# Metabolomic Profiling of Fresh Goji (*Lycium barbarum* L.) Berries from Two Cultivars Grown in Central Italy: A Multi-Methodological Approach

**DOI:** 10.3390/molecules26175412

**Published:** 2021-09-06

**Authors:** Mattia Spano, Alessandro Maccelli, Giacomo Di Matteo, Cinzia Ingallina, Mariangela Biava, Maria Elisa Crestoni, Jean-Xavier Bardaud, Anna Maria Giusti, Alessia Mariano, Anna Scotto D’Abusco, Anatoly P. Sobolev, Alba Lasalvia, Simonetta Fornarini, Luisa Mannina

**Affiliations:** 1 Department of Chemistry and Technology of Drugs, Sapienza University of Rome, Piazzale Aldo Moro 5, 00185 Rome, Italy; mattia.spano@uniroma1.it (M.S.); alessandro.maccelli@uniroma1.it (A.M.); giacomo.dimatteo@uniroma1.it (G.D.M.); cinzia.ingallina@uniroma1.it (C.I.); mariangela.biava@uniroma1.it (M.B.); alba.lasalvia@uniroma1.it (A.L.); simonetta.fornarini@uniroma1.it (S.F.); luisa.mannina@uniroma1.it (L.M.); 2 Institut de Chimie Physique, CLIO, Université Paris Saclay, Bât 200, BP34, CEDEX, 91898 Orsay, France; j.xavier.bardaud@gmail.com; 3 Department of Experimental Medicine, Sapienza University of Rome, P.le Aldo Moro 5, 00185 Rome, Italy; annamaria.giusti@uniroma1.it; 4 Department of Biochemical Sciences, Sapienza University of Roma, P.le Aldo Moro 5, 00185 Rome, Italy; alessia.mariano@uniroma1.it (A.M.); anna.scottodabusco@uniroma1.it (A.S.D.); 5 Institute for Biological Systems, Magnetic Resonance Laboratory “Segre-Capitani”, CNR, Via Salaria Km 29.300, 00015 Monterotondo, Italy

**Keywords:** Goji berries, metabolite profile, NMR, FT-ICR MS, Goji leaves, biological assays

## Abstract

The metabolite profile of fresh Goji berries from two cultivars, namely Big Lifeberry (BL) and Sweet Lifeberry (SL), grown in the Lazio region (Central Italy) and harvested at two different periods, August and October, corresponding at the beginning and the end of the maturation, was characterized by means of nuclear magnetic resonance (NMR) and electrospray ionization Fourier transform ion cyclotron resonance (ESI FT-ICR MS) methodologies. Several classes of compounds such as sugars, amino acids, organic acids, fatty acids, polyphenols, and terpenes were identified and quantified in hydroalcoholic and organic Bligh-Dyer extracts. Sweet Lifeberry extracts were characterized by a higher content of sucrose with respect to the Big Lifeberry ones and high levels of amino acids (glycine, betaine, proline) were observed in SL berries harvested in October. Spectrophotometric analysis of chlorophylls and total carotenoids was also carried out, showing a decrease of carotenoids during the time. These results can be useful not only to valorize local products but also to suggest the best harvesting period to obtain a product with a chemical composition suitable for specific industrial use. Finally, preliminary studies regarding both the chemical characterization of Goji leaves generally considered a waste product, and the biological activity of Big Lifeberry berries extracts was also investigated. Goji leaves showed a chemical profile rich in healthy compounds (polyphenols, flavonoids, etc.) confirming their promising use in the supplements/nutraceutical/cosmetic field. MG63 cells treated with Big Lifeberry berries extracts showed a decrease of iNOS, COX-2, IL-6, and IL-8 expression indicating their significant biological activity.

## 1. Introduction

Goji berries or wolfberries (*Lycium* fruits) are healthy fruits of closely related shrubs from the genus *Lycium*, belonging to the Solanaceae family. *L. barbarum* and *L. chinense* species have a long history in ancestral Chinese medicine as traditional medicinal herbs and food supplements [1]. Beneficial effects on aging, neuroprotection, and well-being such as antioxidant, immunomodulatory, and anticancer properties [2,3,4] have been ascribed to Goji berries’ chemical composition including polysaccharides, terpenoids, glycerogalactolipids, carotenoids, organic acids, phenolic acids (chlorogenic, caffeic acid, *p*-coumaric acids, quercetin, kaempferol) [5], flavonoids, vitamins, minerals, and fibers [6,7,8,9,10]. The beneficial effects contributed are widespread from the consumption of Goji berries as a functional food in many dietary, cosmetic, and pharmaceutical preparations and supplements in the Western countries, including Italy. Italy is one of the main Goji berries producers in Europe [11]. Among the many Goji berries cultivars, Big Lifeberry and Sweet Lifeberry are of particular interest and are widely grown in Europe and North America [12]. Generally, analytical studies on Goji berries have been focused on the chemical characterization of dried berries commercially available in markets. In particular, targeted techniques such as chromatography and UV-Vis spectrophotometry have been applied to study specific classes of compounds such as polyphenols, carotenoids, sugars, organic acids, fatty acids, polysaccharides, and fibers [5,6,9,10,11,13,14].

In this study, Big Lifeberry (BL) and Sweet Lifeberry (SL) Goji berries grown in the South Lazio region (Central Italy) and harvested in August and October [11] were investigated by a multimethodological analytical protocol after their harvesting, before the post-harvesting treatments to obtain a complete metabolite profile. In particular, high-resolution nuclear magnetic resonance (NMR) spectroscopy, electrospray ionization Fourier transform ion cyclotron resonance (ESI FT-ICR) mass spectrometry, and UV-Vis spectrophotometry was applied to obtain the metabolite profile of the hydroalcoholic and organic Bligh-Dyer method extracts. This multimethodological approach has been already exploited to other complex matrices, including essential oils [15] and foodstuffs [16,17,18], ensuring a reliable and extensive phytochemical coverage and quality assessment of the Lycium fruits analyzed from Central Italy. Finally, preliminary studies regarding both the metabolite profile of Goji leaves extracts [19,20] and the biological activity of Big Lifeberry berries was also carried out.

## 2. Results and Discussion

### 2.1. Goji Berries Metabolite Profile

Goji berries Bligh-Dyer extracts from BL and SL Goji berries harvested in August (BL1 and SL1) and October (BL2 and SL2) were analyzed using high-resolution NMR and direct infusion ESI FT-ICR MS methodologies. The ESI FT-ICR MS allowed us to detect many molecular formulas, whereas the less sensitive NMR methodology provided structural and quantitative information. The NMR and ESI FT-ICR assignments will be first discussed separately. Then, the metabolite profile will be discussed for the class of compounds assimilating the results obtained by the two complementary untargeted methodologies. 

#### 2.1.1. ^1^H NMR Assignment

NMR spectroscopy represents a very powerful tool for metabolomic studies since this methodology allows us to resolve complex mixtures such as natural matrices extracts. This is due to the combined interpretation of both one-dimensional and two-dimensional NMR experiments. The ^1^H NMR spectral assignments of hydroalcoholic (Table 1) and organic (Table 2) Bligh-Dyer extracts were carried out by means of 2D experiments and literature data regarding other vegetable matrices analyzed in the same experimental conditions [21,22]. Three sugars, six organic acids, fifteen amino acids, choline, and trigonelline were detected in all the hydroalcoholic Bligh-Dyer extracts and their characteristic ^1^H NMR signals were used for compound quantification. Acetic acid, fumaric acid, arginine, and glutamine were not quantified because of their signals overlapping.

Regarding the organic Bligh-Dyer extracts, β-sitosterol, fatty acids, phosphatidylcholine, digalactosyldiacylglycerol, squalene, and carotenoids were identified in ^1^H NMR spectra of all samples. Squalene was not quantified due to signal overlapping. 

#### 2.1.2. ESI FT-ICR Assignment

ESI(+) FT-ICR mass spectra of hydroalcoholic extracts from BL and SL berries (Figures S1 and S2) showed peaks due to protonated compounds or alkali metal adducts. The list of assigned elemental formulas together with the experimental and theoretical *m/z* ratios, the mass deviation, and the putative compound annotations are available in either ionization mode in Tables S1 and S2 (hydroalcoholic extracts), and Tables S3 and S4 (organic extracts).

The distribution of the identified molecular formulas is reported in Table 3. Numerous compounds assayed by CID-MS^n^ (n = 2,3) experiments provided additional structural information useful to validate peak assignments of likely isomers (Figure S3). Globally, a higher number of molecules were revealed in positive mode, and the hydroalcoholic extracts were relatively richer than the organic ones for each sample.

BL and SL samples collected in August showed a similar chemical profile in both hydroalcoholic and organic extracts. When harvested in October, more metabolites were observed in BL than in SL in hydroalcoholic extracts, whereas the opposite trend was observed in organic extracts.

**Figure 1:**
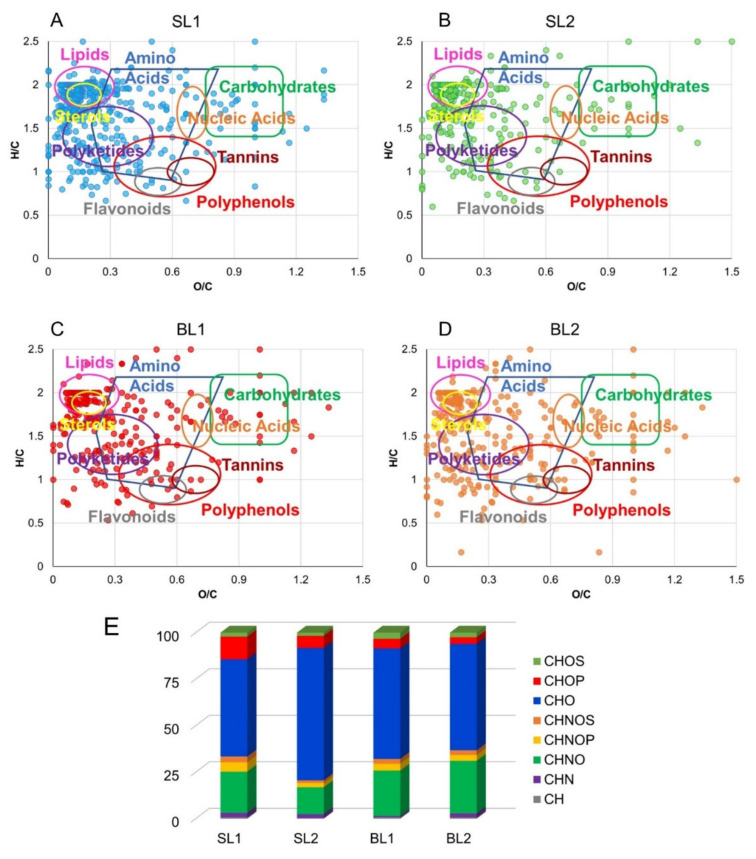
Van Krevelen diagram (elemental plot) obtained from the molecular formulas determined by ESI FT-ICR and Esquire 6000 MS analyses of hydroalcoholic and organic berries extracts of SL1 (panel **A**), SL2 (panel **B**), BL1 (panel **C**), BL2 (panel **D**). Each point represents a distinctive molecular formula. Relative frequency histograms of CH, CHN, CHO, CHNO, CHOP, CHOS, CHNOP, and CHNOS elemental compositions in SL and BL berries extracts (**E**).

A large amount of assigned molecular formulas was visualized by two-dimensional van Krevelen diagrams (vKd) where elemental formulas are sorted based on their H/C versus O/C atomic ratios (Figure 1A–D). These plots enable the identified molecular compositions to be organized, thus providing a qualitative overview of densities of molecular classes, including lipids, amino acids, carbohydrates, polyketides, nucleic acids, and polyalcohols [23]. Regardless of the harvest period, SL and BL cultivars were characterized by strong similarities, with a high density of phytochemicals in the regions of lipids, polyketides, amino acids, and polyphenols.

In addition, homology series along trend lines with specific slopes can be identified as indicative of structural relationships among families of compounds brought about by chemical reactions such as (de)hydrogenation (A-lines), oxidation or reduction (B-lines), (de)hydration (C-lines) and (de)methylation routes (D-lines) [23]. In particular, hits along A-lines comprise, for example, lactic acid (C_3_H_6_O_3_)/glycerol (C_3_H_8_O_3_), fumaric (C_4_H_4_O_4_)/succinic (C_4_H_6_O_4_) acids, for O/C = 1.0; entries along B-lines include cinnamyl alcohol (C_9_H_10_O)/dihydrocinnamic acid (C_9_H_10_O_2_), for H/C = 1.1, and rhamnose (C_6_H_12_O_5_)/glucose (C_6_H_12_O_6_)/gluconic acid (C_6_H_12_O_7_), for H/C = 2.0; items along D-lines encompass palmitic (C_16_H_32_O_2_)/ stearic (C_18_H_36_O_2_)/ arachidic (C_20_H_40_O_2_) acids (Figure S4). 

The relative frequency distribution of the annotated molecular formulas in Figure 1E shows that the most abundant metabolites in all extracts are CHO species, alcohols, carbohydrates, lipids, and polyphenols, ranging from 59% (BL1) to 52% (SL1), and CHNO compounds, mainly amino acids, and alkaloids, from 14% (SL2) to 28% (BL2).

CHOS, CHOP, CHNOP, and CHNOS components were measured in low percentage, with SL1 comprising the majority of CHOP (12%) and CHNOP (5%) groups.

According to Figure 1E, SL1 presented the major biodiversity of CHOP (12%), CHNOP (5%), and CHNOS (3%) components, whereas BL2 contained the highest number of CHN (2%) and CHOS (2%) species. Some metabolites were identified in almost all extracts, such as phosphate, valine, arginine, aldohexose, sucrose, *O*-galloyl-glucose, lactic, aspartic, glutamic, malic, citric, myristic, palmitic, and stearic acids, found in the hydroalcoholic extracts; and limonene, arachidonic, eicosenoic, docosanedioic, linoleic, linolenic, palmitoleic and oleic acids, vitamin D3 derivatives, and mono- and diacylacylglycerols, detected in the organic one. 

Conversely, other phytochemicals were detected only in few samples as hereafter reported in detail for a class of compounds.

#### 2.1.3. Amino Acids and Derivatives

**Figure 2:**
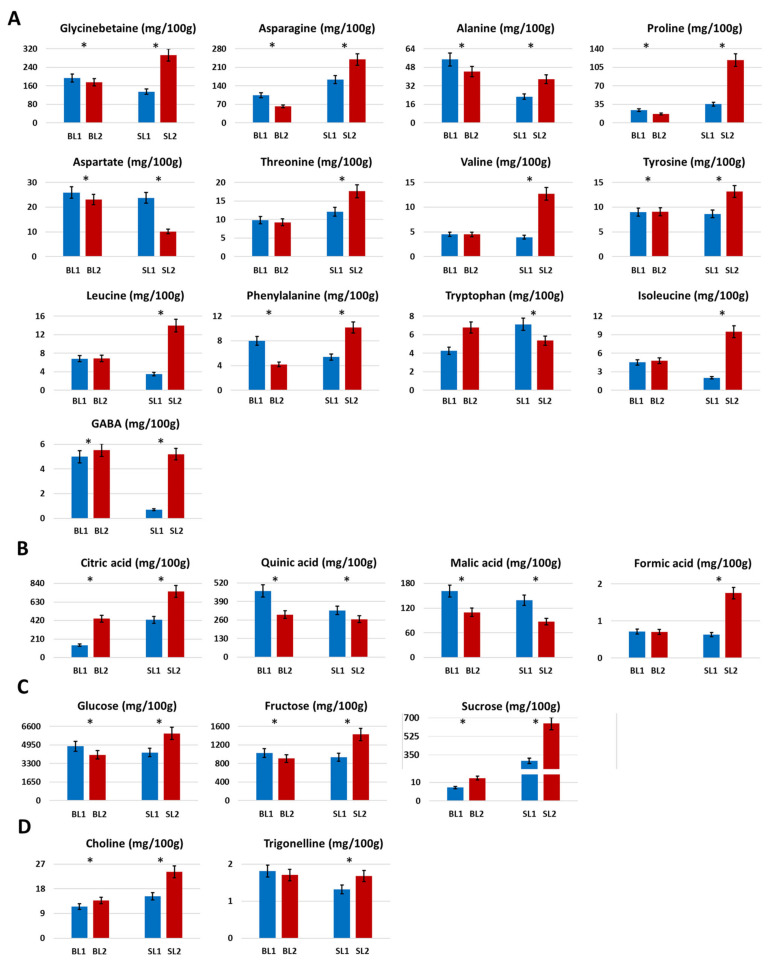
Histograms resulting from the quantitative NMR analysis of quantified compounds present in the Bligh-Dyer hydroalcoholic extracts of Goji berries cultivars Big Lifeberry (BL) and Sweet Lifeberry (SL) harvested in August (BL1, SL1) and October (BL2, SL2). (**A**) Sugars, (**B**) Organic acids, (**C**) Amino acids, (**D**) Other metabolites. Results were expressed as mg/100g ± SD of fresh sample. * *p* < 0.05 significantly difference between the two harvesting periods in the same cultivar, one-way ANOVA.

Glycinbetaine, a typical and characteristic metabolite of Goji berries [2,24] was the most abundant amino acid derivative detected in ^1^H NMR spectra of berries, reaching the highest amount in the SL2 sample, that significantly increased (*p* < 0.05) in August, see Figure 2A. Asparagine was also present in high concentration, with the highest level in the SL cultivar, mainly in October. It was noteworthy that in the BL cultivar, the amounts of amino acids, mainly branched chains (no statistical difference), were generally the same in the two harvesting periods, whereas for the SL cultivar, peculiar trends were observed. Many amino acids such as proline, valine, isoleucine, and GABA significantly increased during the time, whereas tryptophan and aspartate showed a strong decrease from SL1 to SL2 (*p* < 0.05).

ESI FT-ICR MS also detected signals of glutamic acid, lysine, methionine, and histidine, covering almost all the proteinogenic amino acids. Other amino acid derivatives, including proline betaine, acetyltyrosine, nitroarginine, and small peptides, like aspartyl-lysine, glutamyl-carnitine, and prolylproline were also observed. FT-ICR allowed us to identify formally isobaric species, for example, protonated histidine, (*m/z* 156.07654) and potassiated valine, (*m/z* 156.04220) in BL1 (Figure S5). Conversely, histidine was barely present in SL2. *N*-Acetyl-phenylalanine was present only in BL2, whereas lysine discriminated the two cultivars in hydroalcoholic extracts, being present as an intense peak only in BL.

#### 2.1.4. Organic Acids

The presence of citric, quinic, succinic, malic, lactic, and fumaric acids was confirmed by both NMR and ESI(−) FT-ICR MS analyses. For instance, regarding the NMR assignments of some of these metabolites, citric acid was firstly recognized in ^1^H spectra by the typical “roofing” doublets (*J* = 15.4 Hz) at 2.55 and 2.68 ppm of its symmetric diastereotopic CH_2_ groups. The long-range correlation with β-C, external and internal COOH groups at 76.5, 180.1, and 183.0 ppm, respectively, confirmed the identification of this metabolite. Similarly, malic acid was firstly recognized by its typical double doublets in the ^1^H NMR spectra at 2.39, 2.70, and 4.30 ppm, corresponding to α-CH and diastereotopic β-CH protons (Table 1). The ^1^H-^1^H TOCSY experiment allowed us to identify the mutual correlations between these protons; moreover, the ^1^H-^13^C HSQC experiments confirmed the short-range correlation between each proton and their corresponding carbons [25]. According to literature data [26,27], citric and quinic acids were the most abundant organic acids in the Goji berries hydroalcoholic extracts, see Figure 2B, showing a different trend over the season. In particular, citric acid strongly increased (*p* < 0.05) from August to October whereas quinic acid decreased in both cultivars, mainly in BL. Moreover, citric acid was present in major concentrations in SL2, whereas quinic acid was in BL1. Malic acid content decreased over time, with both cultivars having the same concentrations at each harvesting period. Finally, formic acid was always present at low concentrations, increasing in SL2.

ESI(−) FT-ICR mass analysis allowed us to detect malonic acid and caffeic-quinone acid in SL2, 5-hydroxyferulic in BL2, and hydrocinnamic acid in all samples, except in SL2. Chlorogenic acid, already reported in a previous study on Italian Goji berries [10], was detected in BL1 and BL2 samples, as well as chorismic acid, involved in the biosynthetic pathways of aromatic amino acids and alkaloids [28].

#### 2.1.5. Sugars

Sugars turned out to be the most abundant metabolites in hydroalcoholic Bligh-Dyer extracts of Goji berries. Three sugars namely glucose, fructose, and sucrose were assigned and quantified in the NMR spectra of hydroalcoholic extracts. As previously described and in this case, the assignment of sugars, such as all the NMR identified metabolites, was carried out by means of mono and two-dimensional experiment interpretations. For instance, for sucrose identification, its typical doublet at 5.42 ppm which was due to the anomeric CH group of glucose moiety, was recognized in the ^1^H NMR spectra. Proton spin system correlations confirmed the presence of glucose moiety; moreover, the ^1^H-^13^C HMBC experiment allowed us to observe the glucose anomeric group correlation with 104.8 ppm carbon typical of the C-2 fructose group, thus confirming the presence of this disaccharide. Glucose was measured as the most abundant sugar, followed by fructose and sucrose, Figure 2C. Significative variations during the time were observed for the three measured sugars. In particular, in the BL cultivar, a decrease of glucose and fructose was observed during the period, whereas sucrose slightly increased. In the SL cultivar, an increase of the three sugars was observed over time. In particular, it was noteworthy that sucrose was more abundant (at least 40 times) in the SL cultivar with respect to BL. A chromatographic quantification of glucose, fructose, and sucrose in the BL and SL cultivars harvested in Switzerland in September 2004, expressed as dried sample weight, has been reported in previous work [29]. Since the results have been expressed in a different way, a comparison between the absolute quantitative values was not possible. However, in the case of the cultivar harvested in Switzerland, fructose was reported as the most abundant sugar, whereas in the extracts investigated here, glucose turned out to be the most abundant. Moreover, the same level of sucrose has been reported in the BL and SL cultivars conversely to what was observed in the present study.

ESI(−) FT-ICR mass analysis allowed us also to detect mannitol-1-phosphate, only in BL2.

#### 2.1.6. Fatty Acids and Polar Lipids

**Figure 3:**
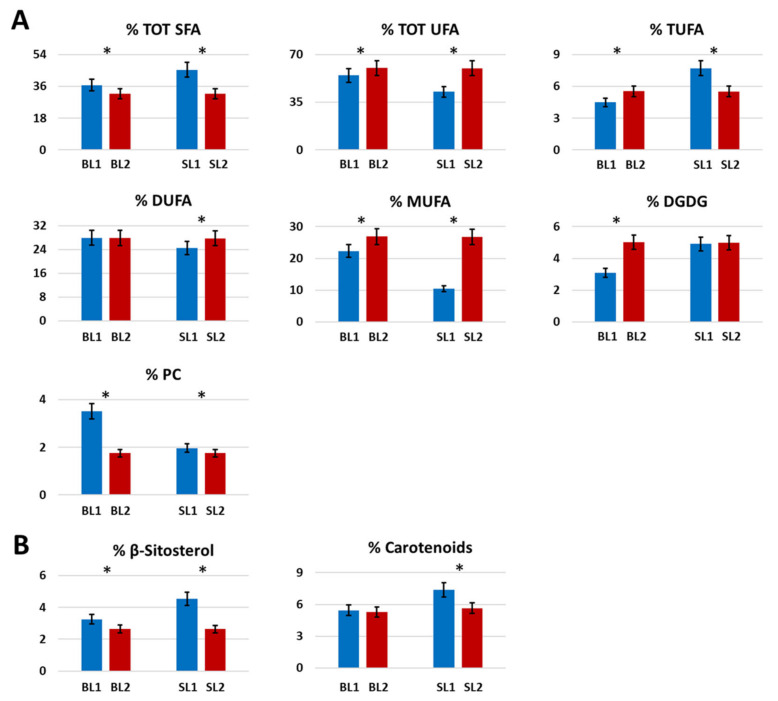
Histograms resulting from the quantitative NMR analysis of some metabolites present in the Bligh-Dyer organic extracts of Goji berries cultivars Big Lifeberry (BL) and Sweet Lifeberry (SL) harvested in August (1) and October (2). (**A**) fatty acids and polar lipids, (**B**) Other metabolites. Results were expressed as molar % ± SD. * *p* < 0.05 significantly difference between the two harvesting periods in the same cultivar, one-way ANOVA.

In organic Bligh-Dyer extracts of Goji berries analyzed with NMR, a significant decrease (*p* < 0.05) during the time of total saturated fatty acids (SFA) was observed in both BL and SL cultivars, whereas total unsaturated fatty acids had an opposite trend, Figure 3A. Regarding the single classes of unsaturated fatty acids, di-unsaturated fatty acids (DUFA) were measured as the most abundant, showing the same content in the BL cultivar and a slight increase in SL. Mono-unsaturated fatty acids (MUFA) generally increased during the time for both cultivars, with the lowest content measured in SL1 that, on the contrary, was characterized by the highest amount of tri-unsaturated fatty acids (TUFA), generally present in low concentrations [30,31]. Regarding polar lipids, the BL cultivar showed an increase of digalactosyldiacylglycerol (DGDG) and a decrease in phosphatidylcholine (PC) during the time. In SL, DGDG and PC maintained the same value from August to October.

While NMR spectroscopy allowed to identify each general class of FAs (TOT SFA, TOT UFA, MUFA, DUFA, TUFA), ESI FT-ICR MS identified the specific FAs for each group. In particular, Goji berries were confirmed to be an important source of essential polyunsaturated FAs, comprising ω-3 (linolenic, stearidonic, and 12-hydroxyeicosatetraenoic) and ω-6 (linoleic, arachidonic, and docosadienoic) acids. Moreover, several species belonging to SFA (from 12:0 to 20:0) and MUFA (from 14:1 to 20:1) were detected in all samples.

#### 2.1.7. Miscellaneous 

Choline slightly increased during the time in both cultivars, whereas the content of trigonelline was quite the same in BL and slightly increased in SL, Figure 2D. β-sitosterol decreased over time in both cultivars whereas carotenoids were present at the same concentration in BL1 and BL2, whereas, in SL, its value significantly decreased (*p* < 0.05) during the time, Figure 3B.

Both cultivars presented numerous metabolites covering the classes of terpenes, such as limonene; terpenoids, such as apiole and phytuberin; hormones (cis-zeatin-*O*-glucoside); flavonoids (myricetin, cyanidin 3-4′′-acetylglucoside, quercetin 3-galactoside [32], flavonol 3-*O*-d-glycoside, apigenin-rhamnoside-rutinoside); vitamins (retinoic acid and pantothenic acid); nucleosides (dimethyladenosine, orotidine, methylguanosine); alkaloids (theophylline, nicotine, and caffeine). Furthermore, a precursor of lignins and lignols (caffeyl alcohol) were also identified in Goji berries, along with kinetin, a phytohormone able to modulate some aging-related disorders in animal models [33], revealed exclusively in the BL cultivar.

### 2.2. Pigments Analysis

In Table 4, the concentrations of chlorophyll a, chlorophyll b, and total carotenoids in Goji berries pulp and peel were reported. The three analyzed pigments were measured in higher concentrations in pulp with respect to peel in all samples. It was noteworthy that the chlorophylls trend was different in the two analyzed cultivars. In particular, chlorophylls a and b increased during the time in BL peel and pulp, whereas in SL, the opposite trend was observed. Moreover, chlorophyll a was measured in higher concentrations than chlorophyll b in the BL cultivar, observing the opposite trend in SL cultivars. On the contrary, the total carotenoids trend was the same in both peel and pulp of the BL and SL cultivars, showing a decrease during the time. According to the NMR results, the highest content of carotenoids was measured in the SL1 sample. Comparing the obtained results with those of a previous work where BL and SL have been cultivated in Switzerland and harvested in September 2014 [29], a different carotenoid content trend was observed. In particular, in the cited work, BL has shown to be richer in carotenoids, of more than 50% than in SL, suggesting how the variation of agronomical factors such as soil, harvesting, and climatic conditions can affect the chemical profile of a natural matrix.

### 2.3. Biological Assays on Berries Extracts and Metabolomic Characterization of Leaves

Preliminary studies of the berries’ biological activities were carried out. In particular, for the preliminary biological assays regarding the anti-inflammatory activity of Goji berries extracts, MG63 osteoblasts were chosen since they are considered a good model to study bone diseases [34], largely widespread in the world population. In these pathologies, the inflammatory pathways are activated as stated by the production of cytokines and chemokines and in turn by secretion of inducible Nitric Oxide Synthase (iNOS) and inducible Cyclooxygenase (COX-2) [35,36]. iNOS and COX-2 generate nitric oxide (NO) and prostaglandins (PGS), respectively, that are involved in many cellular functions as well as in inflammatory settings [37].

Moreover, since Goji leaves are generally considered a waste product, a metabolomic characterization was carried out to define its metabolite profile and valorize a potential use of this matrix in the food field. In particular, both kinds of analyss were carried out considering the BL cultivar in period 1.

#### 2.3.1. Effects of BL1 Goji Berries Extracts on MG63 Cell Viability

The effects of BL1 extracts on MG63 cell viability were determined by the MTS colorimetric method. The extracts did not show detrimental effects at any analyzed concentration, 100 μg/mL, 50 μg/mL, 25 μg/mL, 12.5 μg/mL and 6.5 μg/mL, or time point, 24, 48, and 72 h (Figure S6). For further experiments, 50 μg/mL and 12.5 μg/mL concentrations were used.

#### 2.3.2. Effects of Goji Berries Extracts on Inflammatory Mediators in MG63 Cells

In order to test the effects of the hydroalcoholic and organic extracts of BL1 on inflammatory mediators in MG63 cells, they were added to the cell culture medium at a concentration of 50 and 12.5 μg/mL in presence of 10 ng/mL TNFα. The mRNA expression level of IL-6, IL-8, and IL-1β was analyzed by RT-PCR assay. Both hydroalcoholic and organic extract were able to decrease the examined cytokines mRNA expression level, and this effect is more pronounced in the presence of the organic extract (Figure S7, upper panel). Moreover, the mRNA expression level of iNOS and COX-2 following the pre-treatment with the LB (*L. Barbarum*) extracts, and the stimulus with TNFα was analyzed. A decrease to the basal level of iNOS and COX-2 expression after the treatment with hydroalcoholic and organic extracts with no statistically significant difference between the two extracts was observed (Figure S7, lower panel).

Considering the results obtained on mRNA expression, IL-6 and IL-8 protein production were verified by performing an ELISA assay. Similarly, to the effect on mRNA expression level, LBEHY (hydroalcoholic extract) and LBEORG (organic extract) at the analyzed concentration were able to decrease the production of IL-6 and IL-8 in MG63 cells, Figure S8. 

Taking into account that the mRNA expression of COX-2 was decreased in MG63 cells by LBEHY and LBEORG, we verified whether the extracts were able to reduce the production of this enzyme, performing an immunofluorescence experiment. We found that both LBEHY and LBEORG after 5 h were able to reduce the COX-2 protein expression, Figure S9.

#### 2.3.3. Metabolomic Characterization of Goji Leaves

In order to investigate the potential use of Goji leaves generally considered waste products, a preliminary study of the Goji leaves extracts was carried out. Up to now, the identification of Goji leaves metabolites has been mainly focused on the targeted chromatographic analysis of phenolic compounds [14,20]. In this work, the untargeted metabolomic analysis of Goji leaves was performed to identify as many compound classes as possible. In particular, Bligh-Dyer hydroalcoholic and organic extracts of a leaves pool collected from the Big Lifeberry cultivar in August were investigated by NMR and ESI(+) FT-ICR MS methodologies.

The same NMR experiments (^1^H, ^1^H-^1^H TOCSY, ^1^H-^13^C HSQC, and ^1^H-^13^C HMBC) carried out for the analysis of Goji berries were used for Goji leaves metabolite identification. The ^1^H NMR analysis of leaves extracts allowed us to identify and quantify (see Table 5 and Table 6) the same metabolites present in Goji berries, except for proline, tryptophan, and carotenoids, absent in the leaves. Since the extraction and the NMR experiments were carried out at the same experimental conditions used for berries, the same NMR chemical shifts of Table 1 and Table 2 were identified for metabolites in leaves. In the hydroalcoholic extract, significant quantitative differences were observed comparing leaves and berries, see Table 5: the content of alanine, asparagine, and citric acid was very low in leaves with respect to berries, as well the glucose and fructose levels were at least 10 times lower in leaves than in berries. On the contrary, the sucrose content was measured in high amounts, over 50 times, with respect to berries. Similarly, GABA and malic acid were present in leaves in a higher amount with respect to berries. Betaine was present in a significant amount also in the Goji leaves. Among the other metabolites, comparative values were measured in both the leaves and berries. Regarding the organic extract, TUFAs represented the main class of unsaturated fatty acids in leaves, whereas in berries, they were present in very low concentrations. On the contrary, in leaves, DUFAs were present in very low concentrations and MUFAs were not measured since the error associated with Equation (4) (Section 3.4 of Materials and Methods) turned out to be higher than the MUFA value [18]. Since the quantification of organic extract metabolites is expressed relative to the sum of total metabolite intensities, a comparison between berries and leaves organic metabolites values can not be performed due to the absence of carotenoids in leaves.

The same experimental conditions used for the ESI FT-ICR MS analysis of Goji berries were applied for leaves characterization. Surprisingly, the ESI FT-ICR MS analysis of Goji leaves extracts allowed to identify in both hydroalcoholic and organic extracts, a higher number of molecular formulas with respect to the corresponding BL1 sample. In particular, the analysis of the hydroalcoholic extract allowed the identification of 286 (vs. 96 in berries) and 100 (vs. 114 in berries) molecular formulas in positive and negative mode, respectively. For the organic extract, 202 (vs. 55 in berries) and 84 (vs. 65 in berries) molecular formulas were identified in positive and negative mode, respectively. The list of assigned elemental formulas together with the experimental and theoretical *m/z* ratios, the mass deviation, and the putative compound annotations are available in either ionization mode for Goji leaves in Tables S5 and S6 (hydroalcoholic extracts), and S7 and S8 (organic extracts). Interestingly, the BL1 leaves presented a rich variety of phytochemicals, laying the basis for the helpful exploitation of such waste material.

As an example, the ESI(+) FT-ICR mass spectrum of BL1 leaves hydroalcoholic extract is reported in Figure S10.

**Figure 4:**
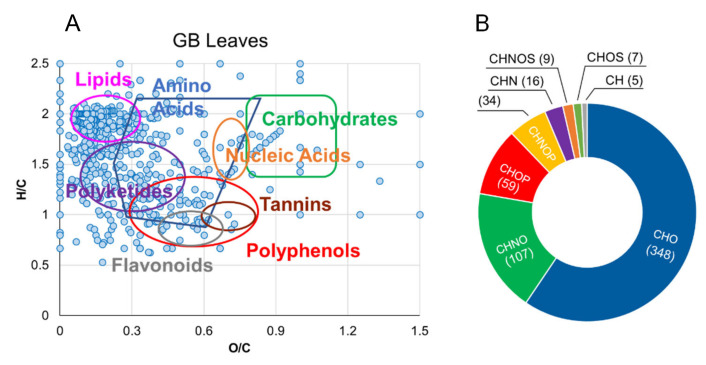
Van Krevelen diagram obtained from the molecular formulas determined by ESI FT-ICR analyses of hydroalcoholic and organic Big Lifeberry leaves extracts (panel **A**); donut chart describing the elemental composition concerning the same extracts (panel **B**).

In this study, almost 600 different molecular formulas/putative compounds were identified, distributed between hydroalcoholic (364) and organic (244) leaf extracts. More entries were collected for both portions in positive ionization mode. Similar to berries, Goji leaves’ extracts cover several molecular classes in van Krevelen diagrams (Figure 4A), with a higher density of entries in the lipids, polyketides, amino acids, and polyphenols areas, and less represented carbohydrates, tannins, and nucleic acids. Accordingly, the relative frequency distribution donut chart of the elemental formulas shown in Figure 4B revealed CHO features (terpenoids, polyphenols, fatty acids, and carbohydrates) as the most populated class, similar to what was observed in the corresponding berries sample, followed by CHNO (amino acids and alkaloids) compounds. Interestingly, a high number of phosphorus-containing metabolites were found as CHOP (59 hits) and CHNOP (34 hits) components, measured in a relatively lower amount in berries. In these classes, phosphorylated-sugars, -amino acids, and -lipids were detected as key intermediates in the primary metabolic processes of the plant. Similar to what was observed above for goji berries, the homology series emerge (Figure S4) along trend A-lines, including entries for oleic (C_18_H_34_O_2_)/linoleic (C_18_H_32_O_2_)/linolenic (C_18_H_30_O_2_) acids; B-lines, with hits for palmitic (C_16_H_32_O_2_)/hydroxyhexadecanoic (C_16_H_32_O_3_)/dihydroxyhexadecanoic (C_16_H_32_O_4_) acids, and succinic (C_4_H_6_O_4_)/malic (C_4_H_6_O_5_)/tartaric (C_4_H_6_O_6_) acids; D-lines, with items for asparagine (C_4_H_8_N_2_O_3_)/glutamine (C_5_H_10_N_2_O_3_), and lauric (C_12_H_24_O_2_)/myristic (C_14_H_28_O_2_)/palmitic (C_16_H_32_O_2_)/stearic (C_18_H_36_O_2_)/arachidic (C_20_H_40_O_2_) acids.

As shown in Figure 4A, the lipids region comprised saturated (primarily 16:0), monounsaturated (mainly 18:1), ω-3 including hexadecatetraenoic, stearidonic and linolenic, and ω-6 like linoleic, arachidonic, and eicosadienoic acids identified as free fatty acids. 

In the polyphenol region, the annotated metabolites could be grouped into two main subsets of tannins, with gallic acid and flavonoids, with anthocyanin, apigenin, myricetin-(galloylrhamnoside), naringenin-(*p*-coumaroylglucoside), isoquercetin, and rutin, already identified as a dominant component in Goji leaves [20]. According to previous evidence, flavonoids glycosidessuch as isoquercetin (quercetin-3-*O*-glucoside) were also found [14]. All these metabolites, mainly present in the hydroalcoholic extract, play significant health-promoting and anticancer activities. The organic fraction from Goji leaves exhibited peaks assigned to protonated and sodiated forms of pheophytin a and chlorophylls (Figure S10), whose potential applications in the food industry and pharmacology have been amply described [38].

Other bioactive compounds responsible for radical scavenging, UV protective and anti-inflammatory effects, like carnosic acid, resveratrol [39], hydroxycinnamic (caffeic, ferulic, chlorogenic) acids [20,40] were also unveiled, concurring to describe the chemical space/metabolic profile of Goji leaves as a valuable source of bioactive/beneficial compounds.

## 3. Materials and Methods

### 3.1. Chemicals

Methanol (HPLC-grade), chloroform (HPLC-grade), and acetonitrile (HPLC-grade) were obtained from Carlo Erba Reagenti (Milan, Italy). Double-distilled water was obtained using a Millipore Milli-Q Plus water treatment system (Millipore Bedford Corp., Bedford, MA, USA). Deuterated water (D_2_O) 99.97 atom% of deuterium, methanol-D4 99.80 atom% of deuterium, and chloroform-D 99.80 atom% of deuterium + 0.03% tetramethylsilane (TMS) was purchased from Euriso-Top (Saclay, France). 3-(trimethylsilyl)-propionic-2,2,3,3-d_4_ acid sodium salt (TSP) was purchased from Merck (Milan, Italy).

### 3.2. Plant Material

Goji berries of two cultivars, namely Big Lifeberry and Sweet Lifeberry, of *Lycium barbarum* L. were grown in the South Lazio area of Subiaco (41°55′34.28′′ N 13°05′20.62′′ E). Fruits were collected in August (period 1, BL1, SL1) and October (period 2, BL2, SL 2), corresponding at the beginning and the end of the maturation period [2,26]. The berries of both cultivars were manually collected from the whole plant, they were homogenized and frozen using liquid nitrogen and, finally, they were stored at −80 °C until analysis.

### 3.3. Extraction Procedure

The Bligh-Dyer extraction protocol [41] was applied for both NMR and ESI FT-ICR analyses, in order to extract both hydrophilic and hydrophobic metabolites. In particular, 1.0 g of homogenized sample was added sequentially with 3 mL methanol/chloroform (2:1 *v/v*) mixture, 1 mL of chloroform, and 1.2 mL of bidistilled water, shaking the sample after each addition. The obtained emulsion was maintained at 4 °C for 40 min and then centrifuged at 4200× *g* for 15 min at 4 °C. Both phases, hydroalcoholic and organic, were carefully separated. The pellets were re-extracted using half of the solvent volumes in the same conditions described above. Finally, hydroalcoholic and organic fractions were dried using an N_2_ flow at room temperature and the dried phases were stored at −20 °C until further analyses.

### 3.4. NMR Analysis

Dried Bligh-Dyer hydroalcoholic extracts were solubilized in 750 µL 400 mM phosphate buffer/D_2_O, containing TSP 1 mM as internal standard, whereas dried Bligh-Dyer organic extracts were solubilized in 700 µL of a CDCl_3_/CD_3_OD mixture (2:1 *v*/*v*). NMR analyses were carried out by using a Bruker AVANCE 600 spectrometer (Bruker, Milan, Italy) operating at the frequency ^1^H frequency of 600.13 MHz and equipped with a multinuclear z-gradient 5 mm probe head (Bruker, Milan, Italy). Mono-dimensional (^1^H) and two-dimensional (^1^H-^1^H TOCSY, ^1^H-^13^C HSQC, and ^1^H-^13^C HMBC) experiments were carried out using the same acquisition and processing parameters previously reported [21]. Two-dimensional experiments allowed us to identify long-range ^1^H-^1^H, and long- and short-range ^1^H-^13^C correlations within each considered molecule. This information, combined with data obtained from mono-dimensional spectra (chemical shift, multiplicity, coupling *J* constants), allowed us to define the chemical structure of the starting unknown compound and define its identity, also by comparing the obtained data with the literature data.

The complete procedures from the extraction to NMR measurement were repeated three times in order to evaluate the repeatability of the protocol. The integrals of 23 signals in hydroalcoholic extracts ^1^H spectra were selected, Table 1, and used for metabolite quantification, as mg/100 g fresh weight sample ± standard deviation (SD), referring to the internal standard TSP. The integrals of 8 signals in organic extracts ^1^H spectra, referred to the area of total fatty acids signal set to 100, were selected, Table 2, and used for metabolite quantification as molar % ± SD by applying the following equations:

%β-SIT = 100(0.66I_β-SIT_ /I_tot_) (1)

%TUFA = 100(0.5I_TUFA_/I_tot_) (2)

%DUFA = 100(I_DUFA_/I_tot_) (3)

%MUFA = 100(I_TOT UFA_ – 2I_DUFA_ – 1.5I_TUFA_)/I_tot_ (4)

%TOT FA = 100(I_TOT FA_/I_tot_) (5)

%TOT UFA = %MUFA + %DUFA + %TUFA(6)

%TOT SFA = %TOT FA – %TOT UFA(7)

%PC = 100(4I_PC_/9I_tot_) (8)

%DGDG = 100(4I_DGDG_ /I_tot_) (9)

%CAR = 100(I_CAR_ /I_tot_) (10)

where %β-SIT, %TUFA, %DUFA, %MUFA, %TOT FA, %TOT UFA, %TOT SFA, %PC, %DGDG, and %CAR are molar % of β-sitosterol, tri-unsaturated fatty acids, di-unsaturated fatty acids, mono-unsaturated fatty acids, total fatty acids, total unsaturated fatty acids, total saturated fatty acids, phosphatidylcholine, digalactosyldiacylglycerol, and carotenoids respectively. I_β-SIT_, I_TUFA_, I_DUFA_, I_TOT UFA_, I_TOT FA_, I_PC_, I_DGDG_, and I_CAR_ are integrals, whereas I_tot_ is calculated according to the following equation:

I_tot_ = I_TOT FA_ + 0.66I_β-SIT_ + I_CAR_ (11)

One-way analysis of variance (ANOVA) was realized by Statistica software (Version 14.0.0.15) in order to determine significant differences (*p* < 0.05) among the quantified metabolites at different harvesting stages for each goji berry cultivar.

### 3.5. Mass-Spectrometry-Based Metabolomics

Stock solutions (1 mg/mL) of Bligh-Dyer hydroalcoholic and organic extracts of BL and SR fruits and BL leaves were filtered through a 0.45 μm hydrophobic polypropylene Acrodisc (VWR, Milan, Italy) to eliminate debris, and diluted in methanol and methanol/acetonitrile (50:50 *v/v*), respectively, to a final concentration of 0.2–0.5 mg L^−1^. No ionization dopants were added. In order to avoid degradation, the stock solutions were kept at −20 °C and analyzed within 24 h after final dilution. Each diluted solution was directly infused with a flow rate of 120 µL/h in the electrospray ionization source of the mass spectrometer for untargeted analysis. All the analyses and Collision Induced Dissociation (CID) experiments were conducted on mass-selected ions with He gas using either a Paul ion trap (Esquire 6000, Bruker Daltonics, Bremen, Germany) or an LTQ XL (Thermo Scientific) in both positive (ESI(+)) and negative (ESI(−)) polarity modes. ESI settings were set as follows: solution flow rate of 120 µL/h, capillary spray voltage at –4.2 kV (in positive mode) or +3.8 kV (in negative mode), nebulizer pressure at 11 psi, drying gas flow at 6 L/min, and drying gas temperature at 300 °C. To gather proof of peak assignments, CID on mass-selected ions obtained from commercially available standards were carried out and further tested by reference spectra obtained from the literature or online libraries, like METLIN [42]. High-resolution mass analysis was carried out using a Fourier Transform-Ion Cyclotron Resonance (FT-ICR) mass spectrometer (Bruker Daltonics GmbH, Bremen, Germany) equipped with an Apollo I ESI source, a 4.7 T superconducting magnet, and an infinity cell (FT-ICR lab, Sapienza University of Rome, Rome, Italy) [43]. A methanolic 10 µM solution of leucine enkephalin (YGGFL, C_28_H_37_N_5_O_7_, molecular weight: 556.26930) was added as an internal standard for precision and mass accuracy assessment. Additional internal frequency-to-*m/z* calibration was attained by relating to ubiquitous metabolites of known elemental composition, like arginine, monosaccharides, and organic acids. FT-ICR mass spectra were typically acquired in the *m/z* 90–1000 range (resolution of 70,000 at *m/z* 500) in at least three replicates. For each sample, the time domain signal was collected by co-adding 200 scans with an acquisition size of 1M. 

### 3.6. Metabolomics Visualization Tools

The list of *m/z* values was subjected to preliminary filtering by retaining only peaks with a cut-off signal-to-noise ratio (S/N) of 3 and then submitted to the free tool MassTRIX [44] for metabolite assignment, by considering protonated, sodiated, and potassiated (ESI(+)), and deprotonated and chlorinated (ESI(−)) ions. The components of the extracts were assigned by comparing the high-resolution *m/z* values with the theoretical exact mass. All metabolite annotations were based on the “monoisotopic” ion. A qualitative description of the phytochemical composition of the samples has been gained by visualization tools, including van Krevelen diagrams (vKds) and relative elemental composition frequency histograms [17].

### 3.7. Spectrophotometric Pigments Analysis

Total carotenoids and chlorophylls were analyzed in the peel and pulp of the Goji berry varieties by using a previously described protocol [45]. In particular, the peel was cut from the surface of the fruits and carefully freed from the pulp. Samples were homogenized with mortar and pestle in 6 mL of chloroform/methanol mixture (2:1, *v/v*) and 30 mg of MgO to prevent chlorophyll pheophytinization. The obtained homogenate was filtered on a paper filter and then a volume of distilled water equal to 20% of the extract volume was added. Finally, the obtained biphasic system was centrifuged for 20 min at 2469× *g* at 10 °C to complete the separation of chloroform fraction from hydroalcoholic one. Absorption spectra of the chloroform phase were acquired with Beckman Coulter DU 800 instruments, in the range of 350–800 nm with a spectral resolution of 0.5 nm at 20 °C. The concentrations of chlorophyll a and b, as well as total carotenoids, were expressed as mg/g ± SD of fresh material were determined according to Wellburn equations [46].

### 3.8. Biological Assays

MG-63 osteosarcoma cell line, obtained from the American Type Culture Collection (ATCC, Rockville, MD, USA) was used as an osteoblast model. The cells were grown in Dulbecco’s modified eagle medium Glutamax (Sigma, St. Louis, MI, USA), supplemented with 10% fetal bovine serum (GIBCO, Amarillo, TX, USA) with penicillin (100 μg/mL), streptomycin (100 U/mL), and 1% sodium pyruvate, at 37 °C with 5% CO_2_, for 24 h, reaching a confluence of around 80%.

#### 3.8.1. Cell Viability

To assess a potential cytotoxic effect of hydroalcoholic and organic extracts of BL1 on MG63 cells at different concentrations and time points, an MTS (3-[4,5-dimethylthiazol-2-yl]-5-[3-carboxymethoxyphenyl]-2-[4-sulfophenyl]-2*H*-tetrazolium)-based colorimetric assay was performed (Promega Corporation, Madison, WI, USA). Briefly, 5 × 10^3^ cells per well were seeded in a 96-well plate. The day after seeding, cells were either left untreated (CTL) or treated with different concentrations of hydroalcoholic and organic extract of BL1 for 24, 48, and 72 h. After each time point, 100 μL MTS solution was added to the wells. Spectrophotometric absorbance was directly measured at 492 nm after 3 h incubation. MG63 cells were left untreated (CTL) or treated with 50 or 12.5 μg/mL of hydro-alcoholic and organic extract of *Lycium barbarum* and stimulated with TNFα (PeproTech House, London, UK) 10 ng/mL for the required time. Experiments were independently repeated at least three times.

#### 3.8.2. Quantitative Real-Time PCR

Total RNA was extracted with a Total RNA extraction mini kit (Fisher Molecular Biology, Trevose, PA, USA), and reverse transcribed by Improm II enzyme, (Promega Corporation, Madison, WI, USA), according to the manufacturers’ instructions. Quantitative real-time PCR analysis was performed using an ABI Prism 7300 (Applied Biosystems, Thermo Fisher Scientific, Waltham, MA, USA, accessed on 30 October 2019. Amplification was carried out using the SensimixPlus SYBR master mix (Bioline, London, UK). Primers were designed using Primer Express software (Applied Biosystems, accessed on 30 October 2019) and were synthesized by Biofab Research (Rome, Italy). 

Data were analyzed by the 2^−ΔΔCt^ method, which determines the transcript abundance relative to the 18S housekeeping gene.

#### 3.8.3. ELISA Assay

The amount of Interleukin -6(IL-6) and Interleukin-8 (IL-8) in the supernatant of cell cultures was determined using Enzyme-Linked Immunoadsorbent assay kits (Fine Test ELISA, Fine Biotech Co., Ltd., Wuhan, China) according to the manufacturer’s instructions. Optical Density (O.D.) absorbance was measured at 450 nm by a microplate reader (Appliskan, Thermo Fisher, Waltham, MA, USA). The samples were analyzed after 5 h culture.

#### 3.8.4. Immunofluorescence Measures

Cox-2 was visualized by immunofluorescence. Cells were fixed with 100% ethanol for 10 min at RT. After washing with PBS, cells were incubated with anti-Cox-2 mouse monoclonal antibody (Abcam) 1:250 for 1 h at RT. After washing with PBS, cells were incubated for 1 h at room temperature with Alexa Fluor 568 donkey anti-mouse antibody 1:400 (Invitrogen, Thermo Fisher Scientific), to stain Cox-2 in red. Cells were washed, incubated with DAPI (Invitrogen, Thermo Fisher Scientific) to visualize the nuclei, mounted, and analyzed with Leica DM microscope (Leica Microsystems, Milan, Italy).

## 4. Conclusions

In this work, for the first time, to our knowledge, the metabolite profile of Goji berries obtained using high-resolution NMR and ESI FT-ICR MS untargeted methodologies was reported. This approach contributed to enrich the knowledge of the chemical composition of this fruit by confirming the presence of carotenoids, polyphenols, fatty acids, and sugars; and by identifying other metabolite groups such as amino acids, terpenes, alkaloids, and chlorophylls. The rich phytochemical profile in both SL and BL cultivars confirmed Goji berries as a “superfruit” with remarkable potential bioactivity since the identified molecules are well known for their antioxidant, antimicrobial activities, together with a modulation activity of several metabolic biochemical processes of the organism. Moreover, the information obtained by the analysis of the considered Goji cultivars grown in Italy and harvested at two different ripening periods can be useful to produce Goji fruits with a peculiar chemical profile, since significative compositional differences were observed.

The preliminary biological studies represent a starting point to further characterize the biological effects of Goji berries, whose potential activities represent an important field of research for several applications of pharmaceutical/nutraceutical interest. Finally, the metabolite characterization of Goji leaves showed how this matrix, generally considered as a waste product, can be valorized as a raw material for several applications in the biological and food fields. Of course, further characterization of this matrix is still needed.

## Figures and Tables

**Table 1:** Metabolites identified in the 600.13 MHz ^1^H-NMR spectra of the Bligh-Dyer hydroalcoholic extracts of Goji berries dissolved in 400 mM phosphate buffer/D_2_O containing TSP 1 mM. Signals selected for quantitative analysis (integration) are labeled with an asterisk (*).

Metabolite	Group	^1^H (ppm)	Multiplicity [*J*(Hz)]	^13^C (ppm)
**Sugars**				
α-D-Fructofuranose ^b,l^	C-2			105.4
	CH-3	4.12 *		83.0
	CH-4	4.01		
	CH-5	4.07		82.5
β-D-Fructofuranose ^b,l^	C-2			102.9
	CH-3	4.12 *		76.5
	CH-4	4.12 *		75.4
	CH-5	3.83		
β-D-Fructopyranose ^b,l^	C-2			99.3
	CH-3	3.80		68.6
	CH-4	4.00		77.0
	CH_2_-6,6′	3.72; 4.03		64.4
α-Glucose ^b,l^	CH-1	5.25 *	d [3.8]	93.2
	CH-2	3.55		72.0
	CH-3	3.72		73.3
	CH-4	3.42		70.7
	CH-5	3.84		72.5
	CH_2_-6,6′	3.89; 3.78		61.8
β-Glucose ^b,l^	CH-1	4.66 *	d [8.0]	96.9
	CH-2	3.26		75.1
	CH-3	3.51		76.9
	CH-4	3.42		70.7
	CH-5	3.49		76.8
	CH_2_-6,6′	3.90; 3.75		61.8
Sucrose ^b,l^	CH-1 (Glucose)	5.42 *	d [3.8]	93.1
	CH-2	3.57		71.8
	CH-3	3.77		73.6
	CH-4	3.48		70.2
	CH-5	3.85		73.5
	C-2 (Fructose)			104.8
	CH-3′	4.23	d [8.8]	77.4
	CH-4′	4.07		75.1
	CH-5′	3.90		82.4
	CH_2_-6′	3.83		61.2
**Organic acids**				
Acetic acid ^b,l^	α-CH_3_	1.93	s	24.4
	COOH			182.6
Citric acid ^b,l^	α,γ-CH	2.55 *	d [15.4]	46.6
	α’,γ’-CH	2.68	d [15.4]	46.6
	β-C			76.5
	1,5-COOH			180.1
	6-COOH			183.0
Formic acid ^b,l^	HCOOH	8.47 *	s	
Fumaric Acid ^b,l^	α,β-CH=CH	6.53	s	
Malic acid ^b,l^	α-CH	4.30 *	dd [9.9; 3.2]	71.3
	β-CH	2.70	dd [15.4; 3.2]	43.5
	β’-CH	2.39	dd [15.4; 9.9]	43.5
Quinic acid ^b,l^	CH_2_-2,2′	1.88 *; 2.07	dd [13.5; 10.8]; m	41.7
	CH-3	4.16		
**Amino acids**				
Alanine ^b,l^	α-CH	3.80		51.7
	β-CH_3_	1.49 *	d [7.3]	17.1
	COOH			177.1
Arginine ^b,l^	α-CH	3.79		55.3
	β-CH_2_	1.91	m	28.6
	γ-CH	1.67	m	25.1
	γ’-CH	1.74	m	25.1
	δ-CH_3_	3.24		41.6
Asparagine ^b,l^	α-CH	4.02		52.4
	β,β’-CH_2_	2.89 *; 2.96	dd [7.3; 16.9]	35.5
	γ-CONH_2_			175.4
Aspartate ^b,l^	α-CH	3.94		53.1
	β,β’-CH_2_	2.70; 2.81 *	dd [3.9; 17.4]	37.5
	γ-COO^-^			179.9
GABA ^b,l^	α-CH_2_	2.30 *	t [7.4]	35.4
	β-CH_2_	1.91		25.0
	γ-CH_2_	3.02		41.0
Glycinebetaine ^b,l^	N(CH_3_)_3_+	3.27 *	s	54.4
	α-CH_2_	3.90		67.5
Glutamine ^b,l^	α-CH	3.79		55.4
	β,β’-CH_2_	2.14	m	27.5
	γ-CH	2.46	m	31.9
Isoleucine ^b,l^	β-CH	1.99		37.1
	γ-CH_3_	1.27		25.8
	γ-CH_3_	1.01 *	d [7.1]	15.7
	δ-CH_3_	0.95		12.0
Leucine ^b,l^	β-CH_2_	1.74		40.8
	γ-CH	1.71		
	δ-CH_3_	0.97 *	d [6.2]	23.0
	δ’-CH_3_	0.96	d [6.2]	22.0
Phenylalanine ^b,l^	CH-2,6	7.34	m	130.3
	CH-4	7.38	m	128.6
	CH-3,5	7.43 *	m	130.2
Proline ^b^	α-CH	4.15		62.3
	β,β’- CH_2_	2.07, 2.35		30.3
	γ-CH_2_	2.01 *	m	24.8
	δ,δ’- CH_2_	3.35, 3.42		47.2
Threonine ^b,l^	α-CH	3.61		61.6
	β-CH	4.27		67.2
	γ-CH_3_	1.34 *	d [6.6]	20.4
Tyrosine ^b,l^	CH-3,5	7.20	d [8.5]	131.8
	CH-2,6	6.90 *	d [8.5]	116.7
Tryptophan ^b^	CH-4	7.27	m	
	CH-5	7.19	m	
	CH-6	7.73	d [8.0]	119.5
	CH-7	7.54 *	d [8.0]	113.0
Valine ^b,l^	α-CH	3.62		61.4
	β-CH	2.28		30.3
	γ-CH_3_	1.00	d [7.1]	17.7
	γ’-CH_3_	1.05 *	d [7.1]	18.9
**Miscellaneous metabolites**				
Choline ^b,l^	N(CH_3_)_3_^+^	3.21 *	s	54.8
	α-CH_2_	3.81		68.5
Trigonelline ^b,l^	CH-1	9.13 *	s	
	CH-3,5	8.84		
	CH-4	8.09		

^b^ Metabolite identified in Bligh-Dyer hydroalcoholic extract of Goji berries; ^l^ Metabolite identified in Bligh-Dyer hydroalcoholic extract of Goji leaves.

**Table 2:** Metabolites identified in the 600.13 MHz ^1^H-NMR (Nuclear Magnetic Resonance) spectra of the Bligh-Dyer organic extracts of Goji berries dissolved in CDCl_3_/MeOD 2:1 *v/v* mixture. Signals selected for quantitative analysis (integration) are labeled with an asterisk (*).

Metabolite	Group	^1^H (ppm)	Multiplicity [*J* (Hz)]	^13^C (ppm)
Oleic fatty chain ^b,l^	COO			174.5
(C18:1 Δ^9^)	CH_2_-2	2.30 *		34.6
	CH_2_-3	1.59	m	25.4
	CH_2_-4-7	1.30	m	29.5
	CH_2_-8	2.03	m	27.6
	CH=CH-9,10	5.30 *	m	128.4
	CH_2_-11	2.03	m	27.6
	CH_2_-12-15	1.33–1.30	m	29.5–29.9
	CH_2_-16	1.29	m	29.7
	CH_2_-17	1.24	m	32.2
	CH_3_-18	0.84	t	14.1
Linoleic fatty chain ^b,l^	COO			174.5
(C18:2 Δ^9,12^)	CH_2_-2	2.30 *		34.6
	CH_2_-3	1.59	m	25.4
	CH_2_-4-7	1.32–1.28	m	29.5
	CH_2_-8	2.03	m	27.6
	CH= 9	5.33 *	m	130.5
	CH= 10	5.30 *	m	128.4
	CH_2_-11	2.75 *	t [6.8]	25.9
	CH= 12	5.30 *	m	128.4
	CH= 13	5.33 *	m	130.5
	CH_2_-14	2.03	m	27.6
	CH_2_-15	1.29	m	29.7
	CH_2_-16	1.29	m	29.7
	CH_2_-17	1.24	m	32.2
	CH_3_-18	0.86	t	14.1
Linolenic fatty chain ^b,l^	COO			174.5
(C18:3 Δ^9,12,15^)	CH_2_-2	2.30 *		34.6
	CH_2_-3	1.57	m	25.4
	CH_2_-4-7	1.30	m	29.5
	CH_2_-8	2.04	m	27.5
	CH=CH 9,10	5.33 *	m	128.4
	CH_2_ 11	2.79 *	t [6.2]	25.9
	CH=CH 12,13	5.33 *	m	128.4
	CH_2_-14	2.79 *	t [6.2]	25.9
	CH= 15	5.28 *	m	128.6
	CH= 16	5.35 *	m	132.2
	CH_2_-17	2.04	m	20.6
	CH_3_-18	0.95	t [7.5]	14.3
Saturated fatty acids ^b,l^	COO			174.5
	CH_2_-2	2.28 *		34.8
	CH_2_-3	1.57	m	25.4
	CH_2_	1.28–1.22	m	29.9–32.0
	CH_2_ n-1	1.25		23.0
	CH_3_ n	0.84	t	14.1
β-Sitosterol ^b,l^	C-13			42.4
	CH-17	1.16		56.4
	CH_3_-18	0.66 *	s	12.0
Squalene ^b,l^	CH_3_-a	1.56		
	CH_3_-b	1.65		
	CH-c	5.08	m	124.7
	CH_2_-d	2.04		
	CH_2_-e	1.96		
1,2-Diacyl-*sn*-glycero-3-Phosphatidylcholine ^b,l^	CH_2_-*sn1*	4.30, 4.14		62.5
	CH-*sn2*	5.22		70.7
	CH_2_-*sn3*	3.94, 3.70		68.2
	N(CH_3_)_3_+	3.19 *	s	54.4
Digalactosyldiacylglycerol ^b,l^	CH_2_-*sn1*	4.30, 4.14		62.5
	CH-*sn2*	5.06		70.7
	CH_2_-*sn3*	3.66		68.2
	CH’’-1	4.87 *	d [3.8]	99.7
	CH’’-2	3.78		68.5
	CH’’-3,5	3.70		70.6
	CH’’-4	3.92		70.1
	CH’-1	4.20		104.3
	CH’-2	3.50		71.3
	CH’-3	3.47		73.5
	CH’-4	3.87		66.6
Carotenoids ^b^	CH-7	6.09		125.6
	CH-8	6.09		139.2
	CH-10	6.14		131.7
	CH-11	6.63		125.2
	CH-12	6.35 *		138.0
	CH-14	6.24		133.0
	CH-15	6.62		130.4

^b^ Metabolite identified in Bligh-Dyer hydroalcoholic extract of Goji berries; ^l^ Metabolite identified in Bligh-Dyer hydroalcoholic extract of Goji leaves.

**Table 3:** Molecular formulas (MF) identified by electrospray ionization-mass spectrometry (ESI-MS) in Bligh-Dyer hydroalcoholic and organic extracts of Big Lifeberry (BL) and Sweet Lifeberry SL) berries harvested in periods 1 and 2.

Extract	Hydroalcoholic ^a^			Organic ^b^		
	Ion Mode	Identified MF		Ion Mode	Identified MF	
BL1	ESI(+)	96	191	ESI(+)	55	109
	ESI(−)	114		ESI(−)	65	
BL2	ESI(+)	118	219	ESI(+)	71	104
	ESI(−)	121		ESI(−)	38	
SL1	ESI(+)	165	273	ESI(+)	114	169
	ESI(−)	114		ESI(−)	63	
SL2	ESI(+)	93	138	ESI(+)	101	170
	ESI(−)	57		ESI(−)	84	

^a^ Investigated by means of ESI FT-ICR MS. ^b^ Investigated by means of ESI Ion trap MS.

**Table 4:** Chlorophyll a, chlorophyll b, and total carotenoid concentrations by spectrophotometric analysis (mg/g of fresh sample ± SD).

		Chlorophyll a	Chlorophyll b	Tot Carotenoids
BL1	Pulp	0.0112 ± 0.0011	0.0214 ± 0.0012	1.4210 ± 0.0452
	Peel	0.0043 ± 0.0008	0.0083 ± 0.0002	0.5300 ± 0.0340
BL2	Pulp	0.0195 ± 0.0015	0.0320 ± 0.0018	1.1980 ± 0.0420
	Peel	0.0139 ± 0.0018	0.0281 ± 0.0014	0.3274 ± 0.0285
SL1	Pulp	0.0300 ± 0.0080	0.0589 ± 0.0035	2.4130 ± 0.0490
	Peel	0.0157 ± 0.0023	0.0302 ± 0.0070	0.9822 ± 0.0130
SL2	Pulp	0.0184 ± 0.0026	0.0370 ± 0.0052	1.2810 ± 0.0772
	Peel	0.0046 ± 0.0003	0.0090 ± 0.0021	0.4652 ± 0.0084

**Table 5:** Compounds identified and quantified in the ^1^H NMR spectrum of the Bligh-Dyer hydroalcoholic extracts of BL1 Goji leaves. In brackets, the value obtained in the correspondent berries sample for each metabolite was also reported for comparison. Results were expressed as mg/100 g of fresh sample ± SD.

Metabolite	mg/100 g	Metabolite	mg/100 g	Metabolite	mg/100 g
Valine	3.98 ± 0.40 (4.5)	Citric acid	52.67 ± 4.82 (139.9)	Glucose	356.66 ± 4.20 (4829.9)
Isoleucine	2.57 ± 0.31 (4.5)	Aspartate	27.38 ± 1.84 (39.4)	Sucrose	430.13 ± 4.32 (7.3)
Leucine	6.33 ± 0.56 (6.8)	Asparagine	5.57 ± 0.36 (103.9)	Tyrosine	4.49 ± 0.54 (9.0)
Threonine	5.30 ± 0.47 (9.8)	Choline	10.44 ± 1.40 (11.4)	Phenylalanine	3.81 ± 0.57 (8.0)
Alanine	3.84 ± 0.43 (54.7)	Glycinbetaine	110.09 ± 8.23 (194)	Formic acid	1.57 ± 0.09 (0.7)
Quinic acid	82.32 ± 2.31 (60.4)	Fructose	67.42 ± 5.42 (1025.5)	Trigonelline	7.60 ± 0.82 (1.8)
GABA	24.87 ± 3.78 (5.0)	Malic acid	306.02 ± 25.48 (161.7)		

**Table 6:** Quantitative NMR results for some metabolites present in the Bligh-Dyer organic extracts of BL1 Goji leaves. Results were expressed as molar % ± SD.

Metabolite	Molar %
TOT SFA	39.57 ± 3.21
TOT UFA	57.67 ± 4.83
TUFA	48.60 ± 4.65
DUFA	9.07 ± 1.03
MUFA	/
DGDG	17.70 ± 1.70
PC	5.02 ± 0.65
β-Sitosterol	6.80 ± 0.70

## Data Availability

Not applicable.

## Supplementary Materials

The following are available online, Figure S1. Enlargement of ESI(+) FT-ICR MS Goji berries samples: BL1 (red profile), and BL2 (purple profile). Figure S2. Enlargement of ESI(+) FT-ICR MS Goji berries samples: SL1 (red profile), and SL2 (purple profile). Figure S3. Comparison between experimental ESI(–) CID assay performed by using LTQ XL ion trap and reference data obtained from Metlin database has allowed us to confirm the assignments of the following peaks at A) *m/z* 283 (stearic acid; B) *m/z* 309 (eicosenoic acid); C) *m/z* 311 (eicosanoic acid); D) *m/z* 325 (eicosanoic acid, methyl ester); E) *m/z* 337 (docosenoic acid); F) *m/z* 339 (docosanoic acid). Figure S4. Van Krevelen diagram obtained by ESI MS analysis of hydroalcoholic and organic extracts from BL and SL fruits and BL leaves. The diagrams display broken lines with the following meaning: (A) lines related to (de)hydrogenation paths; (B) lines associated with oxidation or reduction reactions; (C) lines that describe hydration and condensation processes; (D) lines that correspond to (de)methylation paths. Figure S5. Differentiation between isobaric species by ESI FT-ICR MS: histidine (*m/z* 156.07654) and valine (*m/z* 156.04220) are discriminated in BL1 (purple profile). Otherwise, histidine appears lacking in SL2 (red profile). Figure S6. Cell viability was assessed by the MTS colorimetric method. MG63 cells were treated with different concentrations, 100 μg/mL, 50 μg/mL, 25 μg/mL, 12.5 μg/mL, and 6.5 μg/mL, of Lycium barbarum Hydro-alcoholic extract (LBEHY) dissolved in deionized water and Lycium barbarum organic extract (LBEORG) dissolved in DMSO, for 24, 48, and 72 h. Cell viability of treated samples was normalized to the untreated cells, which is reported as 100% and represented by a horizontal line. Figure S7. Effects of LBEHY and LBEORG extracts on IL-6, IL-8, IL-1β, iNOS, and COX-2 mRNA expression level in MG63 cells. After 2 h-treatment with 0.1 mg/mL of 50 and 12.5 μg/mL Lycium barbarum Hydro-alcoholic extract (LBEHY) dissolved in deionized water and Lycium barbarum organic extract (LBEORG) dissolved in DMSO, cells were stimulated for 30 min with 10 ng/mL of TNFα. Cells were harvested and mRNA was extracted and analyzed by RT-PCR. IL-6, IL-8, IL-1β, iNOS, and COX-2 mRNA levels were reported as relative mRNA expression levels with respect to 18S mRNA (2-ΔΔCt method). Results are expressed as mean ± S.E.M. of data obtained by three different experiments. * *p* < 0.05; # *p* < 0.05; * represents the comparison between TNFα and LBE-treated samples; # represents the comparison between untreated (CTL) and TNFα-stimulated samples. Figure S8. A measure of IL-6 and IL-8 secretion in cell culture medium. Cells were left untreated or treated with 10 ng/mL of TNFα in the absence or in presence of 50 and 12.5 μg/mL Lycium barbarum Hydro-alcoholic extract (LBEHY) dissolved in deionized water and Lycium barbarum organic extract (LBEORG) dissolved in DMSO, for 5 h and then analyzed for IL-6 and IL-8 by ELISA method. Results (in pg/mL) are expressed as mean ± standard error, obtained in three different experiments. * *p* < 0.05; *** *p* < 0.005; ## *p* < 0.01; ### *p* < 0.005. * represents the comparison between TNFα and LBE-treated samples; # represents the comparison between untreated (CTL) and TNFα-stimulated samples. Figure S9. Effects of LBEHY and LBEORG extracts on COX-2 protein production. Upper panel: Cells were left untreated or treated with 10 ng/mL of TNFα in the absence or in presence of 50 and 12.5 μg/mL Lycium barbarum Hydro-alcoholic extract (LBEHY) dissolved in deionized water and Lycium barbarum organic extract (LBEORG) dissolved in DMSO, for 5 h and then analyzed by immunofluorescence using anti-COX-2 primary antibody and Alexa Fluor 568 (red) secondary antibody. Nuclei were stained with DAPI (original magnification 40×), images are representative of two different experiments. Lower panel: The pixel intensities, in the region of interest, were obtained by ImageJ. * *p* < 0.05; # *p* < 0.05; * represents the comparison between TNFα and LBE-treated samples; # represents the comparison between untreated (CTL) and TNFα-stimulated samples. Figure S10. Enlargement of ESI(+) FT-ICR mass spectrum of hydroalcoholic extract of Big Lifeberry leaves. Figure S8. Enlargement of ESI(+) Esquire 6000 mass spectrum of organic extract of Big Life-berry Goji leaves showing protonated pheophytin, ([C55H74N4O5 + H]+, monoisotopic peak at *m/z* 871.4). The theoretical isotopic pattern is shown in the excerpt. Table S1: Untargeted metabolic profiling of Goji Berries hydroalcoholic extracts by ESI(+) FT-ICR MS. Table S2: Untargeted metabolic profiling of Goji Berries hydroalcoholic extracts by ESI(–) FT-ICR MS. Table S3: Untargeted metabolic profiling of Goji Berries organic extracts by ESI(+) Ion Trap MS. Table S4: Untargeted metabolic profiling of Goji Berries organic extracts by ESI(–) Ion Trap MS. Table S5: Untargeted metabolic profiling of Goji Berries leaves hydroalcoholic extracts by ESI(+) FT-ICR MS. Table S6: Untargeted metabolic profiling of Goji Berries leaves hydroalcoholic extracts by ESI(-) FT-ICR MS. Table S7: Untargeted metabolic profiling of Goji Berries leaves organic extracts by ESI(+) Ion Trap MS. Table S8: Untargeted metabolic profiling of Goji Berries leaves organic extracts by ESI(–) Ion Trap MS.

## Sample Availability

Samples of the compounds are available from the authors.
