# Supplementary material for: Metabolomic Profiling of Fresh Goji (Lycium barbarum L.) Berries from Two Cultivars Grown in Central Italy: A Multi-Methodological Approach
Source: Molecules. 2021 Sep 6;26(17):5412. doi: 10.3390/molecules26175412 (PMC8433735; doi:10.3390/molecules26175412)
Supplement: Supplementary file 1 [file molecules-26-05412-s001.zip › molecules-1349754-supplementary.v5.pdf]

molecules

MDPI

Supporting Information

Metabolomic profiling of fresh Goji (*Lycium barbarum* L.) berries from two cultivars grown in Center Italy: a multi-methodological approach

Mattia Spano[1,†], Alessandro Maccelli[1,†], Giacomo Di Matteo[1], Cinzia Ingallina[1], Mariangela Biava[1], Maria Elisa Crestoni[1,*], Jean-Xavier Bardaud[2], Anna Maria Giusti[3], Alessia Mariano[4], Anna Scotto D'Abusco[4], Anatoly P. Sobolev[5,*], Alba Lasalvia [1], Simonetta Fornarini[1], Luisa Mannina[1]

[1] Department of Chemistry and Technology of Drugs, Sapienza University of Rome, Piazzale Aldo Moro 5, Rome 00185, Italy; mattia.spano@uniroma1.it (M.S.); alessandro.maccelli@uniroma1.it (A.M.); giacomo.dimatteo@uniroma1.it (G.D.M.); cinzia.ingallina@uniroma1.it (C.I.); mariangela.biava@uniroma1.it (M.B.); alba.lasalvia@uniroma1.it (A.L.); simonetta.fornarini@uniroma1.it (S.F.); luisa.mannina@uniroma1.it (L.M.)

[2] Institut de Chimie Physique, CLIO, Université Paris Saclay, Bât 200, BP34, Orsay Cedex 91898, France; j.xavier.bardaud@gmail.com

[3] Department of Experimental Medicine, Sapienza University of Rome, P.le Aldo Moro 5, Rome 00185, Italy; annamaria.giusti@uniroma1.it

[4] Department of Biochemical Sciences, Sapienza University of Roma, P.le Aldo Moro 5, Rome 00185, Italy; alessia.mariano@uniroma1.it (A.M.); anna.scottodabusco@uniroma1.it (A.S.D.)

[5] Institute for Biological Systems, Magnetic Resonance Laboratory "Segre-Capitani", CNR, Via Salaria Km 29.300, Monterotondo 00015, Italy

† These authors contributed equally to this work

* Correspondence: mariaelisa.crestoni@uniroma1.it (M.E.C.), anatoly.sobolev@cnr.it (A.P.S.)

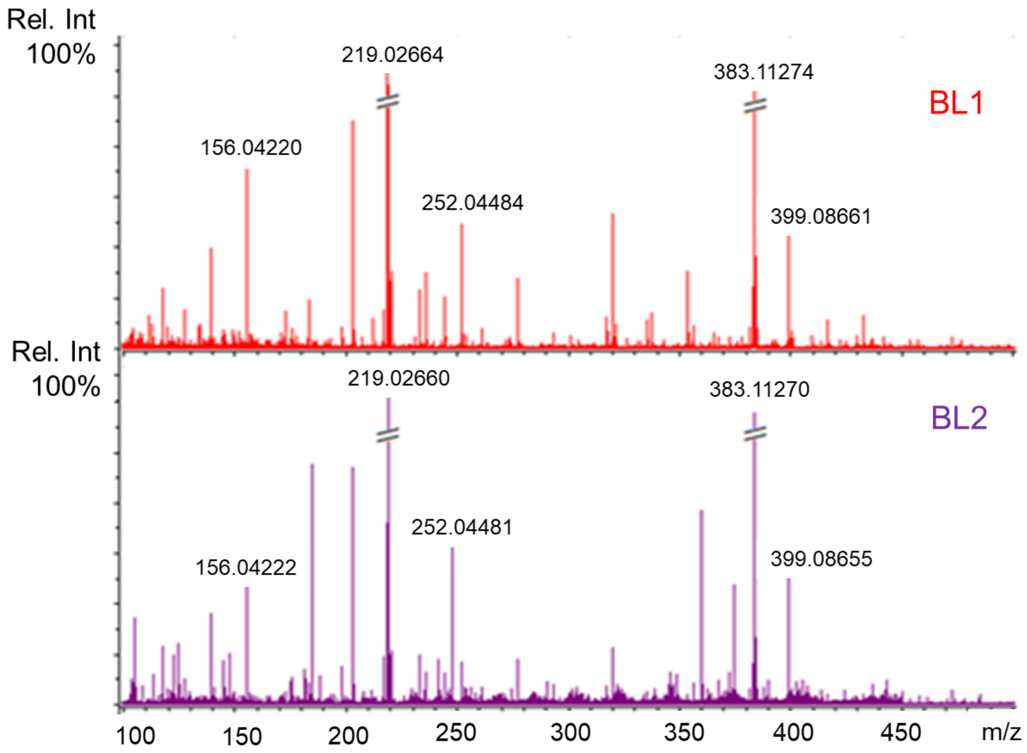

Figure S1. Enlargement of ESI(+) FT-ICR mass spectra Goji berries samples: BL1 (red profile), and BL2 (purple profile).

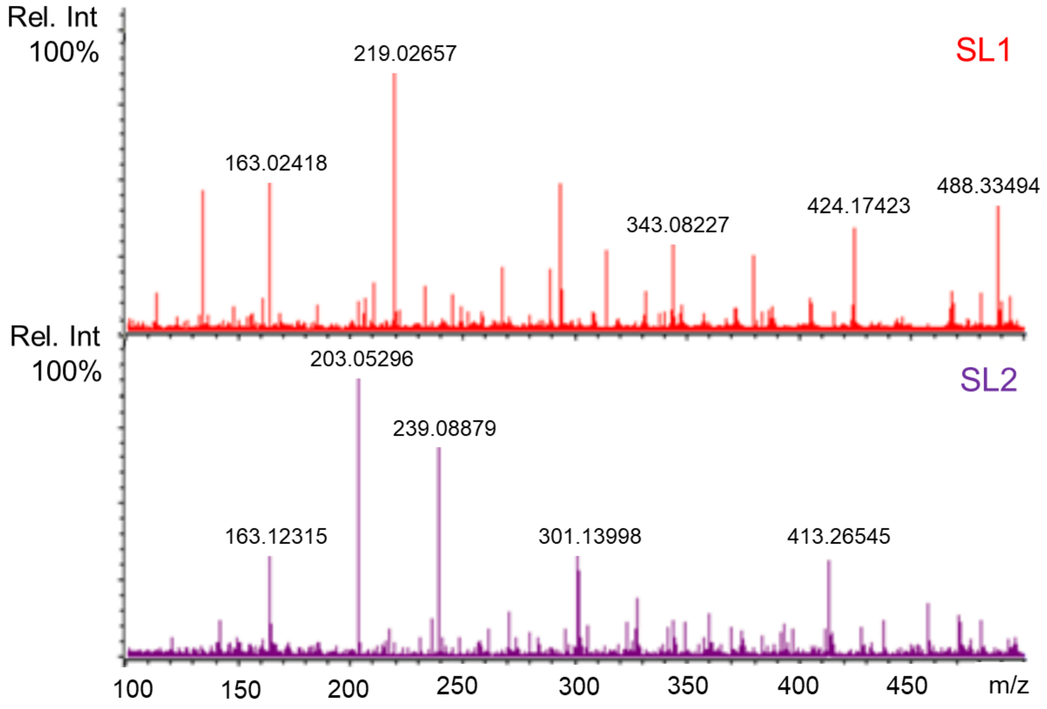

Figure S2. Enlargement of ESI(+) FT-ICR mass spectra Goji berries samples: SL1 (red profile), and SL2 (purple profile).

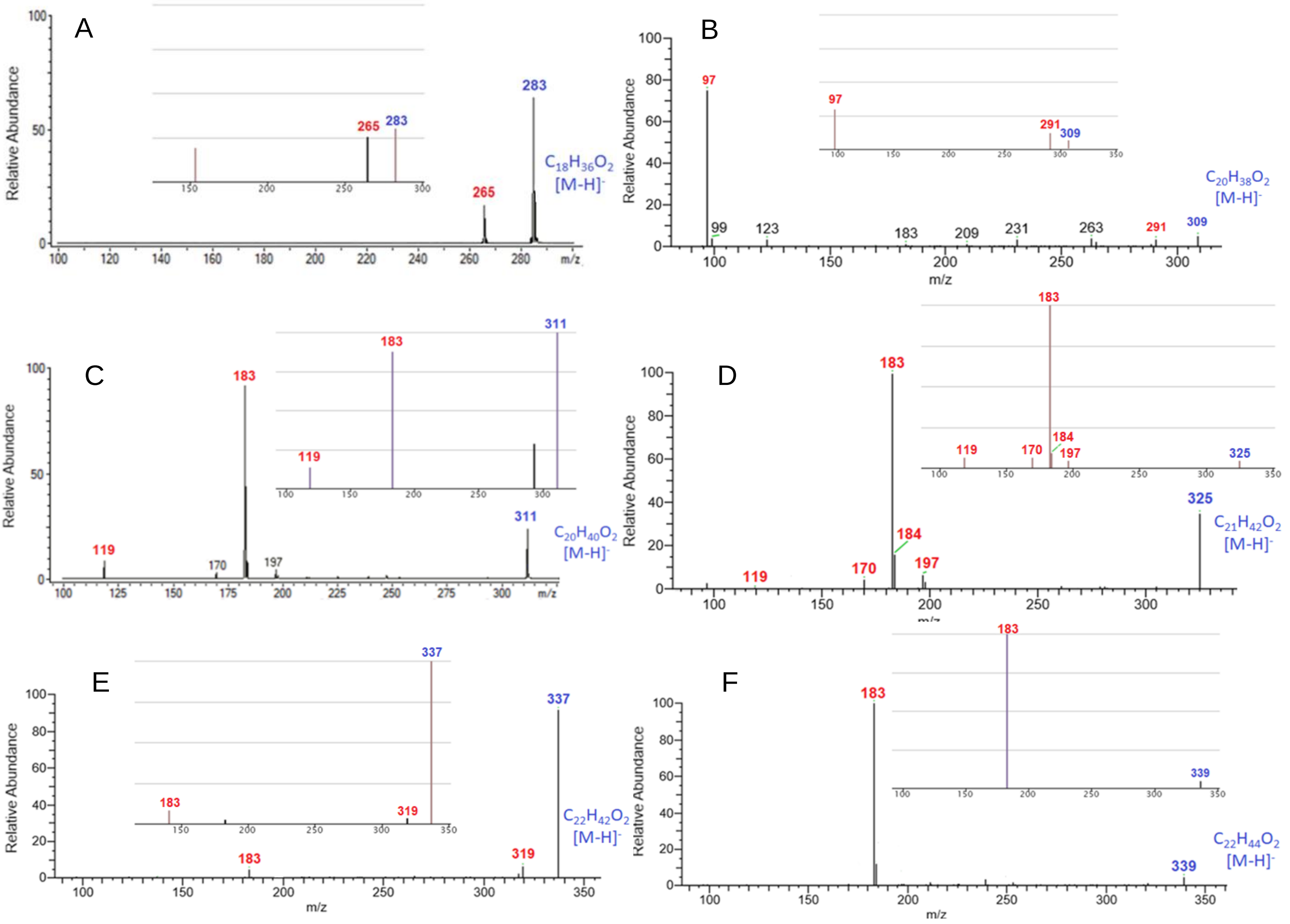

Figure S3. Comparison between experimental ESI(-) CID assay performed with LTQ XL ion trap and reference data obtained from Metlin database has allowed to confirm the assignments of the following peaks at : A) m/z 283 (stearic acid; B) m/z 309 (eicosenoic acid); C) m/z 311 (eicosanoic acid); D) m/z 325 (eicosanoic acid, methyl ester); E) m/z 337 (docosenoic acid); F) m/z 339 (docosanoic acid).

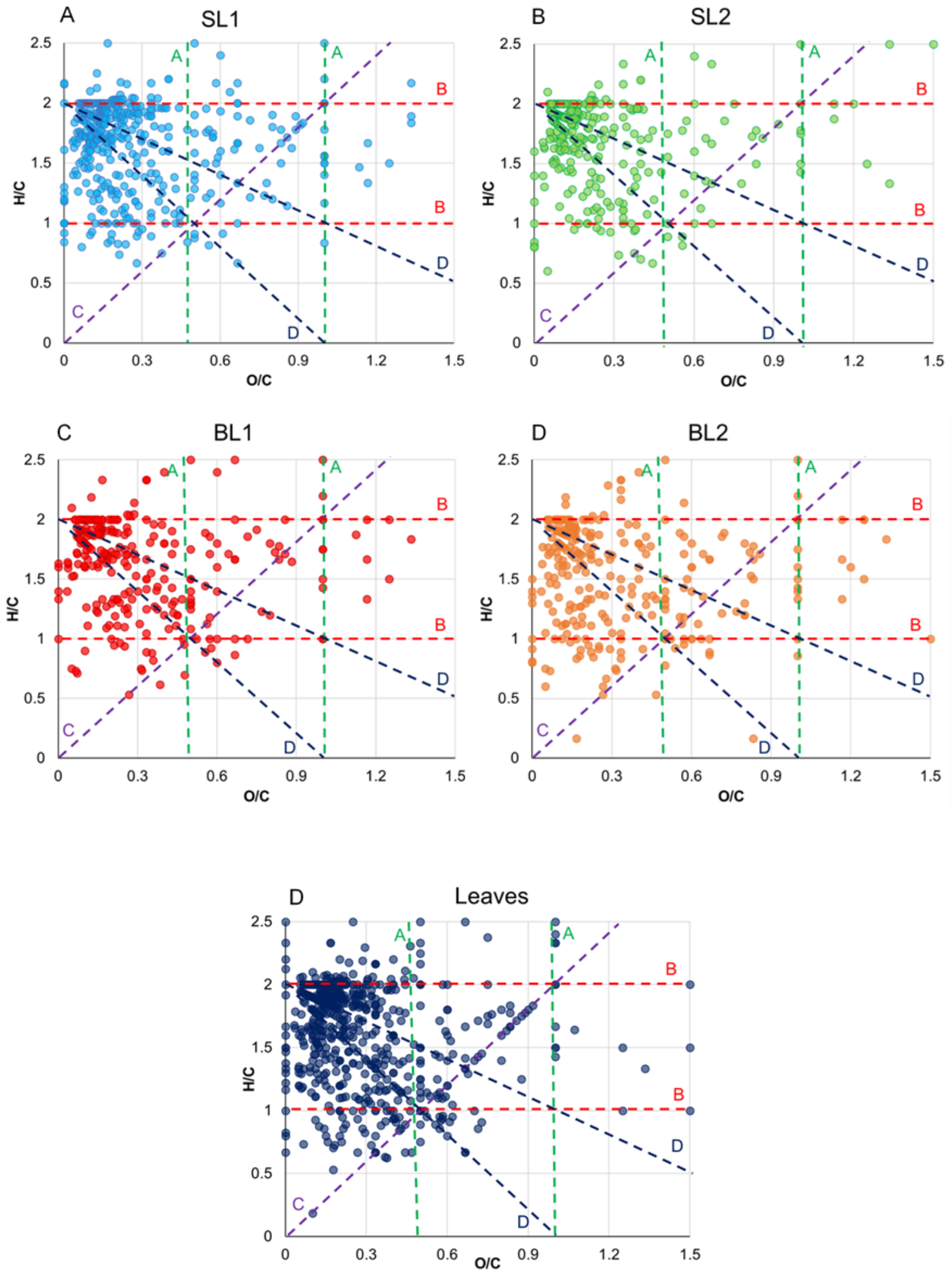

Figure S4. Van Krevelen diagram obtained by ESI MS analysis of hydroalcoholic and organic extracts from BL and SL fruits and BL leaves. The diagrams display broken lines with the following meaning: (A) lines related to (de)hydrogenation paths; (B) lines associated to oxidation or reduction reactions; (C) lines that describe hydration and condensation processes; (D) lines that correspond to (de)methylation paths.

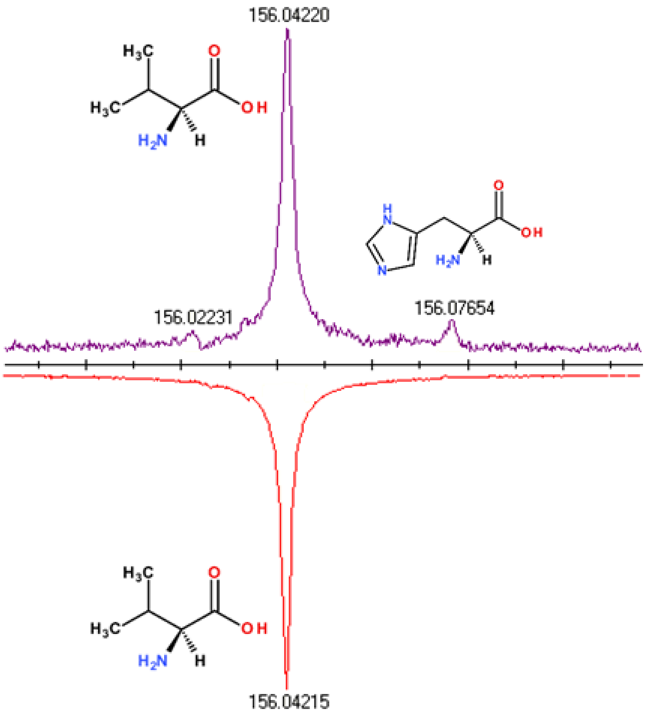

Figure S5. Differentiation between isobaric species by ESI(+) FT-ICR MS: histidine (m/z 156.07654) and valine (m/z 156.04220) are discriminated in BL1 (purple profile). Otherwise, histidine appears lacking in SL2 (red profile).

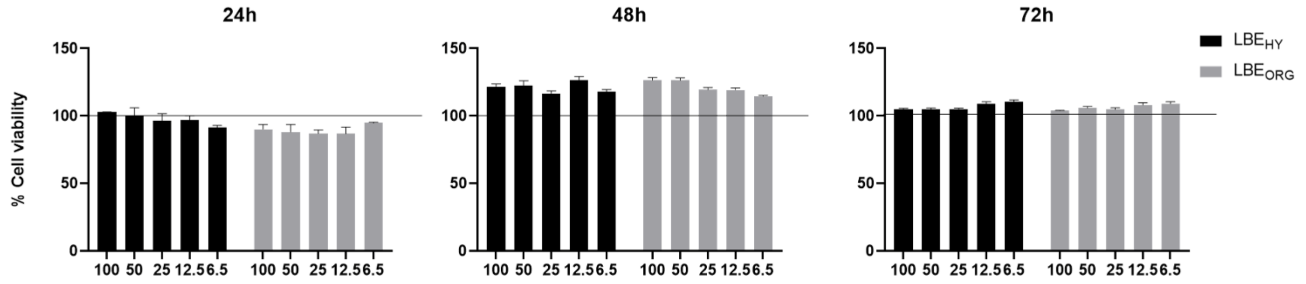

Figure S6. Cell viability was assessed by the MTS colorimetric method. MG63 cells were treated with different concentrations, 100 µg/mL, 50 µg/mL, 25 µg/mL, 12.5 µg/mL and 6.5 µg/mL, of Lycium barbarum Hydro-alcoholic extract (LBEHY) dissolved in deionized water and Lycium barbarum organic extract (LBEORG) dissolved in DMSO, for 24, 48, and 72 h. Cell viability of treated samples was normalized to the untreated cells, which is reported as 100% and represented by a horizontal line.

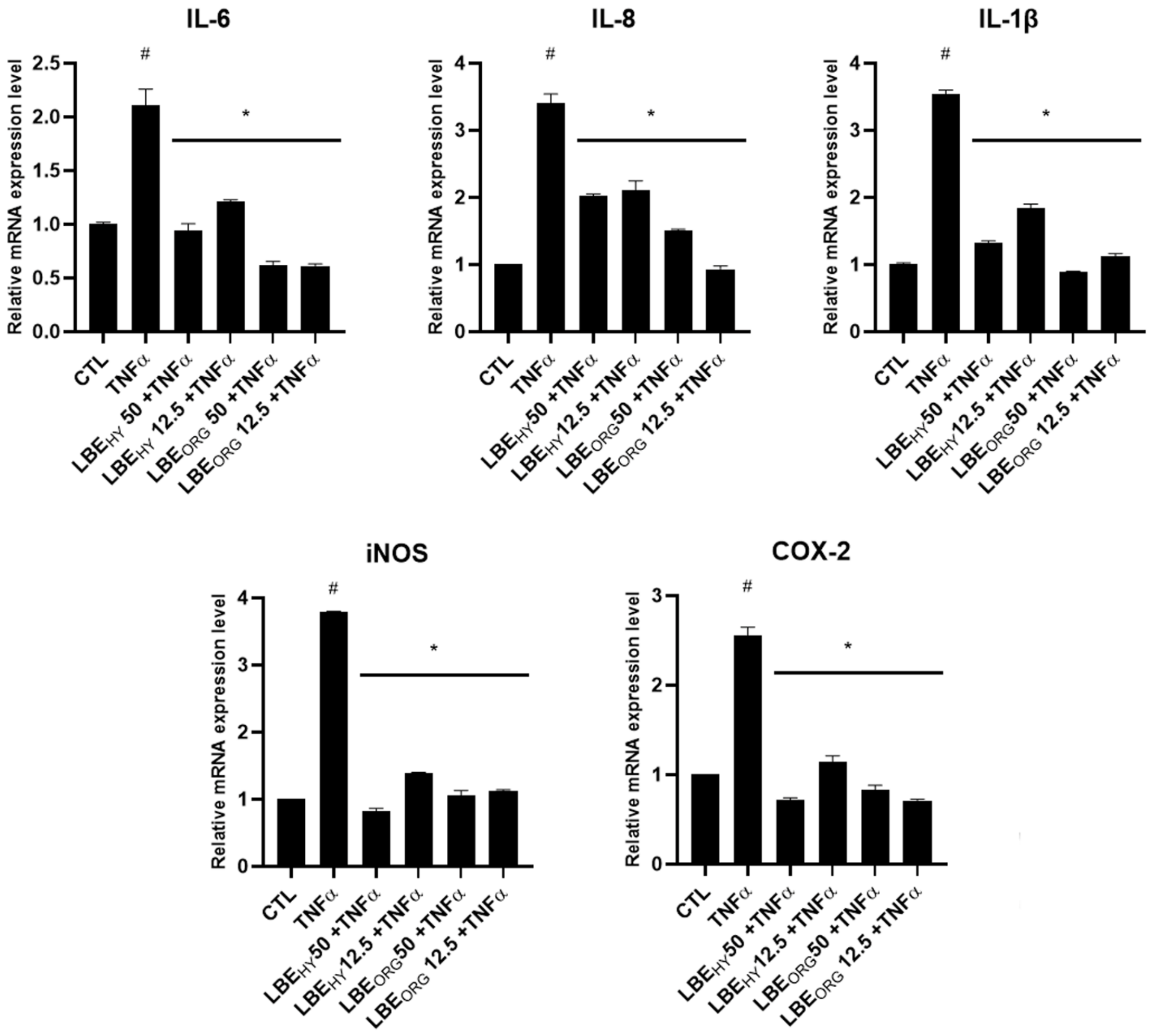

Figure S7. Effects of LBEHY and LBEORG extracts on IL-6, IL-8, IL-1β, iNOS and COX-2 mRNA expression level in MG63 cells. After 2 hour-treatment with 0.1 mg/mL of 50 and 12.5 µg/mL Lycium barbarum Hydro-alcoholic extract (LBEHY) dissolved in deionized water and Lycium barbarum organic extract (LBEORG) dissolved in DMSO, cells were stimulated for 30 min with 10 ng/mL of TNFα. Cells were harvested and mRNA was extracted and analyzed by RT-PCR. IL-6, IL-8, IL-1β, iNOS and COX-2

mRNA levels were reported as relative mRNA expression level with respect to 18S mRNA (2-ΔΔCt method). Results are expressed as mean ± S.E.M. of data obtained by three different experiments. *$p < 0.05$; #$p < 0.05$; * represents the comparison between TNFα and LBE-treated samples; # represents the comparison between untreated (CTL) and TNFα-stimulated samples.

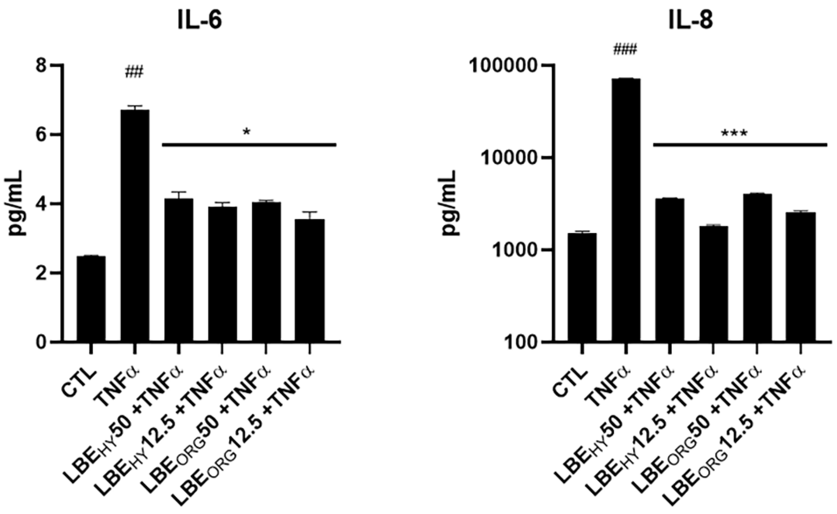

Figure S8. Measure of IL-6 and IL-8 secretion in cell culture medium. Cells were left untreated or treated with 10 ng/mL of TNFα in absence or in presence of 50 and 12.5 μg/mL Lycium barbarum Hydro-alcoholic extract (LBEHY) dissolved in deionized water and Lycium barbarum organic extract (LBEORG) dissolved in DMSO, for 5 h and then analysed for IL-6 and IL-8 by ELISA method. Results (in pg/mL) are expressed as mean ± standard error, obtained in three different experiments. *$p < 0.05$; ***p< 0.005; ##p< 0.01; ###p<0.005. * represents the comparison between TNFα and LBE-treated samples; # represents the comparison between untreated (CTL) and TNFα-stimulated samples.

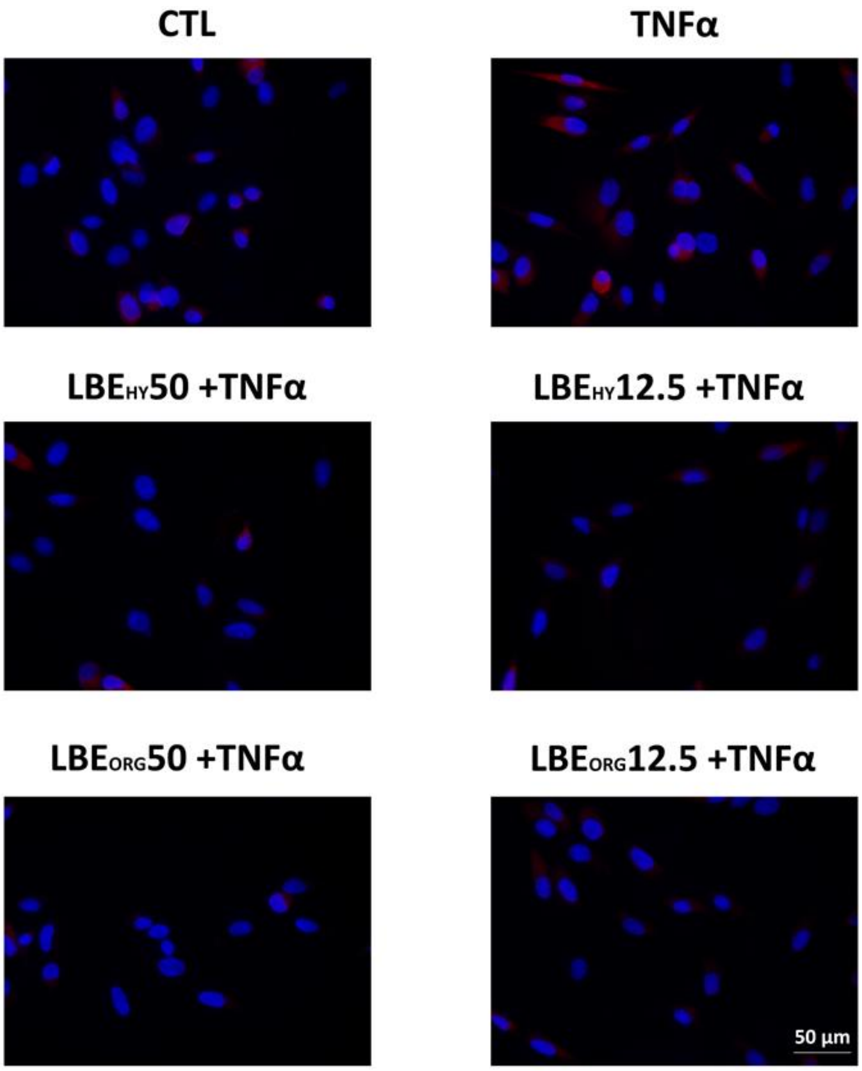
CTL
TNFα
LBEHY50 +TNFα
LBEHY12.5 +TNFα
LBEORG50 +TNFα
LBEORG12.5 +TNFα
50 µm

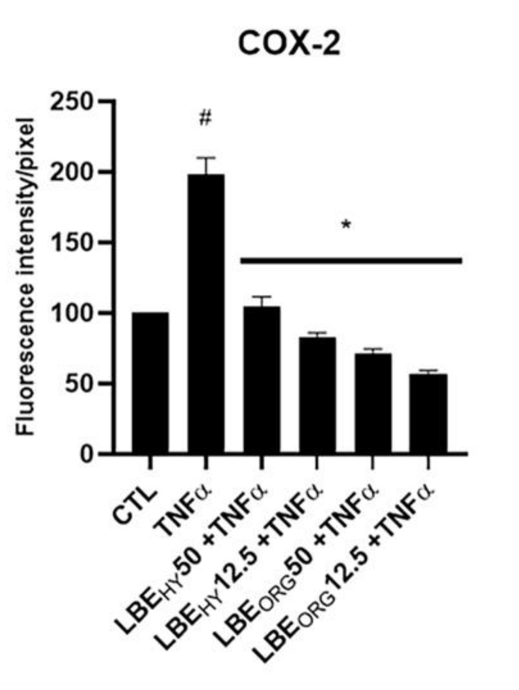
COX-2
Fluorescence intensity/pixel
250
200
150
100
50
0
#
*
CTL
TNFα
LBEHY50 +TNFα
LBEHY12.5 +TNFα
LBEORG50 +TNFα
LBEORG12.5 +TNFα

Figure S9. Effects of LBEHY and LBEORG extracts on COX-2 protein production. Upper panel: Cells were left untreated or treated with 10 ng/mL of TNFα in absence or in presence of 50 and 12.5 µg/mL Lycium barbarum Hydro-alcoholic extract (LBEHY) dissolved in deionized water and Lycium barbarum organic extract (LBEORG) dissolved in DMSO, for 5 h and then analyzed by immunofluorescence using anti-COX-2 primary antibody and Alexa Fluor 568 (red) secondary antibody. Nuclei were stained with DAPI (original magnification 40×), images are representative of two different experiments. Lower panel: The pixel intensities, in the region of interest, were obtained by ImageJ. *$p < 0.05$; #$p < 0.05$; * represents the comparison between TNFα and LBE-treated samples; # represents the comparison between untreated (CTL) and TNFα-stimulated samples.

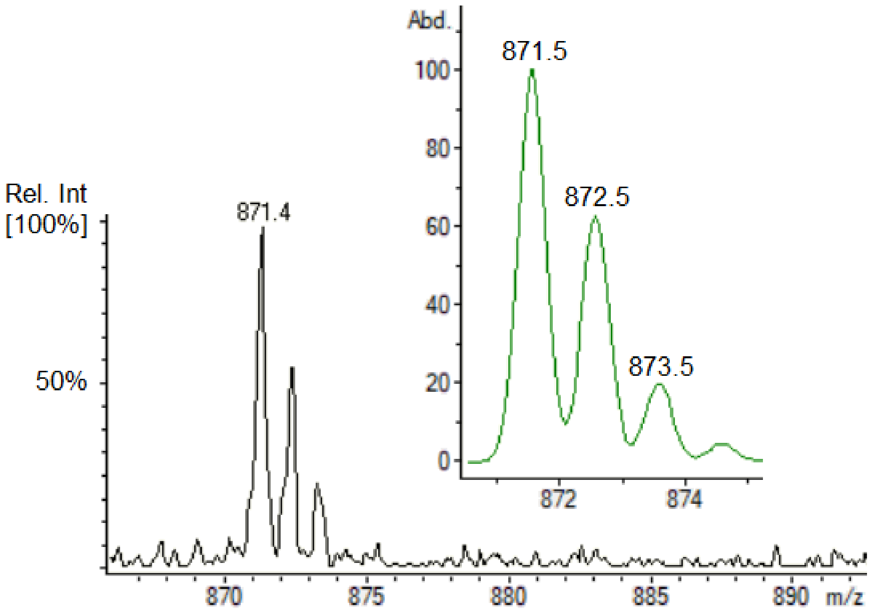

Figure S10. Enlargement of ESI(+) Esquire 6000 mass spectrum of organic extract of Big Lifeberry leaves showing protonated pheophytin a, ($[C_{55}H_{74}N_4O_5 + H]^+$, monoisotopic peak at m/z 871.4). The theoretical isotopic pattern is shown in the excerpt.

Table S1: Untargeted metabolic profiling of Goji Berries hydroalcoholic extracts by ESI(+) FT-R MS

#	Putative annotation[a]	Ion	Formula (M)	m/z$_{Theo}$[b]	BL1[c]	ppm	BL2[c]	ppm	SL1[c]	ppm	SL2[c]	ppm
1	4-Aminopyridine	[M+H]+	C5H6N2	95.06037					1.1E+06	-0.8		
2	Guanidine	[M+K]+	CH5N3	98.01151					4.8E+05	-1.6		
3	Glycine	[M+Na]+	C2H5NO2	98.02125							1.2E+05	2.3
4	Ethanolamine	[M+K]+	C2H7NO	100.01592					4.4E+05	-2.1		
5	Allyl isothiocyanate	[M+H]+	C4H5NS	100.02155					9.2E+05	-0.2		
6	Cadaverine	[M+H]+	C5H14N2	103.12297							1.4E+05	0.3
7	Choline	[M]+	C5H14NO	104.10699	5.3E+05	1.2	1.5E+06	2.3	9.9E+05	-2.3	1.6E+05	0.6
8	S-Methylthioglycolate	[M+H]+	C3H6O2S	107.01613					8.4E+05	-1.1		
9	Iminoglycine	[M+K]+	C2H3NO2	111.97954					5.2E+05	1.4		
10	Alanine	[M+Na]+	C3H7NO2	112.03690							2.1E+05	1.7
11	Glycerol	[M+Na]+	C3H8O3	115.03656							1.1E+05	0.8
12	3-Hydroxyhexanal	[M+H]+	C6H12O2	117.09101	3.9E+05	-2.1			6.8E+05	1.1		
13	L-Valine	[M+H]+	C5H11NO2	118.08626	2.8E+06	-1.4	5.2E+06	0.5	4.3E+05	1.9	2.4E+05	0.2
14	1-Hexen-1-ol	[M+Na]+	C6H12O	123.07804			5.0E+05	2.9	4.3E+05	2.9		
15	Malonic acid	[M+Na]+	C3H4O4	127.00018							1.1E+05	1.0
16	L-Lactic acid	[M+K]+	C3H6O3	128.99485	5.2E+05	0.9						
17	3-Aminopropane-1,2-diol	[M+K]+	C3H9NO2	130.02649			4.4E+05	2.2				
18	D-Ornithine	[M+H]+	C5H12N2O2	133.09715	4.5E+05	0.9	4.5E+05	1.2				
19	2-Hydroxypyridine	[M+K]+	C5H5NO	134.00027			5.0E+05	-2.3			2.2E+05	1.6
20	D-Proline	[M+Na]+	C5H9NO2	138.05255	3.5E+05	0.3	3.5E+05	2.8	1.9E+06	-0.8		
21	Tyramine	[M+H]+	C8H11NO	138.09134					4.9E+05	-2.2		
22	Butyl acetate	[M+Na]+	C6H12O2	139.07295					4.5E+05	-2.4		
23	4-Hydroxyphenylethanol	[M+H]+	C8H10O2	139.07536					4.5E+05	-2.4		
24	L-Valine	[M+Na]+	C5H11NO2	140.06820	4.3E+06	-0.9	8.3E+06	0.0	4.8E+05	0.6		
25	2,4-Diaminobutyric acid	[M+Na]+	C4H10N2O2	141.06345	4.4E+05	-1.7	1.0E+06	2.3	5.4E+05	1.8		
26	L-Lysine	[M+H]+	C6H14N2O2	147.11280	3.8E+05	1.0	5.9E+05	1.0				

Table S1: Untargeted metabolic profiling of Goji Berries hydroalcoholic extracts by ESI(+) FT-R MS

#	Putative annotation[a]	Ion	Formula (M)	m/z$_{Theo}$[b]	BL1[c]	ppm	BL2[c]	ppm	SL1[c]	ppm	SL2[c]	ppm
27	2-Hydroxyethylphosphonate	[M+Na]+	C2H7O4P	148.99742	3.8E+05	-0.7			2.2E+06	2.3		
28	L-Methionine	[M+H]+	C5H11NO2S	150.05833					1.2E+06	-2.2		
29	Pyroglutamic acid	[M+Na]+	C5H7NO3	152.03181	3.7E+05	2.7	3.7E+05	-1.7				
30	epsilon-Caprolactam	[M+K]+	C6H11NO	152.04722			4.6E+05	2.7				
31	2Z-Hexenoic acid	[M+K]+	C6H10O2	153.03124			4.0E+05	2.9				
32	1-Octanol	[M+Na]+	C8H18O	153.12499					1.6E+06	-2.1		
33	L-Proline	[M+K]+	C5H9NO2	154.02649	4.2E+05	-0.6	4.4E+05	0.3				
34	L-Valine	[M+K]+	C5H11NO2	156.04214	8.5E+06	-0.8	1.1E+07	0.3	2.4E+06	0.0	1.0E+05	-2.1
35	L-Histidine	[M+H]+	C6H9N3O2	156.07675	1.2E+06	2.9	1.2E+06	-1.4	1.6E+05	-1.7		
36	Cinnamyl alcohol	[M+Na]+	C9H10O	157.06239	9.6E+05	-0.3	9.6E+05	2.1				
37	2-Isopropylmaleate	[M+H]+	C7H10O4	159.06519			5.7E+05	1.2				
38	Valerylglycine	[M+H]+	C7H13NO3	160.09682			5.1E+05	-2.2				
39	Allicin	[M+H]+	C6H10OS2	163.02458					1.5E+06	-2.5		
40	(R)-Nicotine	[M+H]+	C10H14N2	163.12297	3.5E+05	2.1	5.4E+05	0.8			2.6E+05	1.1
41	2-O-Methylcytosine	[M+K]+	C5H7N3O	164.02207					2.6E+06	3.0		
42	Thymine	[M+K]+	C5H6N2O2	165.00609	7.6E+05	2.6	7.6E+05	1.9				
43	(E)-p-coumaric acid	[M+H]+	C9H8O3	165.05462					6.8E+05	0.1		
44	2,3,6-Trihydroxypyridine	[M+K]+	C5H5NO3	165.99010	4.9E+05	1.4	4.9E+05	0.8			3.2E+05	-2.6
45	(-)-Hygroline	[M+Na]+	C8H17NO	166.12023			7.0E+05	2.4	7.0E+05	2.7		
46	Dihydrothymine	[M+K]+	C5H8N2O2	167.02174					2.1E+06	-0.4		
47	2-methyl-hexanoic acid	[M+K]+	C7H14O2	169.06254							1.2E+05	-0.5
48	Leucine	[M+K]+	C6H13NO2	170.05779			4.8E+05	1.8	1.4E+06	-2.6	1.3E+05	-2.0
49	Lupinine	[M+H]+	C10H19NO	170.15394			5.0E+05	3.0	1.8E+06	2.1		
50	Asparagine	[M+K]+	C4H8N2O3	171.01665	7.3E+05	-0.9	7.6E+05	0.2				
51	Hydrocinnamic acid	[M+Na]+	C9H10O2	173.05730	1.4E+06	1.0	1.2E+06	1.7	1.0E+06	0.4		
52	L-Arginine	[M+H]+	C6H14N4O2	175.11895	3.8E+05	1.0	2.0E+06	0.3			2.2E+05	0.7

Table S1: Untargeted metabolic profiling of Goji Berries hydroalcoholic extracts by ESI(+) FT-R MS

#	Putative annotation[a]	Ion	Formula (M)	m/z_{Theo}[b]	BL1[c]	ppm	BL2[c]	ppm	SL1[c]	ppm	SL2[c]	ppm
53	Monodehydroascorbate	[M+H]+	C6H7O6	176.03154	6.9E+05	-1.5	5.1E+05	-0.2	6.1E+05	-0.2		
54	N-Carboxyethyl-g-aminobutyric acid	[M+H]+	C7H13NO4	176.09173	8.8E+05	-0.5	2.5E+06	-0.1			5.0E+05	2.9
55	Tecomine	[M+H]+	C11H17NO	180.13829					1.2E+06	-0.7		
56	L-Dihydroorotic acid	[M+Na]+	C5H6N2O4	181.02198	3.7E+05	0.0	3.7E+05	0.1				
57	4-Hydroxy-L-glutamic acid	[M+Na]+	C5H7NO5	184.02164					9.9E+05	-0.5		
58	L-Lysinamide	[M+K]+	C6H15N3O	184.08467					1.1E+06	-2.1		
59	D-Glutamine	[M+K]+	C5H10N2O3	185.03230							9.7E+04	-0.7
60	Tridecane	[M+H]+	C13H28	185.22638					1.1E+06	-0.7		
61	L-Methionine	[M+K]+	C5H11NO2S	188.01421							8.3E+04	0.9
62	D-Ribose	[M+K]+	C5H10O5	189.01598			5.5E+05	-2.5				
63	Caffeyl alcohol	[M+Na]+	C9H10O3	189.05221							2.4E+05	2.3
64	3-Isopropylcatechol	[M+K]+	C9H12O2	191.04689	4.2E+05	-2.9						
65	Thiobenzamide S-oxide	[M+K]+	C7H7NOS	191.98799					3.0E+06	2.3		
66	3-Hydroxyanthranilic acid	[M+K]+	C7H7NO3	192.00575					7.5E+05	-2.5		
67	2-Amino-3,7-dideoxy-D-threo-hept-6-ulosonic acid	[M+H]+	C7H13NO5	192.08665	4.1E+05	0.6	4.2E+05	0.5			2.0E+05	0.3
68	3-Oxooctanoic acid	[M+K]+	C8H14O3	197.05745							1.5E+05	1.5
69	5-Acetylamino-6-amino-3-methyluracil	[M+H]+	C7H10N4O3	199.08257							1.0E+05	2.9
70	Methyl cinnamate	[M+K]+	C10H10O2	201.03124							9.3E+04	1.8
71	1-Phenylpropyl acetate	[M+Na]+	C11H14O2	201.08860	5.7E+05	2.4	5.7E+05	-1.0				
72	4-Aminophenyl ether	[M+H]+	C12H12N2O	201.10224			1.4E+06	-1.5				
73	(E)-4-Oxobut-1-ene-1,2,4-tricarboxylate	[M+H]+	C7H6O7	203.01863			7.8E+05	0.5				
74	Aldohexose	[M+Na]+	C6H12O6	203.05261	1.2E+07	-0.2	2.8E+07	0.3	4.0E+06	0.0	1.4E+07	1.7
75	Succimer	[M+Na]+	C4H6O4S2	204.95997					4.6E+06	1.3		
76	Dimethylenetriurea	[M+H]+	C5H12N6O3	205.10436					1.1E+06	2.6	1.0E+05	1.6
77	Caulophylline	[M+H]+	C12H16N2O	205.13354					6.5E+06	2.6		
78	N-Acetyl-D-phenylalanine	[M+H]+	C11H13NO3	208.09682			4.0E+06	2.9	9.3E+05	2.0		

Table S1: Untargeted metabolic profiling of Goji Berries hydroalcoholic extracts by ESI(+) FT-R MS

#	Putative annotation[a]	Ion	Formula (M)	m/z_{Theo}[b]	BL1[c]	ppm	BL2[c]	ppm	SL1[c]	ppm	SL2[c]	ppm
79	(S)-dihydrolipoic acid	[M+H]+	C8H16O2S2	209.06645					1.0E+06	-2.3	1.3E+05	1.3
80	8-Amino-7-oxononanoate	[M+Na]+	C9H17NO3	210.11006			5.2E+05	1.7			4.3E+05	2.3
81	5,8,11-dodecatriynoic acid	[M+Na]+	C12H12O2	211.07295							3.7E+05	3.0
82	2E,8E-Undecadiene-4,6-diynoic acid	[M+K]+	C11H10O2	213.03124			6.3E+05	-1.5				
83	2-Hydroxy-6-ketononatrienedioate	[M+H]+	C9H8O6	213.03936			5.2E+05	-1.9				
84	(+)-3-hydroxy pelargonic acid	[M+K]+	C9H18O3	213.08875							1.3E+05	-1.8
85	N-Carboxyethyl-g-aminobutyric acid	[M+K]+	C7H13NO4	214.04762			1.8E+06	1.5				
86	Quinic acid	[M+Na]+	C7H12O6	215.05261							7.1E+04	-2.9
87	2E,4E,8Z,10E-dodecatetraenoic acid	[M+Na]+	C12H16O2	215.10425					9.5E+05	1.8		
88	(+)-10-methyl lauric acid	[M+H]+	C13H26O2	215.20056					8.6E+05	1.7		
89	Aldohexose	[M+K]+	C6H12O6	219.02655	4.7E+07	0.0	6.6E+07	0.3	3.6E+07	0.1	8.5E+05	1.9
90	4-Nitrophenyl sulfate	[M+H]+	C6H5NO6S	219.99103					9.8E+05	-2.0		
91	Tauropine	[M+Na]+	C5H11NO5S	220.02501	4.4E+06	0.2	1.3E+07	-2.3	9.6E+05	1.0		
92	1D-1-Guanidino-1-deoxy-3-dehydro-scyllo-inositol	[M+H]+	C7H13N3O5	220.09280					2.0E+06	-2.6		
93	2,4-Dinitrophenylhydrazine	[M+Na]+	C6H6N4O4	221.02813	4.9E+06	1.0	1.4E+07	-0.3	1.8E+06	0.2	9.9E+04	0.1
94	Imidazole acetol-phosphate	[M+H]+	C6H9N2O5P	221.03218			1.3E+06	-2.5	1.5E+06	-11.0	1.8E+05	2.5
95	Diisopropyl phosphate	[M+K]+	C6H15O4P	221.03396	8.8E+05	-0.1						
96	4-Amino-2-hydroxylamino-6-nitrotoluene	[M+K]+	C7H9N3O3	222.02755	4.1E+05	1.8	3.8E+05	2.4	2.3E+06	-0.5		
97	7-oxo-8-amino-nonanoic acid	[M+K]+	C9H17NO3	226.08400			8.4E+05	2.7				
98	(E)-2-(Methoxycarbonylmethyl)butenedioate	[M+K]+	C7H8O6	226.99525					9.5E+05	-2.1		
99	5,8,11-dodecatriynoic acid	[M+K]+	C12H12O2	227.04689					8.0E+05	-0.5		
100	Nonate	[M+K]+	C9H16O4	227.06802							1.0E+05	2.0
101	Myristoleic acid	[M+H]+	C14H26O2	227.20056					8.3E+05	-0.5		
102	2-Methylcitric acid	[M+Na]+	C7H10O7	229.03187	3.6E+05	0.8	7.6E+05	-1.8			8.1E+04	0.5
103	5'-Hydroxycotinine	[M+K]+	C10H12N2O2	231.05304					9.6E+05	1.8		
104	cis-1,2-Diphenylcyclobutane	[M+Na]+	C16H16	231.11442	9.9E+05	1.6	5.2E+05	-2.6				

Table S1: Untargeted metabolic profiling of Goji Berries hydroalcoholic extracts by ESI(+) FT-R MS

#	**Putative annotation**[a]	**Ion**	**Formula (M)**	**m/z$_{Theo}$**[b]	**BL1**[c]	**ppm**	**BL2**[c]	**ppm**	**SL1**[c]	**ppm**	**SL2**[c]	**ppm**
105	15-octadecene-9,11,13-triynoic acid	[M+H]+	C15H18O2	231.13796					2.8E+06	-2.2		
106	5-Hydroxyferulic acid	[M+Na]+	C10H10O5	233.04204			6.4E+05	2.6				
107	Sinapyl alcohol	[M+Na]+	C11H14O4	233.07843	2.4E+06	0.9	4.6E+06	1.1			6.1E+04	-2.1
108	3-(4'-Methylthio)butylmalic acid	[M+H]+	C9H16O5S	237.07912					1.6E+06	-1.0	3.0E+05	-1.9
109	2E-Decenedioic acid	[M+K]+	C10H16O4	239.06802			5.0E+05	1.7				
110	N-Phenyl-1-naphthylamine	[M+Na]+	C16H13N	242.09402			7.7E+05	-1.2			1.3E+05	-2.9
111	L-Tryptophan	[M+K]+	C11H12N2O2	243.05304			8.9E+05	1.4				
112	Oblongolide	[M+Na]+	C14H20O2	243.13555							1.7E+05	0.6
113	Diethylpropion	[M+K]+	C13H19NO	244.10982					8.6E+05	-0.3		
114	2-Methylcitric acid	[M+K]+	C7H10O7	245.00581	5.3E+05	-0.7	1.7E+06	-2.5				
115	Apiole	[M+Na]+	C12H14O4	245.07843							5.7E+05	2.5
116	Glycerophosphoglycerol	[M+H]+	C6H15O8P	247.05773							1.5E+05	-0.6
117	Chorismate	[M+Na]+	C10H10O6	249.03696	4.2E+05	0.4	4.9E+05	-0.5				
118	N-Caffeoylputrescine	[M+H]+	C13H18N2O3	251.13902							2.7E+05	-2.1
119	Morindaparvin A	[M+H]+	C15H8O4	253.04954	1.1E+06	-2.8	4.4E+05	-2.3				
120	Kinetin	[M+K]+	C10H9N5O	254.04387	5.3E+05	-0.8	7.2E+05	1.2	1.5E+06	1.5		
121	Tetrahydroharmine	[M+K]+	C13H16N2O	255.08942			7.1E+05	-2.6				
122	3-Hydroxydodecanoic acid	[M+K]+	C12H24O3	255.13570			6.7E+05	-2.9				
123	(S)-beta-Methylindolepyruvate	[M+K]+	C12H11NO3	256.03705			1.5E+06	-0.2				
124	O-Succinyl-L-homoserine	[M+K]+	C8H13NO6	258.03745					1.5E+06	-1.9		
125	Ngamma-Nitro-L-arginine	[M+K]+	C6H13N5O4	258.05991					1.7E+06	2.9		
126	Darlingine	[M+K]+	C13H17NO2	258.08909					1.8E+06	-0.8		
127	N-Lauroylglycine	[M+H]+	C14H27NO3	258.20637					1.5E+06	-1.3		
128	Apiole	[M+K]+	C12H14O4	261.05237							3.8E+05	1.4
129	3-Deoxy-D-manno-octulosonate	[M+Na]+	C8H14O8	261.05809	7.8E+05	0.2	1.3E+06	0.4			2.9E+05	1.1
130	3-Methoxy-4-hydroxyphenylethyleneglycol sulfate	[M+H]+	C9H12O7S	265.03765					1.0E+06	-0.9	3.0E+05	1.9

Table S1: Untargeted metabolic profiling of Goji Berries hydroalcoholic extracts by ESI(+) FT-R MS

#	Putative annotation[a]	Ion	Formula (M)	m/z_{Theo}[b]	BL1[c]	ppm	BL2[c]	ppm	SL1[c]	ppm	SL2[c]	ppm
131	Subaphyllin	[M+H]+	C14H20N2O3	265.15467			5.0E+05	2.2			1.6E+05	-2.4
132	(+)-12-methyl myristic acid	[M+Na]+	C15H30O2	265.21380							8.1E+04	-2.8
133	10-Hydroxybenzo[a]pyrene	[M+H]+	C20H12O	269.09609			9.0E+05	0.6			1.3E+05	-1.3
134	5,6-Dehydrolupanine	[M+Na]+	C15H22N2O	269.16243			9.1E+05	2.3				
135	N-Caffeoylputrescine	[M+Na]+	C13H18N2O3	273.12096	7.0E+05	-1.9	4.8E+05	-1.3				
136	N-Acetylgalactosaminate	[M+K]+	C8H13NO7	274.03236					1.2E+06	1.8		
137	Brugine	[M+H]+	C12H19NO2S2	274.09300					9.1E+05	-2.7		
138	L-Thyronine	[M+H]+	C15H15NO4	274.10738					1.4E+06	0.0		
139	Glutaconylcarnitine	[M+H]+	C12H19NO6	274.12851					6.4E+05	0.4		
140	Hexadecasphinganine	[M+H]+	C16H35NO2	274.27406	1.4E+06	2.5	1.4E+06	0.2				
141	6-Phosphogluconic acid	[M+H]+	C6H13O10P	277.03191	2.4E+06	0.0	2.4E+06	0.0	6.3E+05	-0.3		
142	Xanthopterin-B2	[M+K]+	C9H10N4O4	277.03336	2.4E+06	-2.4			8.5E+05	-2.2		
143	methyl 4-[2-(2-formyl-vinyl)-3-hydroxy-5-oxo-cyclopentyl]-butanoate	[M+Na]+	C13H18O5	277.10464	2.7E+06	0.2	4.1E+06	0.6			2.9E+05	-1.6
144	Albendazole-2-aminosulfone	[M+K]+	C10H13N3O2S	278.03601	3.8E+05	-0.6	5.9E+05	1.1				
145	2-Ethylhexyl phthalate	[M+H]+	C16H22O4	279.15909	4.6E+05	1.3	4.6E+05	-0.8			1.6E+06	-2.1
146	(+)-12-methyl myristic acid	[M+K]+	C15H30O2	281.18774							2.5E+05	-1.9
147	Arborinine	[M+H]+	C16H15NO4	286.10738	3.5E+05	1.9	3.5E+05	-0.2			9.0E+04	-2.0
148	Nupharidine	[M+K]+	C15H23NO2	288.13604			1.1E+06	1.0				
149	Micropine	[M+Na]+	C16H27NO2	288.19340					1.2E+06	-2.7		
150	2(alpha-D-Mannosyl)-D-glycerate	[M+Na]+	C9H16O9	291.06865					6.3E+05	-2.4	2.3E+05	-1.5
151	2-p-Tolyl-5,6,7,8-tetrahydrobenzo[d]imidazo[2,1-b]thiazole	[M+Na]+	C16H16N2S	291.09264					1.1E+06	2.1		
152	Palmitoleic acid	[M+K]+	C16H30O2	293.18774	9.4E+05	-2.2	8.5E+05	0.8				
153	14-Methyl-8Z-hexadecen-1-ol	[M+K]+	C17H34O	293.22413			1.9E+06	1.5			2.5E+05	-1.0
154	Sugeonyl acetate	[M+Na]+	C17H24O3	299.16177			4.1E+05	0.5				
155	Stearidonic acid	[M+Na]+	C18H28O2	299.19815			4.5E+05	2.0				
156	Aspartylysine	[M+K]+	C10H19N3O5	300.09563					6.1E+05	-1.6		

Table S1: Untargeted metabolic profiling of Goji Berries hydroalcoholic extracts by ESI(+) FT-R MS

#	Putative annotation[a]	Ion	Formula (M)	m/z_{Theo}[b]	BL1[c]	ppm	BL2[c]	ppm	SL1[c]	ppm	SL2[c]	ppm
157	Dibutyl phthalate	[M+Na]+	C16H22O4	301.14103	5.4E+05	-0.5	1.0E+06	1.0				
158	Panaxytriol	[M+Na]+	C17H26O3	301.17742					7.2E+05	1.3		
159	Tributyl phosphate	[M+K]+	C12H27O4P	305.12786			5.3E+05	-1.1				
160	Stearic acid	[M+Na]+	C18H36O2	307.26075							3.8E+05	2.8
161	Hexadecanedioic acid	[M+Na]+	C16H30O4	309.20363	6.2E+05	-1.0	6.2E+05	-2.7				
162	(+)-14-methyl palmitic acid	[M+K]+	C17H34O2	309.21904			1.9E+06	-1.7			2.2E+05	77.0
163	Orotidine	[M+Na]+	C10H12N2O8	311.04859					5.4E+05	0.6		
164	Sempervirine	[M+K]+	C19H16N2	311.09451					5.5E+05	0.1	1.2E+05	2.3
165	4-(3-Methylbut-2-enyl)-L-tryptophan	[M+K]+	C16H20N2O2	311.11564					5.6E+05	-2.9		
166	Dibutyl phthalate	[M+K]+	C16H22O4	317.11497	1.3E+06	-1.0	1.6E+06	-0.3			3.5E+05	-2.4
167	N6,N6-Dimethyladenosine	[M+Na]+	C12H17N5O4	318.11727	5.8E+05	1.0	7.6E+05	-2.7				
168	N-methyl arachidonoyl amine	[M+H]+	C21H35NO	318.27914					1.5E+06	0.0		
169	Trimethylolpropane triacrylate	[M+Na]+	C15H20O6	319.11521	4.5E+05	-1.4						
170	Phenethylamine glucuronide	[M+Na]+	C14H19NO6	320.11046			8.7E+05	0.8				
171	1-Carbazol-9-yl-3-(3,5-dimethylpyrazol-1-yl)-propan-2-ol	[M+H]+	C20H21N3O	320.17574			1.0E+07	-1.8				
172	3,7-Dimethoxyflavone	[M+K]+	C17H14O4	321.05237					6.2E+05	2.4		
173	Arctolide	[M+H]+	C17H20O6	321.13326	1.1E+06	1.0	9.2E+05	-0.4				
174	Octanoylglucuronide	[M+H]+	C14H24O8	321.15439	5.2E+05	1.1	5.2E+05	-0.3	5.1E+05	0.3		
175	10-hydroxy-16-oxo-hexadecanoic acid	[M+K]+	C16H30O4	325.17757	1.1E+06	1.3	1.1E+06	-0.1				
176	14-Dihydroxycornestin	[M+Na]+	C16H20O6	331.11521					1.5E+06	-0.7		
177	Phytuberin	[M+K]+	C17H26O4	333.14627							1.4E+05	1.4
178	Steroid O-sulfate	[M+H]+	C18H24O4S	337.14681	5.3E+05	-2.3	4.0E+05	-1.0				
179	(9Z)-(7S,8S)-Dihydroxyoctadecenoic acid	[M+Na]+	C18H34O4	337.23493	1.3E+06	0.0					5.5E+05	0.3
180	5,2',6'-Trihydroxy-7-methoxyflavone	[M+K]+	C16H12O6	339.02655					5.2E+05	2.7	1.1E+05	1.2
181	1-(5-Phospho-D-ribosyl)-5-amino-4-imidazolecarboxylate	[M+H]+	C9H14N3O9P	340.05404					5.7E+05	-1.2		
182	Coptisine	[M+Na]+	C19H14NO4	343.08150					2.8E+06	2.2		

Table S1: Untargeted metabolic profiling of Goji Berries hydroalcoholic extracts by ESI(+) FT-R MS

#	Putative annotation[a]	Ion	Formula (M)	m/z$_{Theo}$[b]	BL1[c]	ppm	BL2[c]	ppm	SL1[c]	ppm	SL2[c]	ppm
183	12-HETE	[M+Na]+	C20H32O3	343.22437	6.4E+05	0.1					2.3E+06	-0.7
184	3',4'-Methylenedioxy-[2'',3'':7,8]furanoflavanone	[M+K]+	C18H12O5	347.03163			1.1E+06	2.9	2.8E+06	1.5		
185	Bis-D-fructose 2',1:2,1'-dianhydride	[M+Na]+	C12H20O10	347.09487					2.1E+06	2.2		
186	5,2'-Dihydroxy-7,4',5'-trimethoxyflavanone	[M+H]+	C18H18O7	347.11253					2.4E+06	2.8		
187	(-)-Phaseollinisoflavan	[M+Na]+	C20H20O4	347.12538					1.9E+06	-0.7	1.6E+05	-2.0
188	Tetraneurin E	[M+Na]+	C17H24O6	347.14651					1.4E+06	2.2		
189	Oxo-dihydroxy-octadecatrienoic acid	[M+Na]+	C18H28O5	347.18289					2.1E+06	0.0		
190	(3S,4S)-3-Hydroxytetradecane-1,3,4-tricarboxylate	[M+H]+	C17H30O7	347.20643			1.8E+06	-2.6				
191	(-)-Chimonanthine	[M+H]+	C22H26N4	347.22302					1.5E+06	2.2	3.7E+05	0.5
192	9,10-dihydroxy-13-hydroperoxy-11-octadecenoic acid	[M+H]+	C18H34O6	347.24282					1.6E+06	-1.7		
193	12Z-heneicosenoic acid	[M+Na]+	C21H40O2	347.29205					1.5E+06	-1.2		
194	Inosine 2'-phosphate	[M+H]+	C10H13N4O8P	349.05438			1.0E+06	-1.6				
195	2',4',6'-Trihydroxy-3'-prenyldihydrochalcone	[M+Na]+	C20H22O4	349.14103							1.6E+05	2.7
196	5'-Phosphoribosyl-N-formylglycinamide	[M+K]+	C8H15N2O9P	353.01468	4.1E+05	1.8					1.9E+05	0.0
197	Octadecanedioic acid	[M+K]+	C18H34O4	353.20887	3.1E+06	-0.7	9.1E+05	-0.9	4.7E+05	-2.0		
198	2-mercapto-octadecanoic acid	[M+K]+	C18H36O2S	355.20676								
199	1-O-Feruloyl-beta-D-glucose	[M+H]+	C16H20O9	357.11801					1.3E+06	-0.7		
200	Mugineic acid	[M+K]+	C12H20N2O8	359.08513					4.9E+05	-0.2		
201	Catharanthine	[M+Na]+	C21H24N2O2	359.17300							6.9E+05	2.9
202	Acronycine	[M+K]+	C20H19NO3	360.09965							2.1E+05	1.8
203	beta-Cyclopiazonate	[M+Na]+	C20H22N2O3	361.15226					6.2E+05	2.1		
204	Shiromodiol diacetate	[M+Na]+	C19H30O5	361.19854					5.3E+05	-0.7		
205	Disaccharide	[M+Na]+	C12H22O11	365.10543	5.5E+05	-2.1	7.3E+05	0.0	1.2E+06	0.2	8.2E+05	-2.9
206	N-Acetylmuramoyl-Ala	[M+H]+	C14H24N2O9	365.15546	6.9E+05	1.2	1.7E+06	-0.9			5.4E+05	2.0
207	3-Hydroxy-5,7,8-trimethoxyflavone	[M+K]+	C18H16O6	367.05785	3.9E+05	-0.2	4.2E+05	-1.2				
208	Corydaline	[M+H]+	C22H27NO4	370.20128							1.7E+05	-1.6

Table S1: Untargeted metabolic profiling of Goji Berries hydroalcoholic extracts by ESI(+) FT-R MS

#	Putative annotation[a]	Ion	Formula (M)	m/z_{Theo}[b]	BL1[c]	ppm	BL2[c]	ppm	SL1[c]	ppm	SL2[c]	ppm
209	19(R)-hydroxy-PGE1	[M+H]+	C20H34O6	371.24282			8.9E+05	-0.6	2.1E+06	-1.8		
210	19-norcholestanol	[M+H]+	C26H46O	375.36214			1.3E+06	0.8				
211	1-O-Feruloyl-beta-D-glucose	[M+Na]+	C16H20O9	379.09995							1.9E+05	-2.6
212	D-Maltose	[M+K]+	C12H22O11	381.07937	1.0E+06	0.2	1.4E+06	-1.2	1.4E+06	-0.9		
213	N1-(5-Phospho-a-D-ribosyl)-5,6-dimethylbenzimidazole	[M+Na]+	C14H19N2O7P	381.08221					1.3E+06	-1.2		
214	12a-Hydroxypachyrrhizone	[M+H]+	C20H14O8	383.07614							4.6E+05	-2.8
215	Aknadicine	[M+K]+	C19H23NO5	384.12078	6.0E+06	-2.2	1.2E+07	-1.0				
216	(+)-Eudesmin	[M+H]+	C22H26O6	387.18022					1.6E+06	2.0	2.3E+05	-2.0
217	(22E)-3alpha,12alpha-Dihydroxy-5beta-chol-22-en-24-oic Acid	[M+H]+	C24H38O4	391.28429			4.7E+05	-0.7				
218	Isopropamide	[M+K]+	C23H33N2O	392.22245					4.5E+05	-1.8		
219	5,7,3',4',5'-Pentahydroxy-3,6,8-trimethoxyflavone	[M+H]+	C18H16O10	393.08162	7.3E+05	-0.7					6.3E+05	2.3
220	Leukotriene B4 ethanolamide	[M+Na]+	C22H37NO4	402.26148					6.2E+05	-1.7		
221	5S-HETE di-endoperoxide	[M+H]+	C20H34O8	403.23264							2.6E+05	2.9
222	5-Methoxy-7-prenyloxy-8-C-prenylflavanone	[M+H]+	C26H30O4	407.22169			1.8E+06	-2.1				
223	Glucotropaeolin	[M+H]+	C14H19NO9S2	410.05740			5.7E+05	-1.1			1.8E+05	-1.9
224	Di-n-octyl phthalate	[M+Na]+	C24H38O4	413.26623	1.3E+06	-2.7	6.8E+05	-2.3			1.6E+06	-1.9
225	Glucoerucin	[M+H]+	C12H23NO9S3	422.06077	6.6E+05	-2.1						
226	5,7,3',4'-Tetrahydroxy-6,8-diprenylisoflavone	[M+H]+	C25H26O6	423.18022					1.9E+06	-1.5	4.5E+05	-2.5
227	(-)-epigallocatechin sulfate	[M+K]+	C15H13O10S	423.98610	1.3E+06	-1.6	1.3E+06	-1.4				
228	N-Methyl-2,3,7,8-tetramethoxy-5,6-dihydrobenzophenathridine-6-ethanoic acid	[M+H]+	C24H25NO6	424.17546					1.5E+07	-2.9		
229	Antiarone K	[M+Na]+	C22H26O7	425.15707							1.9E+05	1.1
230	Aspidoalbine	[M+H]+	C24H32N2O5	429.23840	5.1E+05	0.1	5.1E+05	0.3			6.8E+05	1.5
231	25-dihydroxy-19-nor-22-oxavitamin D3	[M+Na]+	C25H42O4	429.29753					7.1E+05	2.3		
232	5,7,8-Trihydroxyflavone 5-glucoside	[M+H]+	C21H20O10	433.11292	4.6E+05	-0.7						
233	Echimidine	[M+K]+	C20H31NO7	436.17321	6.2E+05	2.1						
234	Cryogenine	[M+H]+	C26H29NO5	436.21185	6.4E+05	2.7						

Table S1: Untargeted metabolic profiling of Goji Berries hydroalcoholic extracts by ESI(+) FT-R MS

#	Putative annotation[a]	Ion	Formula (M)	m/z_{Theo}[b]	BL1[c]	ppm	BL2[c]	ppm	SL1[c]	ppm	SL2[c]	ppm
235	Ancistrobrevine A	[M+H]+	C27H33NO4	436.24824	8.1E+05	-2.9						
236	Solanidine	[M+K]+	C27H43NO	436.29762	4.5E+05	-2.4						
237	Hallactone B	[M+H]+	C20H24O9S	441.12138	5.0E+05	-2.2	8.1E+05	-1.8				
238	2'',3''-Dihydroxylupinifolin	[M+H]+	C25H28O7	441.19078								
239	Lusitanicoside	[M+H]+	C21H30O10	443.19117							4.8E+05	2.8
240	24,25-trihydroxy-22-oxavitamin D3	[M+Na]+	C26H42O5	457.29245			3.9E+05	2.5				
241	5-Hydroxypseudobaptigenin 7-O-glucoside	[M+H]+	C22H20O11	461.10784					5.4E+05	3.0		
242	3-Hydroxy-11Z-octadecenoylcarnitine	[M+Na]+	C25H47NO5	464.33464					4.5E+05	0.3		
243	12-Dehydrotetracycline	[M+Na]+	C22H22N2O8	465.12684							3.4E+05	-2.6
244	Obliquine	[M+Na]+	C26H28N2O5	471.18904					4.8E+05	0.8		
245	N-arachidonoyl glutamic acid	[M+K]+	C25H39NO5	472.24598	1.3E+06	-1.0	3.6E+06	-0.3			8.1E+05	-0.2
246	25-dihydroxy-3-deoxy-3-thiavitamin D3 3-oxide	[M+K]+	C26H42O3S	473.24863	4.6E+05	-0.2	1.0E+06	2.1			3.1E+05	-1.4
247	1'',2''-Dihydro-2'-hydroxycycloosajin	[M+K]+	C26H28O6	475.15175					1.3E+06	-0.3		
248	DHAP(18:0)	[M+K]+	C21H41O7P	475.22215					1.4E+06	-2.1		
249	LPA(0:0/18:0)	[M+K]+	C21H43O7P	477.23780					4.7E+05	-1.7		
250	1-O-all-trans-retinoyl-beta-glucuronic acid	[M+H]+	C26H36O8	477.24829					4.6E+05	0.2		
251	27-Norcholestanehexol	[M+Na]+	C26H46O6	477.31866	4.6E+05	0.6	4.6E+05	1.6				
252	Monocyclic botryococcane	[M+H]+	C34H68	477.53938					1.1E+06	-2.3		
253	PG(16:1(9Z)/0:0)	[M+H]+	C22H43O9P	483.27175							3.7E+05	-0.2
254	Dihydroxy-24-oxo-23-azavitamin D2	[M+K]+	C27H43NO4	484.28237					7.9E+05	0.8		
255	LysoSM(d18:1)	[M+Na]+	C23H50N2O5P	488.33496					4.3E+06	0.0		
256	Leucettamol A	[M+Na]+	C30H52N2O2	495.39210					1.6E+06	-3.0		
257	Trihydroxy-5alpha-cholan-24-yl sulfate	[M+Na]+	C24H42O7S	497.25435					4.1E+05	-0.9	2.5E+05	1.6
258	N-Acetyl-O-demethylpuromycin	[M+H]+	C23H29N7O6	500.22521					7.0E+05	-0.1		
259	(-)-Jolkinol A	[M+Na]+	C29H36O6	503.24041					5.6E+05	-2.7		
260	3-Sulfodeoxycholic acid	[M+K]+	C24H40O7S	511.21263	3.6E+05	2.5	8.5E+05	-1.9				

Table S1: Untargeted metabolic profiling of Goji Berries hydroalcoholic extracts by ESI(+) FT-R MS

#	Putative annotation[a]	Ion	Formula (M)	m/z_{Theo}[b]	BL1[c]	ppm	BL2[c]	ppm	SL1[c]	ppm	SL2[c]	ppm
261	Epimedoside C	[M+H]+	C26H28O11	517.17044					4.1E+05	2.4	3.3E+05	2.9
262	Ophrysanin	[M+H]+	C23H21O14	522.10041					1.7E+06	1.2		
263	Trisaccharide	[M+Na]+	C18H32O16	527.15826	5.8E+05	-0.1	6.3E+05	1.3	5.9E+05	-2.4		
264	Artelastocarpin	[M+Na]+	C30H34O7	529.21967					1.2E+06	0.9	1.7E+05	-0.9
265	Aragusteroketal	[M+K]+	C31H54O4	529.36537					1.1E+06	-0.5		
266	32,35-anhydrobacteriohopaneterol	[M+H]+	C35H60O3	529.46152					1.4E+06	0.1		
267	Cassiamin C	[M+K]+	C30H18O8	545.06333			6.1E+05	2.3				
268	dTDP-3-methyl-4-oxo-2,6-dideoxy-L-glucose	[M+H]+	C17H26N2O14P2	545.09320			5.9E+05	0.5				
269	5,8-Dihydroxy-6,7,4'-trimethoxyflavone 8-glucoside	[M+K]+	C24H26O12	545.10559	4.7E+05	-1.3						
270	Estriol 3-sulfate 16-glucuronide	[M+H]+	C24H32O12S	545.16872	5.6E+05	2.0	7.4E+05	-0.3				
271	PG(18:3(6Z,9Z,12Z)/0:0)	[M+K]+	C24H43O9P	545.22763					4.6E+05	1.1		
272	Trilobolide	[M+Na]+	C27H38O10	545.23572	4.6E+05	-0.8						
273	PI(P-16:0/0:0)	[M+H]+	C25H49O11P	557.30853					7.1E+05	1.1		
274	Petunidin 3-(6''-acetylglucoside)	[M+K]+	C24H25O13	560.09268					5.7E+05	-2.9		
275	Eriocarpin	[M+H]+	C29H38O11	563.24869					5.2E+05	0.2	1.6E+05	-2.2
276	2-deoxy-20-hydroxyecdysone 22-phosphate	[M+Na]+	C27H45O9P	567.26934					4.6E+05	-0.9		
277	PG(20:5(5Z,8Z,11Z,14Z,17Z)/0:0)	[M+K]+	C26H43O9P	569.22763					4.3E+05	-1.6	3.5E+05	-0.5
278	(-)-Santiaguine	[M+H]+	C38H48N4O2	593.38500					1.2E+06	-0.5		
279	14:0 Cholesteryl ester	[M+H]+	C41H72O2	597.56051					1.8E+06	-0.4		
280	Quercetin 3-(2''-galloyl-alpha-L-arabinopyranoside)	[M+K]+	C27H22O15	625.05903					3.8E+05	-2.4	1.3E+05	0.6
281	Malvidin 3-O-beta-D-sambubioside	[M+H]+	C28H33O16	626.18414	4.1E+05	1.7	3.9E+05	2.5				
282	7''-O-Phosphohygromycin	[M+Na]+	C20H38N3O16P	630.18819					1.7E+06	1.4		
283	Peridinin	[M+H]+	C39H50O7	631.36293					7.8E+05	1.7		
284	PA(12:0/20:4(5Z,8Z,11Z,14Z))	[M+H]+	C35H61O8P	641.41768					3.9E+05	-0.5		
285	PA(12:0/17:1(9Z))	[M+K]+	C32H61O8P	643.37356					4.4E+05	-2.6		
286	PA(12:0/20:3(8Z,11Z,14Z))	[M+H]+	C35H63O8P	643.43333					5.4E+05	2.6		

Table S1: Untargeted metabolic profiling of Goji Berries hydroalcoholic extracts by ESI(+) FT-R MS

#	Putative annotation[a]	Ion	Formula (M)	m/z_{Theo}[b]	BL1[c]	ppm	BL2[c]	ppm	SL1[c]	ppm	SL2[c]	ppm
287	GDP-L-galactose	[M+K]+	C16H25N5O16P2	644.04031					4.0E+05	-1.0		
288	Juliflorine	[M+Na]+	C40H75N3O2	652.57515					3.8E+05	2.0		
289	2-(S-Glutathionyl)acetyl glutathione	[M+K]+	C22H34N6O13S2	693.12569					4.7E+05	0.0		
290	DG(18:1(11Z)/24:1(15Z)/0:0)	[M+H]+	C45H84O5	705.63915	1.1E+06	2.8						
291	Petunidin 3-glucoside-5-(6''-acetylglucoside)	[M+Na]+	C30H35O18	706.17156					9.4E+05	0.0		
292	PG(12:0/20:5(5Z,8Z,11Z,14Z,17Z))	[M+H]+	C38H65O10P	713.43881	9.8E+05	0.3					9.5E+04	-1.6
293	PC(10:0/21:0)	[M+H]+	C39H78NO8P	720.55378					4.2E+05	-0.9		
294	Apigenin 7-(2'',6''-di-p-coumarylglucoside)	[M+H]+	C39H32O14	725.18648	4.7E+05	2.6						
295	PC(12:0/20:4(5Z,8Z,11Z,14Z))	[M+H]+	C40H72NO8P	726.50683					5.6E+05	-1.1	1.3E+05	-2.2
296	Gomphrenin-V	[M+H]+	C34H34N2O16	727.19811	5.3E+05	0.6						
297	Isoliquiritigenin 4-O-(5'''-O-feruloyl)-apiofuranosyl-(1'''->2'')-glucoside	[M+H]+	C36H38O16	727.22326					5.3E+05	-0.9		
298	Adiantifoline	[M+H]+	C42H50N2O9	727.35891	4.3E+05	-2.3						
299	DG(20:4(5Z,8Z,11Z,14Z)/24:1(15Z)/0:0)	[M+H]+	C47H82O5	727.62350	4.2E+05	-2.1						
300	PG(12:0/21:0)	[M+H]+	C39H77O10P	737.53271					9.8E+05	2.2		
301	PG(O-16:0/17:2(9Z,12Z))	[M+Na]+	C39H75O9P	741.50409					4.5E+05	0.8		
302	Apigenin 7-rhamnoside-4'-rutinoside	[M+Na]+	C33H40O18	747.21069					5.0E+05	-0.5		
303	Phalluside-1	[M+Na]+	C41H75NO9	748.53340					7.5E+05	0.0		
304	3-Nonaprenyl-4-hydroxybenzoate	[M+H]+	C52H78O3	751.60237					5.6E+05	-0.7		
305	Amataine	[M+K]+	C43H48N4O6	755.32054					5.5E+05	0.0		
306	PC(12:0/22:1(11Z))	[M+H]+	C42H82NO8P	760.58508					4.1E+05	0.3		
307	Ophiopogonin B	[M+K]+	C39H62O12	761.38729					8.6E+05	-0.6		
308	PI(12:0/15:1(9Z))	[M+Na]+	C36H67O13P	761.42115	7.5E+05	1.2						
309	PI(O-16:0/16:0)	[M+H]+	C41H81O12P	797.55384					6.3E+05	0.5		
310	DG(22:2(13Z,16Z)/24:1(15Z)/0:0)	[M+K]+	C49H90O5	797.64199					4.6E+05	-0.7		
311	PI(13:0/20:4(5Z,8Z,11Z,14Z))	[M+H]+	C42H73O13P	817.48616					5.3E+05	-2.1		
312	PE(22:4(7Z,10Z,13Z,16Z)/P-18:0)	[M+K]+	C45H82NO7P	818.54605					4.3E+05	-0.1		

Table S1: Untargeted metabolic profiling of Goji Berries hydroalcoholic extracts by ESI(+) FT-R MS

#	Putative annotation[a]	Ion	Formula (M)	m/z$_{Theo}$[b]	BL1[c]	ppm	BL2[c]	ppm	SL1[c]	ppm	SL2[c]	ppm
313	PS(22:0/22:1(11Z))	[M+K]+	C50H96NO10P	940.64034	4.2E+05	1.4						
314	TG(16:0/20:2(11Z,14Z)/22:6(4Z,7Z,10Z,13Z,16Z,19Z))[iso6]	[M+K]+	C61H102O6	969.73080					3.5E+05	0.2		
315	(S)-3-Hydroxydodecanoyl-CoA	[M+Na]+	C33H58N7O18P3S	988.26641					4.2E+05	0.4		

a MG Monoacylglycerol; DG Diacylglycerol; TG Triacylglycerol; GalCer Galactosylceramide; PA Phosphatidic acid; PC Phosphatidylcholine; PE Phosphatidylethanolamine; PG Glycerophospholipids; PI Phosphatidylinositol; PS Phosphatidylserine; SQMG Glycosylmonoacylglycerols; DHAP Dihydroxyacetone phosphate; DNP Dinitrophenyl

b Theoretical m/z

c BL1 and BL2 stand for Big Lifeberry harvested in August and October, respectively; SL1 and SL2 stand for Sweet Lifeberry harvested in August and October, respectively.

Table S2: Untargeted metabolic profiling of Goji Berries hydroalcoholic extracts by ESI(-) FT-ICR MS

#	Putative annotation[a]	Ion	Formula	m/z_{Theo}[b]	BL1[c]	ppm	BL2[c]	ppm	SL1[c]	ppm	SL2[c]	ppm
1	Phosphonate	[M-H]-	PH3O3	80.97470			3.4E+05	-1.9				
2	1,4-Lactone	[M-H]-	C4H6O2	85.02950	1.3E+06	0.2						
3	(R)-Lactate	[M-H]-	C3H6O3	89.02442	4.3E+06	-0.1	3.6E+05	-1.8	4.0E+05	-1.5	6.7E+04	1.2
4	Dimethyl sulfone	[M-H]-	C2H6O2S	93.00157					5.8E+05	-2.2		
5	Orthophosphate	[M-H]-	H3PO4	96.96962	9.9E+05	-1.6	5.0E+05	2.0	4.7E+05	2.0	7.2E+04	1.3
6	Cyclohexylamine	[M-H]-	C6H13N	98.09752					3.8E+05	-3.0		
7	Malonic acid	[M-H]-	C3H4O4	103.00368							9.9E+04	3.0
8	3-Aminopropionaldehyde	[M+Cl]-	C3H7NO	108.02217	7.2E+05	2.4						
9	Glyoxylic acid	[M+Cl]-	C2H2O3	108.96980			7.6E+05	1.8				
10	Uracil	[M-H]-	C4H4N2O2	111.02000							4.4E+04	-1.7
11	Fumaric acid	[M-H]-	C4H4O4	115.00368	7.8E+05	-0.4						
12	3-Oxopentanoic acid	[M-H]-	C5H8O3	115.04007			4.2E+05	1.2			7.5E+04	-2.9
13	Succinic acid	[M-H]-	C4H6O4	117.01933	1.0E+06	-1.1	1.1E+06	0.8			9.0E+04	1.9
14	2-Hydroxyethanesulfonate	[M-H]-	C2H6O4S	124.99140	5.2E+05	-1.1						
15	Pyroglutamic acid	[M-H]-	C5H7NO3	128.03532							5.7E+04	2.8
16	Citraconic acid	[M-H]-	C5H6O4	129.01933			4.2E+05	0.9				
17	3-Methylindole	[M-H]-	C9H9N	130.06622					4.7E+05	2.2	1.0E+05	1.2
18	Asparagine	[M-H]-	C4H8N2O3	131.04622							6.1E+04	-1.2
19	Aspartate	[M-H]-	C4H7NO4	132.03023	8.0E+05	1.8	1.4E+06	-0.2				
20	L-Malic acid	[M-H]-	C4H6O5	133.01425	1.9E+07	0.1	3.0E+07	0.0	2.0E+06	-0.4	1.7E+05	-0.1
21	Threonic acid	[M-H]-	C4H8O5	135.02990	5.0E+05	-0.6	4.6E+05	1.6				
22	Proline betaine	[M-H]-	C7H13NO2	142.08735					9.5E+05	2.8		
23	(E)-2-Methylglutaconic acid	[M-H]-	C6H8O4	143.03498			1.6E+06	1.3			4.4E+05	-0.4
24	3-Dehydroxycarnitine	[M-H]-	C7H15NO2	144.10300	1.3E+06	1.0						
25	Solerol	[M-H]-	C6H10O4	145.05063	4.7E+05	0.9	4.8E+05	1.9			2.0E+05	-1.1

Table S2: Untargeted metabolic profiling of Goji Berries hydroalcoholic extracts by ESI(-) FT-ICR MS

#	Putative annotation[a]	Ion	Formula	m/z_{Theo}[b]	BL1[c]	ppm	BL2[c]	ppm	SL1[c]	ppm	SL2[c]	ppm
26	Cytosine	[M+Cl]-	C4H5N3O	146.01266							9.3E+04	0.9
27	L-Glutamic acid	[M-H]-	C5H9NO4	146.04588	4.8E+05	1.8	1.5E+06	1.8	7.1E+05	-2.9		
28	Citramalic acid	[M-H]-	C5H8O5	147.02990			5.3E+05	-0.4				
29	1-Methylhypoxanthine	[M-H]-	C6H6N4O	149.04688			5.7E+05	-1.0	9.8E+05	-0.1		
30	3-Amino-2-piperidone	[M+Cl]-	C5H10N2O	149.04871	4.5E+05	2.4	5.7E+05	-2.2	9.8E+05	-0.1		
31	Thiopurine	[M-H]-	C5H4N4S	151.00839			1.2E+06	-0.8			2.0E+05	-1.1
32	3-methyl-octanoic acid	[M-H]-	C9H18O2	157.12340	5.7E+05	0.0	1.4E+06	-0.5			4.9E+05	-2.8
33	Nicotine imine	[M-H]-	C10H13N2	160.10060					8.1E+05	1.7		
34	(2R,3S)-2,3-Dimethylmalate	[M-H]-	C6H10O5	161.04555	5.5E+05	0.8	2.6E+06	1.1				
35	2-Dehydro-D-xylonate	[M-H]-	C5H8O6	163.02481			6.4E+05	-1.2				
36	5-methyl-5-hexenoic acid	[M+Cl]-	C7H12O2	163.05313	4.3E+05	-2.4	1.6E+07	2.6				
37	Rhamnose	[M-H]-	C6H12O5	163.06120			1.2E+07	0.5				
38	L-Lysine 1,6-lactam	[M+Cl]-	C6H12N2O	163.06436					1.6E+07	-1.4		
39	Arabinonic acid	[M-H]-	C5H10O6	165.04046							4.2E+04	-0.9
40	Phenylacetothiohydroximate	[M-H]-	C8H9NOS	166.03321			6.1E+05	-1.4				
41	2-Furoylglycine	[M-H]-	C7H7NO4	168.03023			1.1E+06	1.8				
42	Diphenylamine	[M-H]-	C12H11N	168.08187			1.1E+06	-2.2				
43	2-Octenedioic acid	[M-H]-	C8H12O4	171.06628	4.1E+05	1.2	1.3E+06	0.3				
44	Decanoic acid	[M-H]-	C10H20O2	171.13905	3.3E+05	2.7	8.4E+05	2.6			4.9E+05	0.3
45	Dehydroascorbic acid	[M-H]-	C6H6O6	173.00916	3.2E+05	-1.5	8.7E+05	-0.5				
46	2-Propylglutaric acid	[M-H]-	C8H14O4	173.08193	3.2E+05	-1.5					2.1E+05	0.6
47	Monodehydroascorbate	[M-H]-	C6H7O6	174.01699					8.2E+05	1.8		
48	Canavanine	[M-H]-	C5H12N4O3	175.08366	3.8E+05	-2.7						
49	Caffeic acid quinone	[M-H]-	C9H6O4	177.01933							7.5E+04	2.8
50	2-Octenoic acid	[M+Cl]-	C8H14O2	177.06878	5.2E+05	-1.2					6.1E+04	-0.6
51	(E)-2-Methylglutaconic acid	[M+Cl]-	C6H8O4	179.01166					8.9E+05	1.4		

Table S2: Untargeted metabolic profiling of Goji Berries hydroalcoholic extracts by ESI(-) FT-ICR MS

#	Putative annotation[a]	Ion	Formula	m/z_{Theo}[b]	BL1[c]	ppm	BL2[c]	ppm	SL1[c]	ppm	SL2[c]	ppm
52	D-Aldose	[M-H]-	C6H12O6	179.05611	9.3E+06	-0.7	1.3E+07	-0.5	2.5E+06	1.2	2.5E+05	0.8
53	Paraxanthine	[M-H]-	C7H8N4O2	179.05745			4.4E+06	-2.5			2.5E+05	0.7
54	O-Carbamoyl-L-serine	[M+Cl]-	C4H8N2O4	183.01781					9.8E+05	0.9		
55	L-Methionine	[M+Cl]-	C5H11NO2S	184.02045							5.9E+04	-1.0
56	2-Amino-4-nitrotoluene	[M+Cl]-	C7H8N2O2	187.02798					8.6E+05	-0.4		
57	Citric acid	[M-H]-	C6H8O7	191.01973	4.5E+06	0.3	4.3E+07	0.6	2.8E+06	0.7		
58	Quinic acid	[M-H]-	C7H12O6	191.05611	4.1E+06	0.4	8.9E+06	0.7	6.4E+05	0.5		
59	Galacturonic acid	[M-H]-	C6H10O7	193.03538	2.1E+06	2.9	2.5E+06	1.7	8.0E+05	2.6		
60	Phenanthrene-9,10-oxide	[M-H]-	C14H10O	193.06589	1.4E+06	0.2						
61	1-O-Methyl-myo-inositol	[M-H]-	C7H14O6	193.07176	1.7E+06	2.2						
62	Caffeine	[M-H]-	C8H10N4O2	193.07310	1.5E+06	-0.7						
63	2-methyl-octanoic acid	[M+Cl]-	C9H18O2	193.10008			1.4E+06	0.2				
64	Tetrahydroxypteridine	[M-H]-	C6H4N4O4	195.01598					9.0E+05	2.3		
65	2-Propylsuccinic acid	[M+Cl]-	C7H12O4	195.04296					1.0E+06	-0.8		
66	D-Gluconic acid	[M-H]-	C6H12O7	195.05103	5.7E+05	0.6	8.5E+05	0.5				
67	9-acetyl pelargonic acid	[M-H]-	C11H20O3	199.13397					2.1E+06	2.9		
68	Lauric acid	[M-H]-	C12H24O2	199.17035	3.6E+05	0.6					7.8E+05	0.2
69	Sebacic acid	[M-H]-	C10H18O4	201.11323			8.0E+05	1.1				
70	N5-(L-1-Carboxyethyl)-L-ornithine	[M-H]-	C8H16N2O4	203.10373			8.7E+05	2.9				
71	2-Methylcitric acid	[M-H]-	C7H10O7	205.03538	4.1E+05	2.5	1.1E+06	1.7				
72	Thiolactomycin	[M-H]-	C11H14O2S	209.06417			1.0E+06	2.8	1.1E+06	2.9		
73	1,3,7-Trimethyluric acid	[M-H]-	C8H10N4O3	209.06801	3.5E+05	-0.1						
74	N-(3-oxo-hexanoyl)-homoserine lactone	[M-H]-	C10H15NO4	212.09283	3.2E+05	-2.7	1.2E+06	-2.9			6.1E+04	-2.9
75	O-Phospho-4-hydroxy-L-threonine	[M-H]-	C4H10NO7P	214.01221	1.8E+06	-2.9						
76	Aldohexose	[M+Cl]-	C6H12O6	215.03279	2.1E+07	-0.1	3.0E+07	-1.2	2.5E+07	0.6	3.0E+06	1.9
77	Theophylline	[M+Cl]-	C7H8N4O2	215.03413			2.4E+07	0.5				

Table S2: Untargeted metabolic profiling of Goji Berries hydroalcoholic extracts by ESI(-) FT-ICR MS

#	Putative annotation[a]	Ion	Formula	m/z_{Theo}[b]	BL1[c]	ppm	BL2[c]	ppm	SL1[c]	ppm	SL2[c]	ppm
78	4-Amino-2-methyl-5-phosphomethylpyrimidine	[M-H]-	C6H10N3O4P	218.03362	6.5E+05	-1.3	6.5E+05	1.7	6.8E+05	-0.9		
79	Pantothenate	[M-H]-	C9H17NO5	218.10340	4.0E+05	-1.8	1.4E+06	-0.6				
80	Phenylacetylglycine dimethylamide	[M-H]-	C12H16N2O2	219.11390	8.1E+05	1.4						
81	6-Benzylaminopurine	[M-H]-	C12H11N5	224.09417					1.2E+06	0.4		
82	5-Acetylamino-6-formylamino-3-methyluracil	[M-H]-	C8H10N4O4	225.06293	8.7E+05	-1.8	1.8E+06	-2.9				
83	Myristoleic acid	[M-H]-	C14H26O2	225.18600							1.0E+05	2.7
84	Myristic acid	[M-H]-	C14H28O2	227.20165	4.9E+05	-1.7	2.0E+06	-0.4	1.1E+06	1.5	1.8E+06	2.0
85	Nitro-L-arginine methyl ester	[M-H]-	C7H15N5O4	232.10513			2.4E+06	2.9				
86	Glycosminine	[M-H]-	C15H12N2O	235.08769					7.0E+05	2.0		
87	Benzoyl phosphate	[M+Cl]-	C7H7O5P	236.97251	3.5E+05	-0.2						
88	3-Deoxy-D-manno-octulosonate	[M-H]-	C8H14O8	237.06159	3.0E+05	2.1						
89	N6-(delta2-Isopentenyl)-adenine	[M+Cl]-	C10H13N5	238.08650			1.9E+06	1.4				
90	L-Tryptophan	[M+Cl]-	C11H12N2O2	239.05928	3.8E+05	-2.7						
91	6-Deoxy-5-ketofructose 1-phosphate	[M-H]-	C6H11O8P	241.01188	7.6E+05	2.6	1.0E+06	1.5	1.5E+06	-0.2		
92	6E,8E,14E-Hexadecatriene-10,12-diynoic acid	[M-H]-	C16H18O2	241.12340			3.5E+06	-2.5				
93	(+)-12-methyl myristic acid	[M-H]-	C15H30O2	241.21730	4.7E+05	-0.3	1.9E+06	-1.8			1.1E+06	1.1
94	Anthocyanin	[M+Cl]-	C15H11O	242.05039	1.1E+06	0.7	3.1E+06	1.6				
95	1,3,5-Trihydroxyxanthone	[M-H]-	C13H8O5	243.02990	1.1E+06	-1.1						
96	10,12-Tetradecadienal	[M+Cl]-	C14H24O	243.15212	6.8E+05	1.4						
97	Indoxyl sulfate	[M+Cl]-	C8H7NO4S	247.97898					8.8E+06	0.4		
98	Succinyl proline	[M+Cl]-	C9H13NO5	250.04877	3.3E+05	0.6						
99	Kinetin	[M+Cl]-	C10H9N5O	250.05011	6.0E+05	0.4						
100	N-Feruloylglycine	[M-H]-	C12H13NO5	250.07210	6.3E+05	-0.5						
101	4-Methylumbelliferyl acetate	[M+Cl]-	C12H10O4	253.02731			7.3E+05	0.8				
102	Palmitoleic acid	[M-H]-	C16H30O2	253.21730	6.6E+05	0.0						
103	Phenylgalactoside	[M-H]-	C12H16O6	255.08741			9.1E+05	0.1				

Table S2: Untargeted metabolic profiling of Goji Berries hydroalcoholic extracts by ESI(-) FT-ICR MS

#	**Putative annotation**[a]	**Ion**	**Formula**	**m/z_{Theo}**[b]	**BL1**[c]	**ppm**	**BL2**[c]	**ppm**	**SL1**[c]	**ppm**	**SL2**[c]	**ppm**
104	Palmitic acid	[M-H]-	C16H32O2	255.23295	1.3E+06	0.1	3.6E+06	-1.6	1.7E+06	-2.4	5.8E+06	-2.1
105	4-Methyl-5-(2-phosphoethyl)-thiazole	[M+Cl]-	C6H10NO4PS	257.97622					1.6E+06	-1.2		
106	N-Sulfo-D-glucosamine	[M-H]-	C6H13NO8S	258.02891					9.6E+05	2.4		
107	N-Acetyl-L-tyrosine	[M+Cl]-	C11H13NO4	258.05386					1.7E+06	0.7		
108	Skimmianine	[M-H]-	C14H13NO4	258.07718					1.8E+06	1.2	6.2E+04	2.6
109	Hexanoylcarnitine	[M-H]-	C13H25NO4	258.17108					2.0E+06	-0.9		
110	4Z,7Z,10Z-octadecatrienenitrile	[M-H]-	C18H29N	258.22272					1.3E+06	1.0		
111	Methyl jasmonate	[M+Cl]-	C13H20O3	259.11065							5.5E+04	2.3
112	Mannitol-1-phosphate	[M-H]-	C6H15O9P	261.03700			1.5E+06	2.1			5.2E+05	0.3
113	1,8-Diazacyclotetradecane-2,9-dione	[M+Cl]-	C12H22N2O2	261.13753			8.0E+05	-1.9				
114	gamma-Fagarine	[M+Cl]-	C13H11NO3	264.04329			7.0E+05	-2.8				
115	Oxycarboxin	[M-H]-	C12H13NO4S	266.04925					1.0E+06	0.1		
116	S-Ribosyl-L-homocysteine	[M-H]-	C9H17NO6S	266.07038					1.1E+06	0.4		
117	Neuraminic acid	[M-H]-	C9H17NO8	266.08814					1.3E+06	-0.6		
118	Acetylcarnosine	[M-H]-	C11H15N4O4	266.10205					9.4E+05	-2.9		
119	(-)-Caaverine	[M-H]-	C17H17NO2	266.11865					1.3E+06	2.7		
120	Dimethoxyhydroxycaffein	[M-H]-	C10H14N4O5	269.08914	3.3E+07	-1.7	1.5E+07	0.4	3.4E+06	1.3		
121	Doxylamine	[M-H]-	C17H22N2O	269.16594			8.0E+05	1.1				
122	10E,12Z-Hexadecadienal	[M+Cl]-	C16H28O	271.18342					1.3E+06	-1.7	7.5E+04	-1.9
123	8-Allyl-2-phenyl-8H-1,3a,8-triaza-cyclopenta[a]indene	[M-H]-	C18H15N3	272.11932			7.7E+05	0.2				
124	4'-O-Methylnorbelladine	[M-H]-	C16H19NO3	272.12922			8.6E+05	1.7				
125	10Z,13Z,16Z-nonadecatrienenitrile	[M-H]-	C19H31N	272.23837	6.1E+05	2.3						
126	Phosphoallohydroxy-L-lysine	[M+Cl]-	C6H15N2O6P	277.03617	1.3E+07	0.5	1.7E+07	2.8	1.5E+06	-0.3		
127	Oleic acid	[M-H]-	C18H34O2	281.24860	3.8E+05	0.3	1.1E+06	-2.4				
128	5-O-Methylgenistein	[M-H]-	C16H12O5	283.06120	6.5E+05	2.4	7.4E+05	-2.6				
129	Stearic acid	[M-H]-	C18H36O2	283.26425	5.4E+05	2.1	1.3E+06	-1.2	1.2E+06	2.7	5.8E+06	-0.6

Table S2: Untargeted metabolic profiling of Goji Berries hydroalcoholic extracts by ESI(-) FT-ICR MS

#	Putative annotation[a]	Ion	Formula	m/z_{Theo}[b]	BL1[c]	ppm	BL2[c]	ppm	SL1[c]	ppm	SL2[c]	ppm
130	2,6-Naphthalenedisulfonic acid	[M-H]-	C10H8O6S2	286.96895	4.9E+05	-2.3						
131	2-[3-Ethyl-5-(4-methoxyphenyl)-1H-pyrazol-4-yl]phenol	[M-H]-	C18H18N2O2	293.12955			8.3E+05	-1.3				
132	18:2(5Z,9Z)(16Me)	[M-H]-	C19H34O2	293.24860			4.1E+06	-0.4				
133	1,4-benzothiazine-o-quinonimine	[M-H]-	C12H11N2O5S	294.03159			1.1E+06	-0.5				
134	N6,N6-Dimethyladenosine	[M-H]-	C12H17N5O4	294.12078	7.9E+05	-2.2	1.2E+06	-2.2	1.1E+06	-0.9		
135	L-alpha-glutamyl-L-hydroxyproline	[M+Cl]-	C10H16N2O6	295.07024	3.4E+06	1.5	5.1E+06	1.5	9.9E+05	-2.7		
136	Albendazole sulfone	[M-H]-	C12H15N3O4S	296.07105	4.1E+05	-1.5						
137	D-Ornaline	[M+Cl]-	C10H18N2O6	297.08589	1.1E+06	-1.9	9.1E+05	-0.2				
138	5-Hydroxy-7-methoxy-6,8-di-C-methylflavanone	[M-H]-	C18H18O4	297.11323					1.2E+06	2.7		
139	5,7,4'-Trimethoxyflavan	[M-H]-	C18H20O4	299.12888							1.4E+05	-1.1
140	11alpha-Hydroxyandrosta-1,4-diene-3,17-dione	[M-H]-	C19H24O3	299.16527			4.6E+05	-0.9				
141	5-Methyl-6-hydroxyluteolinidin	[M-H]-	C16H15O6	302.07959							1.2E+05	-0.5
142	O-Acetylated sialic acid	[M-H]-	C11H19NO9	308.09870	4.1E+05	-1.2	7.8E+05	0.6			1.9E+05	-2.7
143	Botrydial	[M-H]-	C17H26O5	309.17075	5.1E+05	-0.1	1.3E+06	-0.7				
144	alpha-Obscurine	[M+Cl]-	C17H26N2O	309.17392	5.1E+05	1.4	1.3E+06	-1.8				
145	4-Hydroxycinnamyl alcohol 4-D-glucoside	[M-H]-	C15H20O7	311.11363	3.1E+06	0.5	1.4E+06	-2.9				
146	Angustine	[M-H]-	C20H15N3O	312.11424	5.9E+05	1.8	4.6E+05	-1.7				
147	Rosinidin	[M-H]-	C17H15O6	314.07959	1.3E+06	2.4						
148	N-Glycolyl-D-mannosamine 6-phosphate	[M-H]-	C8H16NO10P	316.04391			5.7E+05	0.6				
149	Arctolide	[M-H]-	C17H20O6	319.11871			4.7E+05	2.6				
150	N-oleoyl ethanolamine	[M-H]-	C20H39NO2	324.29080			5.7E+05	2.1	8.9E+05	-1.3		
151	MG(0:0/16:1(9Z)/0:0)	[M-H]-	C19H36O4	327.25408	4.3E+05	2.8						
152	22:4(4Z,7Z,10Z,13E)	[M-H]-	C22H36O2	331.26425					1.3E+06	-0.1	1.3E+05	3.0
153	1-Methylguanosine	[M+Cl]-	C11H15N5O5	332.07672			4.5E+05	-0.6				
154	9,13-cis-retinoate	[M+Cl]-	C20H27O2	334.17051			2.2E+06	2.7				
155	Lobelanine	[M-H]-	C22H25NO2	334.18125			1.1E+06	-1.0				

Table S2: Untargeted metabolic profiling of Goji Berries hydroalcoholic extracts by ESI(-) FT-ICR MS

#	Putative annotation[a]	Ion	Formula	m/z_{Theo}[b]	BL1[c]	ppm	BL2[c]	ppm	SL1[c]	ppm	SL2[c]	ppm
156	Pseudopurpurin	[M+Cl]-	C15H8O7	334.99640			1.2E+06	0.9				
157	N-Acetyl-D-galactosamine 1-phosphate	[M+Cl]-	C8H16NO9P	336.02567							3.8E+05	-1.0
158	6-(2-Amino-2-carboxyethyl)-7,8-dioxo-hexahydroquinoline-2,4-dicarboxylate	[M-H]-	C14H14N2O8	337.06774	5.6E+05	1.0						
159	5'-Hydroxy-2'-methoxy-3,4-methylenedioxyfurano[2'',3'':4',3']chalcone	[M-H]-	C19H14O6	337.07176	5.2E+05	2.3	2.9E+06	-1.3			6.3E+05	-1.8
160	3''-Deamino-3''-oxonicotianamine	[M+Cl]-	C12H18N2O7	337.08080			9.8E+05	0.9	4.7E+06	1.3		
161	D-Glucosyl-D-mannose	[M-H]-	C12H22O11	341.10894					1.6E+06	2.9	3.1E+06	-1.5
162	13(S)-HpOTrE(gamma)	[M+Cl]-	C18H30O4	345.18381			6.1E+05	2.0				
163	7Z-eicosenoic acid	[M-H]-	C20H38O2	345.25658							2.4E+05	-0.9
164	2-(alpha-D-Mannosyl)-3-phosphoglycerate	[M-H]-	C9H17O12P	347.03849					1.6E+06	-2.4		
165	3',4',5'-Trimethoxyflavone	[M+Cl]-	C18H16O5	347.06918					1.2E+06	0.8		
166	Arabino-galactose	[M+Cl]-	C11H20O10	347.07505					1.2E+06	0.0		
167	Machaerol C	[M-H]-	C18H20O7	347.11363					1.1E+06	2.0		
168	trans-Anhydrotephrostachin	[M-H]-	C22H20O4	347.12888					1.3E+06	0.0		
169	Fruticosonine	[M+Cl]-	C20H28N2O	347.18957					1.3E+06	3.0		
170	Estradiol-17beta 3-sulfate	[M-H]-	C18H24O5S	351.12717	4.7E+05	0.7						
171	Chlorogenic acid	[M-H]-	C16H18O9	353.08781	1.3E+06	-2.9	4.7E+06	2.8				
172	Pantetheine 4'-phosphate	[M-H]-	C11H23N2O7PS	357.08908					1.3E+06	-0.6		
173	Galactosylhydroxylysine	[M+Cl]-	C12H24N2O8	359.12267	2.9E+07	-1.9			9.6E+05	-1.9		
174	3-Methoxytyramine-betaxanthin	[M-H]-	C18H20N2O6	359.12486			1.7E+07	1.5			1.5E+05	0.3
175	Monocrotaline	[M+Cl]-	C16H23NO6	360.12194	4.2E+06	1.3	6.0E+06	1.2				
176	5,7,3'-Trihydroxy-6,4',5'-trimethoxyflavanone	[M-H]-	C18H18O8	361.09289					1.7E+06	-3.0		
177	(+)-18-methyl-eicosanoic acid	[M+Cl]-	C21H42O2	361.28788					3.2E+06	-0.4		
178	C25:2 6,7-Epoxy highly branched isoprenoid	[M-H]-	C25H46O	361.34759					2.4E+06	-2.3		
179	11-hydroperoxy-12,13-epoxy-9-octadecenoic acid	[M+Cl]-	C18H32O5	363.19438	4.2E+05	2.9						
180	NAc-DNP-Cys	[M+Cl]-	C11H11N3O7S	364.00117			8.8E+05	2.8				
181	1-O-Galloyl-beta-D-glucose	[M+Cl]-	C13H16O10	367.04375	5.1E+05	2.0	9.5E+05	-0.5	2.4E+06	2.6		

Table S2: Untargeted metabolic profiling of Goji Berries hydroalcoholic extracts by ESI(-) FT-ICR MS

#	Putative annotation[a]	Ion	Formula	m/z_{Theo}[b]	BL1[c]	ppm	BL2[c]	ppm	SL1[c]	ppm	SL2[c]	ppm
182	Isopentenyl adenosine	[M+Cl]-	C15H21N5O4	370.12876	5.0E+05	-2.3						
183	24-norcholesterol	[M-H]-	C26H44O	371.33194					1.1E+06	2.7		
184	Arachidonoylmorpholine	[M-H]-	C24H39NO2	372.29080					1.3E+06	0.6		
185	Alamarine	[M+Cl]-	C19H18N2O4	373.09606	2.5E+06	2.6	1.4E+06	2.6				
186	Nicotine glucuronide	[M+Cl]-	C16H22N2O6	373.11719			8.5E+05	-0.7				
187	5-Hydroxytryptophol glucuronide	[M+Cl]-	C16H21NO7	374.10120	3.1E+05	0.6						
188	Pentamidine	[M+Cl]-	C19H24N4O2	375.15933	4.4E+05	0.9						
189	Methantheline	[M+Cl]-	C21H26NO3	375.16067	4.4E+05	-2.7						
190	Dolichyl diphosphate	[M+Cl]-	C12H26O7P2	379.08478					1.0E+06	-2.4		
191	Cis-zeatin-O-glucoside	[M-H]-	C16H23N5O6	380.15756							1.1E+05	2.2
192	Callytriol C	[M+Cl]-	C23H24O3	383.14195			4.7E+05	-2.2			2.4E+05	2.2
193	5,7,2',5'-Tetrahydroxy-3,4'-dimethoxyflavone 5'-acetate	[M-H]-	C19H16O9	387.07216					1.1E+06	0.6		
194	(+)N-(1S-methyl-2-hydroxy-ethyl) alpha,alpha-dimethylarachidonoyl amine	[M-H]-	C25H43NO2	388.32210					1.3E+06	-0.1		
195	Miraxanthin-I	[M+Cl]-	C14H18N2O7S	393.05287	8.6E+05	-2.5	1.3E+06	-1.0	3.2E+06	-2.1		
196	Pantetheine 4'-phosphate	[M+Cl]-	C11H23N2O7PS	393.06576			2.2E+06	-1.7				
197	Triglochinin	[M+Cl]-	C14H17NO10	394.05465			4.1E+05	-0.3	1.3E+06	2.1		
198	3-Methoxytyramine-betaxanthin	[M+Cl]-	C18H20N2O6	395.10154			1.8E+06	-0.1				
199	3-(2,4-Cyclopentadien-1-ylidene)pregn-4-en-20-one	[M+Cl]-	C26H34O	397.23037							2.6E+05	-0.5
200	Estradiol-17-phenylpropionate	[M-H]-	C27H32O3	403.22787					1.2E+06	-1.5		
201	6-Hydroxyprotopine	[M+Cl]-	C20H19NO6	404.09064					1.4E+06	2.2		
202	Fraxin	[M+Cl]-	C16H18O10	405.05940			4.7E+05	0.4			8.6E+04	-0.3
203	MG(0:0/22:4(7Z,10Z,13Z,16Z)/0:0)	[M-H]-	C25H42O4	405.30103					1.1E+06	-3.0	2.5E+05	-1.3
204	Gossypetin 7-methyl ether 8-acetate	[M+Cl]-	C18H14O9	409.03318			1.3E+06	-0.9				
205	3,5-Dihydroxy-6,7,8-trimethoxy-3',4'-methylenedioxyflavone	[M+Cl]-	C19H16O9	423.04883					1.2E+06	-2.4		
206	Tuberonic acid glucoside	[M+Cl]-	C18H28O9	423.14273							4.7E+04	0.0
207	Acetyl adenylate	[M+Cl]-	C12H16N5O8P	424.04305					8.1E+05	-1.6		

Table S2: Untargeted metabolic profiling of Goji Berries hydroalcoholic extracts by ESI(-) FT-ICR MS

#	Putative annotation[a]	Ion	Formula	m/z_{Theo}[b]	BL1[c]	ppm	BL2[c]	ppm	SL1[c]	ppm	SL2[c]	ppm
208	Coleone P	[M+Cl]-	C22H30O6	425.17364			8.1E+05	2.9				
209	Abscisic acid glucose ester	[M-H]-	C21H30O9	425.18171	3.3E+05	-1.4						
210	Allochenodeoxycholic acid	[M+Cl]-	C24H40O4	427.26206	4.6E+05	0.6						
211	(5Z)-(3S)-1alpha,25-dihydroxy-3-deoxy-3-thiavitamin D3 3-oxide	[M-H]-	C26H42O3S	433.27819			1.7E+06	2.4				
212	Flavonol 3-O-D-glycoside	[M+Cl]-	C21H20O8	435.08522							4.4E+04	2.0
213	PA(16:1(9Z)/0:0)	[M+Cl]-	C19H37O7P	443.19709							7.2E+04	2.2
214	Mycolipenic acid (C27)	[M+Cl]-	C27H52O2	443.36613					1.3E+06	0.7		
215	dIDP	[M+Cl]-	C10H14N4O10P2	446.98792			4.6E+05	-2.8				
216	1D-myo-Inositol 1,3,4-trisphosphate	[M+Cl]-	C6H15O15P3	454.93178			8.3E+05	0.7				
217	Enantiomultijugin	[M+Cl]-	C24H22O7	457.10595			2.2E+06	0.4				
218	DHAP(18:0e)	[M+Cl]-	C21H43O6P	457.24913	4.3E+05	-0.1						
219	Staurosporine	[M-H]-	C28H26N4O3	465.19321					7.6E+05	0.9		
220	Cephaeline	[M-H]-	C28H38N2O4	465.27588					1.8E+06	-0.5		
221	Dichamanetin	[M-H]-	C29H24O6	467.15001					8.0E+05	1.2		
222	Marchantin A	[M+Cl]-	C28H24O5	475.13178			6.6E+05	-1.9	7.6E+05	1.8		
223	(24R)-24-Methylcycloarta-25-en-3-beta-ol	[M+Cl]-	C31H52O	475.37122					1.3E+06	1.6		
224	CTP	[M-H]-	C9H16N3O14P3	481.97724					2.2E+06	-0.9		
225	PA(P-20:0/0:0)	[M+Cl]-	C23H47O6P	485.28043	3.7E+05	-1.0			1.8E+06	-1.4		
226	Cyanidin 3-(4''-acetylglucoside)	[M-H]-	C23H23O12	490.11167	3.8E+05	0.1	3.5E+06	2.2				
227	5,10-Methenyltetrahydrofolic acid	[M+Cl]-	C20H21N7O6	490.12473			1.8E+06	-3.0				
228	20-Hydroxy-leukotriene E4	[M+Cl]-	C23H37NO6S	490.20356	1.4E+06	-2.2						
229	(+)-3beta-Hydroxy-ursan-28-oic acid	[M+Cl]-	C30H50O3	493.34540					7.6E+05	-2.3		
230	7-Methylthioheptyl glucosinolate	[M+Cl]-	C15H29NO9S3	498.06985					8.6E+05	-2.2		
231	N-Acetyl-O-demethylpuromycin	[M-H]-	C23H29N7O6	498.21066	6.2E+05	-2.3						
232	Quercetin 3-galactoside	[M+Cl]-	C21H20O12	499.06488			5.0E+05	3.0				

Table S2: Untargeted metabolic profiling of Goji Berries hydroalcoholic extracts by ESI(-) FT-ICR MS

#	Putative annotation[a]	Ion	Formula	m/z_{Theo}[b]	BL1[c]	ppm	BL2[c]	ppm	SL1[c]	ppm	SL2[c]	ppm
233	1-(2-Hydroxy-3,4,5,6-tetramethoxyphenyl)-3-(2,3,4,6-tetramethoxyphenyl)-2-propen-1-one	[M+Cl]-	C23H28O10	499.13765	5.4E+05	0.1						
234	Cromolyn	[M+Cl]-	C23H16O11	503.03866	4.1E+05	-2.1						
235	Arachidonyl carnitine	[M-H]-	C31H54NO4	503.39801			3.9E+05	-1.5			1.4E+05	-1.0
236	Emetamine	[M+Cl]-	C29H36N2O4	511.23691			1.2E+06	2.7				
237	Isorhamnetin 7-alpha-D-Glucosamine [Flavones and Flavonols	[M+Cl]-	C22H23NO11	512.09651			7.0E+05	-2.1				
238	PS(15:1(9Z)/0:0)	[M+Cl]-	C21H40NO9P	516.21347	3.9E+05	2.2						
239	N-Acetyl-leukotriene E4	[M+Cl]-	C25H39NO6S	516.21921							8.5E+04	-2.4
240	Glucosylgalactosyl hydroxylysine	[M+Cl]-	C18H34N2O13	521.17549			1.1E+06	1.4				
241	Mascaroside	[M-H]-	C26H36O11	523.21849					1.3E+06	2.8		
242	2',4',6',3,4-Pentahydroxy-3'-geranyl-5-prenyldihydrochalcone	[M+Cl]-	C30H38O6	529.23624					8.1E+05	-0.3	9.3E+04	-2.9
243	PI(13:0/0:0)	[M-H]-	C22H43O12P	529.24194					7.8E+05	-1.7		
244	35-aminobacteriohopane-32,33,34-triol	[M-H]-	C35H63NO3	544.47352	4.4E+05	-0.5						
245	Decoside	[M-H]-	C30H42O9	545.27561	5.1E+05	0.0						
246	all-trans-retinyl linolate	[M-H]-	C38H58O2	545.43640	5.4E+05	2.8						
247	Mycalamide B	[M+Cl]-	C25H43NO10	552.25810					1.3E+06	0.6		
248	PG(10:0/10:0)	[M-H]-	C26H51O10P	553.31471					1.1E+06	2.2	5.5E+04	-2.9
249	6,8a-Seco-6,8a-deoxy-5-oxoavermectin ''1b'' aglycone	[M-H]-	C33H46O7	553.31708					1.1E+06	-1.1		
250	Desmosine	[M+Cl]-	C24H40N5O8	561.25709	3.4E+05	0.0						
251	DG(14:0/15:0/0:0)	[M+Cl]-	C32H62O5	561.42913			3.8E+05	-1.5				
252	UDP-3-ketoglucose	[M-H]-	C15H22N2O17P2	563.03209					2.0E+06	-1.4		
253	PG(22:2(13Z,16Z)/0:0)	[M-H]-	C28H53O9P	563.33544					2.0E+06	1.1		
254	PA(12:0/14:0)	[M-H]-	C29H57O8P	563.37183					1.9E+06	-2.9		
255	2-Octaprenyl-3-methyl-6-methoxy-1,4-benzoquinol	[M-H]-	C38H60O3	563.44697					1.9E+06	-2.4		
256	DG(14:0/18:2(9Z,12Z)/0:0)	[M-H]-	C35H64O5	563.46810					2.6E+06	-0.6		
257	LysoPE(0:0/22:4(7Z,10Z,13Z,16Z))	[M+Cl]-	C27H48NO7P	564.28624					2.1E+06	0.7		

Table S2: Untargeted metabolic profiling of Goji Berries hydroalcoholic extracts by ESI(-) FT-ICR MS

#	Putative annotation[a]	Ion	Formula	m/z_{Theo}[b]	BL1[c]	ppm	BL2[c]	ppm	SL1[c]	ppm	SL2[c]	ppm
258	4,5-Dihydro-4-hydroxy-5-S-glutathionyl-benzo[a]pyrene	[M-H]-	C30H29N3O7S	574.16534	8.2E+05	2.4					3.1E+04	-0.8
259	Myricetin 3-(2'',3'',4''-triacetylxyloside)	[M-H]-	C26H24O15	575.10424	3.8E+05	-1.8						
260	Afrormosin 7-O-(6''-malonylglucoside)	[M+Cl]-	C26H26O13	581.10674			6.4E+05	-0.9				
261	SQMG(0:0/16:1(11Z))	[M+Cl]-	C25H45O11S	588.23766	6.1E+05	2.4						
262	UDP-2-acetamido-4-amino-2,4,6-trideoxyglucose	[M-H]-	C17H28N4O15P2	589.09536	4.6E+05	-1.0						
263	Osthenol-7-O-beta-D-gentiobioside	[M+Cl]-	C26H34O13	589.16934	3.8E+05	-2.6						
264	4-Keto-4'-hydroxyalloxanthin	[M-H]-	C40H50O4	593.36363					7.6E+05	-2.9		
265	Herbacetin 8-(2'',3'',4''-triacetylxyloside)	[M+Cl]-	C26H24O14	595.08601			4.0E+05	-3.0				
266	5,7,2',5'-Tetrahydroxyflavanone 7-O-rutinoside	[M-H]-	C27H32O15	595.16684			3.4E+05	-2.7				
267	1-(15-methyl-tridecanyl)-2-(8-[3]-ladderane-octanyl)-sn-glycerol	[M+Cl]-	C37H68O3	595.48625	3.3E+05	-0.3						
268	Trilobine	[M+Cl]-	C35H34N2O5	597.21617			3.8E+05	-2.1				
269	PE(12:0/14:1(9Z))	[M-H]-	C31H60NO8P	604.39838	4.2E+05	-2.0						
270	1D-myo-Inositol 1,3,4,5,6-pentakisphosphate	[M+Cl]-	C6H17O21P5	614.86444					2.5E+06	-2.3		
271	PS(12:0/12:0)	[M+Cl]-	C30H58NO10P	658.34924					1.5E+06	-1.2		
272	PA(12:0/20:2(11Z,14Z))	[M+Cl]-	C35H65O8P	679.41111					1.8E+06	2.6		
273	Abrusoside A	[M+Cl]-	C36H54O10	681.34110					3.2E+06	-2.5		
274	Chitin	[M-H]-	C28H49N3O16	682.30401					9.5E+05	-0.2		
275	Gomphrenin-V	[M-H]-	C34H34N2O16	725.18356	3.3E+05	1.0						
276	Anhydroicaritin 3,7-diglucoside	[M+Cl]-	C33H40O16	727.20104					6.9E+05	-2.0		
277	PA(14:0/22:6(4Z,7Z,10Z,13Z,16Z,19Z))	[M+Cl]-	C39H65O8P	727.41111	6.0E+05	2.8						
278	PG(12:0/21:0)	[M-H]-	C39H77O10P	735.51816					3.5E+06	1.6		
279	Bilirubin glucuronide	[M-H]-	C39H44N4O12	759.28830	1.1E+06	2.6						
280	PE(16:1(9Z)/22:6(4Z,7Z,10Z,13Z,16Z,19Z))	[M-H]-	C43H72NO8P	760.49228	7.6E+05	-1.5						
281	PS(P-18:0/17:2(9Z,12Z))	[M+Cl]-	C41H76NO9P	792.49517			3.1E+05	2.1				
282	PA(19:1(9Z)/22:4(7Z,10Z,13Z,16Z))	[M+Cl]-	C44H77O8P	799.50501					9.7E+05	-2.1		
283	PS(15:0/20:5(5Z,8Z,11Z,14Z,17Z))	[M+Cl]-	C41H70NO10P	802.44314					6.0E+05	-0.8		

Table S2: Untargeted metabolic profiling of Goji Berries hydroalcoholic extracts by ESI(-) FT-ICR MS

#	Putative annotation[a]	Ion	Formula	m/z_{Theo}[b]	BL1[c]	ppm	BL2[c]	ppm	SL1[c]	ppm	SL2[c]	ppm
284	2-Methylbut-2-enoyl-CoA	[M-H]-	C26H42N7O17P3S	848.14980					9.2E+05	-2.9		
285	PGP(16:1(9Z)/22:6(4Z,7Z,10Z,13Z,16Z,19Z))	[M-H]-	C44H74O13P2	871.45319					5.6E+05	-2.6		
286	Deltonin	[M-H]-	C45H72O17	883.46967					8.4E+05	-2.2		
287	PI(20:0/22:6(4Z,7Z,10Z,13Z,16Z,19Z))	[M-H]-	C51H87O13P	937.58115	5.4E+05	0.5						
288	TG(16:0/17:0/22:0)	[M+Cl]-	C58H112O6	939.81529	3.5E+05	-0.3						
289	TG(16:0/20:0/22:1(13Z))	[M+Cl]-	C61H116O6	979.84659					1.9E+06	1.4		

a MG Monoacylglycerol; DG Diacylglycerol; TG Triacylglycerol; GalCer Galactosylceramide; PA Phosphatidic acid; PC Phosphatidylcholine; PE Phosphatidylethanolamine; PG Glycerophospholipids; PI Phosphatidylinositol; PS Phosphatidylserine; SQMG Glycosylmonoacylglycerols; DHAP Dihydroxyacetone phosphate; DNP Dinitrophenyl

b Theoretical m/z

[c] BL1 and BL2 stand for Big Lifeberry harvested in August and October, respectively; SL1 and SL2 stand for Sweet Lifeberry harvested in August and October, respectively.

Table S3: Untargeted metabolic profiling of Goji Berries organic extracts by ESI(+) Ion Trap MS[a]

#	m/z	Putative Annotation[b]	Formula	Ion	BL1[c]	BL2[c]	SL1[c]	SL2[c]
1	129.9	Pyroglutamic acid	C5H7NO3	[M+H]+		1.9E+03		
2	131.9	Leucine	C6H13NO2	[M+H]+		6.6E+03		2.8E+04
3	132.9	Ornithine	C5H12N2O2	[M+H]+		2.0E+03		
4	138.9	Nicotinamide N-oxide	C6H6N2O2	[M+H]+		5.3E+03		1.3E+04
5	146.7	Lysine	C6H14N2O2	[M+H]+	1.3E+04			
6	148.8	Cinnamic acid	C9H8O2	[M+H]+		1.6E+03		
7	162.9	Nicotine	C10H14N2	[M+H]+		4.2E+03		
8	172.8	Glycylproline	C7H12N2O3	[M+H]+	1.7E+04	2.7E+03		9.6E+03
9	174.9	Arginine	C6H14N4O2	[M+H]+	1.3E+04	4.7E+03		
10	188.9	Monomethyl-arginine	C7H16N4O2	[M+H]+		2.7E+03		
11	202.8	Aldohexose	C6H12O6	[M+Na]+	1.9E+04	3.0E+03		1.2E+04
12	209.8	Butenyl-methyl-threonine	C9H17NO3	[M+Na]+		2.0E+03	1.2E+04	
13	211.0	Azelaic acid	C9H16O4	[M+Na]+		2.5E+03		
14	214.9	Methyl lauric acid	C13H26O2	[M+H]+		1.6E+03		
15	218.9	Aldohexose	C6H12O6	[M+K]+	1.7E+04	3.1E+03		
16	256.9	Palmitic acid	C16H32O2	[M+H]+			1.1E+04	
17	260.9	Apiole	C12H14O4	[M+K]+			2.1E+04	9.9E+03
18	276.9	Chrysanthetriol	C15H26O3	[M+H]+	2.4E+04			
19	278.9	Linolenic acid	C18H30O2	[M+H]+	2.1E+04			
20	284.1	Acrifoline	C16H23NO2	[M+Na]+				1.1E+04
21	292.9	Methyl palmitic acid	C17H34O2	[M+Na]+	1.8E+04			
22	300.9	Methyl-hexadecanedioic acid	C17H32O4	[M+H]+				1.4E+04
23	303.0	Linoleic acid	C18H32O2	[M+Na]+	2.1E+04			
24	305.0	Arachidonic acid	C20H32O2	[M+H]+	3.1E+04		2.8E+04	1.5E+04
25	321.0	Phytanol	C20H42O	[M+Na]+	1.7E+04			1.2E+04
26	337.0	Trimethoxyflavanone	C18H18O5	[M+Na]+			1.9E+04	

Table S3: Untargeted metabolic profiling of Goji Berries organic extracts by ESI(+) Ion Trap MS[a]

#	m/z	Putative Annotation[b]	Formula	Ion	BL1[c]	BL2[c]	SL1[c]	SL2[c]
27	339.0	Docosenoic acid	C22H42O2	[M+H]+				1.2E+04
28	343.0	Phaseollidin hydrate	C20H22O5	[M+H]+	2.2E+04			1.3E+04
29	349.0	Methyl-eicosanoic acid	C21H42O2	[M+Na]+	1.8E+04		3.2E+04	1.6E+04
30	353.0	Octadecanedioic acid	C18H34O4	[M+K]+	2.6E+04			1.7E+04
31	357.0	Docosatrienoic acid	C22H38O2	[M+Na]+		9.6E+03		
32	361.2	Docosenoic acid	C22H42O2	[M+Na]+				1.5E+04
33	365.0	Eicosanedioic acid	C20H38O4	[M+Na]+	5.0E+04	1.9E+04	6.2E+04	5.5E+04
34	367.1	Hydroxydocosahexaenoic acid	C22H32O3	[M+Na]+				1.4E+04
35	369.0	Dimethyl-docosanoic acid	C24H48O2	[M+H]+	2.3E+04			1.3E+04
36	371.0	Docosanedioic acid	C22H42O4	[M+H]+	2.7E+04	1.2E+04		1.5E+04
37	381.0	MG(0:0/18:0/0:0)	C21H42O4	[M+Na]+	2.4E+04	1.2E+04	2.4E+04	2.0E+04
38	385.1	Cholecalciferol	C27H44O	[M+H]+	2.7E+04	1.4E+04	2.1E+04	1.5E+04
39	393.1	Docosanedioic acid	C22H42O4	[M+Na]+	4.5E+04	1.3E+04	4.7E+04	3.1E+04
40	395.1	Hexacosenoic acid	C26H50O2	[M+H]+				1.2E+04
41	399.1	Methylvitamin D3	C28H46O	[M+H]+	2.0E+04			1.2E+04
42	407.0	Hexacosahexaenoic acid	C26H40O2	[M+Na]+				1.4E+04
43	409.1	Hydroxychalcone-glucoside	C21H22O7	[M+Na]+	3.0E+04	1.4E+04	3.6E+04	2.2E+04
44	413.1	MG(0:0/22:1/0:0)	C25H48O4	[M+H]+	5.6E+04	2.6E+04	6.3E+04	5.0E+04
45	415.1	Tetrahydroxy-trimethoxyflavone	C18H16O9	[M+K]+				1.2E+04
46	421.1	Methylvitamin D3	C28H46O	[M+Na]+			2.1E+04	1.9E+04
47	423.1	Octacosenoic acid	C28H54O2	[M+H]+				1.3E+04
48	425.2	Octacosanoic acid	C28H56O2	[M+H]+		9.4E+03		1.3E+04
49	428.2	Stearoylcarnitine	C25H49NO4	[M+H]+	2.9E+04	1.2E+04	2.1E+04	1.9E+04
50	429.1	Dihydroxynoroxavitamin D3	C25H42O4	[M+Na]+	4.2E+04	2.3E+04	4.4E+04	3.2E+04
51	431.2	Ginkgolide A	C20H24O9	[M+Na]+	1.8E+04			1.6E+04
52	433.1	Hexacosenoic acid	C26H50O2	[M+K]+	3.2E+04		4.2E+04	

Table S3: Untargeted metabolic profiling of Goji Berries organic extracts by ESI(+) Ion Trap MS[a]

#	m/z	Putative Annotation[b]	Formula	Ion	BL1[c]	BL2[c]	SL1[c]	SL2[c]
53	437.1	Tridecyl oleate	C29H56O2	[M+H]+	3.5E+04	1.1E+04	5.4E+04	2.6E+04
54	439.1	Beta-tocopherol	C28H48O2	[M+Na]+				1.2E+04
55	441.1	apo-Carotenol	C30H42O	[M+Na]+	2.2E+04	1.3E+04	2.7E+04	2.2E+04
56	443.1	Scillirosidin	C26H34O6	[M+H]+	1.9E+04		2.3E+04	1.6E+04
57	445.2	Dihydroxynoroxavitamin D3	C25H42O4	[M+K]+		1.1E+04	2.4E+04	1.4E+04
58	447.1	Alpha-Tocotrienol	C29H44O2	[M+Na]+			2.1E+04	1.3E+04
59	449.2	Hexacosanedioic acid	C26H50O4	[M+Na]+	2.9E+04	1.3E+04	2.2E+04	1.9E+04
60	451.1	Myristyl oleate	C30H58O2	[M+H]+			2.1E+04	
61	453.1	Alpha-Tocopherol	C29H50O2	[M+Na]+	2.2E+04	1.2E+04	4.1E+04	1.9E+04
62	455.1	Hydroxy-gama-tocopherol	C28H48O3	[M+Na]+			2.6E+04	1.2E+04
63	457.2	Trihydroxy-dihydrovitamin D3	C27H46O4	[M+Na]+		1.0E+04	2.7E+04	1.7E+04
64	459.2	Tetramethy-tetracosadienoic acid	C28H52O2	[M+K]+			2.0E+04	
65	465.1	MG(24:0/0:0/0:0)	C27H54O4	[M+Na]+	1.8E+04		2.5E+04	1.7E+04
66	467.1	Triacontatetraenoic acid	C30H52O2	[M+Na]+			2.4E+04	
67	469.1	Hydroxy-tocopherol	C29H50O3	[M+Na]+			2.0E+04	1.3E+04
68	471.1	Chlorogenin	C28H48O4	[M+Na]+			2.0E+04	
69	473.2	Trihydroxy-cholestanoic acid	C27H46O5	[M+Na]+	2.5E+04	1.5E+04	4.3E+04	3.1E+04
70	475.1	Dihydroxy-dimethylhexadehydro-homovitamin D3	C30H44O3	[M+Na]+			2.3E+04	1.5E+04
71	477.2	Dihydroxy-dimethyl-tetradehydro-homovitamin D3	C30H46O3	[M+Na]+			2.3E+04	
72	479.1	Palmityl oleate	C32H62O2	[M+H]+			2.6E+04	1.3E+04
73	481.1	Hexadecyl hexadecanoate	C32H64O2	[M+H]+	2.3E+04	1.4E+04	6.3E+04	2.2E+04
74	483.1	Myrrhanol A	C30H52O3	[M+Na]+			2.0E+04	
75	485.2	Dehydroecdysone	C27H42O6	[M+Na]+			2.0E+04	
76	487.1	Trihydroxycoprostanoic acid	C28H48O5	[M+Na]+	2.0E+04	1.1E+04	3.4E+04	1.6E+04
77	489.2	Tetrahydroxy-cholestanoic acid	C27H46O6	[M+Na]+	1.7E+04	1.4E+04	3.9E+04	1.7E+04
78	491.2	(Hydroxy-propynyl)-didehydrovitamin D3	C30H44O4	[M+Na]+			2.2E+04	

Table S3: Untargeted metabolic profiling of Goji Berries organic extracts by ESI(+) Ion Trap MS[a]

#	m/z	Putative Annotation[b]	Formula	Ion	BL1[c]	BL2[c]	SL1[c]	SL2[c]
79	493.2	(Carboxylpropyl)vitamin D3	C31H50O3	[M+Na]+	1.8E+04	9.5E+03	3.3E+04	1.6E+04
80	495.2	Dihydroxy-dimethyl-dihomovitamin D3	C31H52O3	[M+Na]+		1.0E+04	3.0E+04	1.4E+04
81	497.1	PG(17:1/0:0)	C23H45O9P	[M+H]+	1.8E+04	1.1E+04	4.7E+04	2.2E+04
82	501.2	Dihydroxy-(hydroxypropoxy)-norvitamin D3	C29H50O5	[M+Na]+	2.0E+04	1.1E+04	3.2E+04	1.5E+04
83	503.2	epi-Brassinolide	C28H48O6	[M+Na]+	2.0E+04	1.3E+04	2.9E+04	1.8E+04
84	505.2	Triacontanedioic acid	C30H58O4	[M+Na]+		1.1E+04	3.3E+04	1.5E+04
85	507.2	Stearyl oleate	C34H66O2	[M+H]+			2.1E+04	1.2E+04
86	509.1	Diethyl-dihydroxy-methano-oxavitamin D3	C31H50O4	[M+Na]+	2.0E+04	2.5E+04	5.1E+04	2.4E+04
87	511.1	PG(18:1(9Z)/0:0)	C24H47O9P	[M+H]+			2.3E+04	1.2E+04
88	513.2	Dihydroxy-(hydroxypropoxy)vitamin D3	C30H50O5	[M+Na]+			2.1E+04	1.2E+04
89	515.2	Dihydroxy-phenylvitamin D3	C33H48O3	[M+Na]+		1.1E+04	2.0E+04	1.1E+04
90	517.2	Makisterone A	C28H46O7	[M+Na]+	2.3E+04	1.6E+04	1.1E+05	3.1E+04
91	518.2	PC(O-16:0/O-1:0)	C25H54NO6P	[M+Na]+			4.1E+04	1.3E+04
92	519.2	epi-Brassinolide	C28H48O6	[M+K]+		9.9E+03	9.8E+04	2.1E+04
93	521.2	Tetratriacontapentaenoic acid	C34H58O2	[M+Na]+		1.0E+04	3.2E+04	1.8E+04
94	523.2	Tetratriacontatetraenoic acid	C34H60O2	[M+Na]+		1.2E+04	2.7E+04	1.2E+04
95	525.1	PG(19:1/0:0)	C25H49O9P	[M+H]+	1.8E+04	2.0E+04	5.2E+04	2.6E+04
96	527.2	PG(19:0/0:0)	C25H51O9P	[M+H]+			2.2E+04	
97	529.2	Hydroxy-norgeminivitamin D3	C31H54O5	[M+Na]+	1.8E+04	1.3E+04	2.2E+04	
98	531.2	Mucronine B	C28H36N4O4	[M+K]+	2.2E+04	1.8E+04	4.6E+04	2.8E+04
99	533.2	DG(14:1/14:0/0:0)	C31H58O5	[M+Na]+	2.2E+04	1.7E+04	4.4E+04	2.7E+04
100	535.2	Dimyristyl-sn-glycerol	C31H60O5	[M+Na]+		1.0E+04	2.8E+04	1.5E+04
101	536.2	PC(P-20:0/0:0)	C28H58NO6P	[M+H]+			4.7E+04	1.2E+04
102	537.2	Phthioceranic acid (C36)	C36H72O2	[M+H]+		1.1E+04	3.8E+04	1.5E+04
103	539.2	Tetratriacontatetraenoic acid	C34H60O2	[M+K]+			2.5E+04	
104	545.2	Oleoylglycerophosphocholine	C26H53NO7P	[M+Na]+	1.8E+04	1.3E+04	3.0E+04	1.6E+04

Table S3: Untargeted metabolic profiling of Goji Berries organic extracts by ESI(+) Ion Trap MS[a]

#	m/z	Putative Annotation[b]	Formula	Ion	BL1[c]	BL2[c]	SL1[c]	SL2[c]
105	547.2	DG(14:1/15:0/0:0)	C32H60O5	[M+Na]+	2.1E+04	2.0E+04	4.9E+04	2.7E+04
106	549.3	DG(14:0/15:0/0:0)	C32H62O5	[M+Na]+	1.8E+04	1.6E+04	4.1E+04	2.5E+04
107	551.2	(Tetradecenyl)-(octadecenoyl)-sn-glycerol	C35H66O4	[M+H]+		1.2E+04		1.3E+04
108	553.3	((O-hydroxy-ethane)-hydroxy-propane)- hopane	C35H62O3	[M+Na]+		1.0E+04	3.1E+04	1.7E+04
109	557.1	PG(20:3/0:0)	C26H47O9P	[M+Na]+	4.2E+04	2.2E+04	7.0E+04	3.1E+04
110	559.2	DG(14:1/16:1/0:0)	C33H60O5	[M+Na]+		9.9E+03	2.1E+04	1.4E+04
111	561.2	DG(14:0/16:1/0:0)	C33H62O5	[M+Na]+	2.3E+04	1.7E+04	4.7E+04	3.4E+04
112	563.2	DG(14:0/16:0/0:0)	C33H64O5	[M+Na]+		1.5E+04	3.8E+04	2.2E+04
113	565.2	(D-ribonyl)hopane	C35H58O4	[M+Na]+		1.0E+04	2.7E+04	1.3E+04
114	567.2	DG(14:0/18:1/0:0)	C35H66O5	[M+H]+			2.3E+04	1.1E+04
115	569.2	Cholest-dienol O-beta-D-glucopyranoside	C33H54O6	[M+Na]+	2.8E+04	1.8E+04	7.5E+04	2.8E+04
116	571.2	Hexaprenyl-methoxy-benzoquinol	C37H56O3	[M+Na]+		1.1E+04	2.1E+04	1.2E+04
117	575.2	DG(15:0/16:1/0:0)	C34H64O5	[M+Na]+	1.8E+04	1.4E+04	3.8E+04	2.3E+04
118	577.2	DG(15:0/16:0/0:0)	C34H66O5	[M+Na]+	2.1E+04	1.6E+04	4.4E+04	3.0E+04
119	579.2	PA(O-16:0/12:0)	C31H63O7P	[M+H]+		9.4E+03	2.9E+04	1.7E+04
120	585.2	DG(14:0/18:3/0:0)	C35H62O5	[M+Na]+		1.2E+04	3.7E+04	1.6E+04
121	587.3	DG(14:0/18:2/0:0)	C35H64O5	[M+Na]+			2.3E+04	1.3E+04
122	591.2	DG(14:0/18:0/0:0)	C35H68O5	[M+Na]+		1.3E+04	3.8E+04	2.3E+04
123	593.2	PA(O-16:0/13:0)	C32H65O7P	[M+H]+			2.4E+04	1.5E+04
124	601.2	DG(15:0/18:2/0:0)	C36H66O5	[M+Na]+			2.5E+04	1.2E+04
125	603.3	DG(15:0/18:1/0:0)	C36H68O5	[M+Na]+			2.5E+04	1.4E+04
126	605.3	DG(15:0/18:0/0:0)	C36H70O5	[M+Na]+		1.3E+04	3.7E+04	2.2E+04
127	607.2	PA(O-16:0/14:0)	C33H67O7P	[M+H]+			2.0E+04	1.2E+04
128	609.3	5-Oxoavermectin "2b" aglycone	C33H46O9	[M+Na]+			2.0E+04	
129	611.3	DG(14:0/20:4/0:0)	C37H64O5	[M+Na]+			2.0E+04	
130	613.2	DG(14:0/20:3/0:0)	C37H66O5	[M+Na]+			2.1E+04	

Table S3: Untargeted metabolic profiling of Goji Berries organic extracts by ESI(+) Ion Trap MS[a]

#	m/z	Putative Annotation[b]	Formula	Ion	BL1[c]	BL2[c]	SL1[c]	SL2[c]
131	615.3	DG(14:0/20:2/0:0)	C37H68O5	[M+Na]+			4.4E+04	1.3E+04
132	617.3	Hexadecanoyl-(octadecenoyl)-sn-glycerol	C37H70O5	[M+Na]+			2.8E+04	1.4E+04
133	619.2	PA(O-16:0/15:1)	C34H67O7P	[M+H]+			3.3E+04	1.2E+04
134	621.3	PA(O-16:0/15:0)	C34H69O7P	[M+H]+			2.9E+04	1.4E+04
135	631.3	PA(P-16:0/16:1)	C35H67O7P	[M+H]+			2.4E+04	
136	633.3	PA(O-16:0/16:1)	C35H69O7P	[M+H]+			2.8E+04	
137	635.3	PA(O-16:0/16:0)	C35H71O7P	[M+H]+			2.6E+04	
138	637.3	DG(14:0/22:5/0:0)	C39H66O5	[M+Na]+			2.4E+04	
139	639.3	DG(14:0/22:4/0:0)	C39H68O5	[M+Na]+			2.2E+04	
140	641.3	DG(14:1/22:2/0:0)	C39H70O5	[M+Na]+			1.9E+04	
141	643.0	DG(14:0/22:2/0:0)	C39H72O5	[M+Na]+			1.9E+04	
142	649.3	PA(O-16:0/17:0)	C36H73O7P	[M+H]+			3.0E+04	
143	651.2	DG(15:0/22:5/0:0)	C40H68O5	[M+Na]+			2.0E+04	
144	663.2	PA(16:0e/18:0)	C37H75O7P	[M+H]+			3.7E+04	
145	665.2	DG(16:0/22:5/0:0)	C41H70O5	[M+Na]+			3.0E+04	
146	677.3	PA(O-16:0/19:0)	C38H77O7P	[M+H]+			2.2E+04	
147	679.2	PG(12:0/17:1)	C35H67O10P	[M+H]+			2.2E+04	
148	685.2	PA(O-16:0/20:3)	C39H73O7P	[M+H]+			4.3E+04	
149	691.2	PG(P-16:0/15:1)	C37H71O9P	[M+H]+			2.1E+04	
150	693.3	PG(12:0/18:1)	C36H69O10P	[M+H]+			3.3E+04	
151	695.3	PG(12:0/18:0)	C36H71O10P	[M+H]+			1.9E+04	
152	707.3	PG(12:0/19:1)	C37H71O10P	[M+H]+			2.1E+04	

a Experiments carried out by ESI(+) Esquire 6000 MS

[b] MG Monoacylglycerol; DG Diacylglycerol; TG Triacylglycerol; GalCer Galactosylceramide; PA Phosphatidic acid; PC Phosphatidylcholine; PE Phosphatidylethanolamine; PG Glycerophospholipids; PI Phosphatidylinositol;

Table S3: Untargeted metabolic profiling of Goji Berries organic extracts by ESI(+) Ion Trap MS[a]

#	m/z	Putative Annotation[b]	Formula	Ion	BL1[c]	BL2[c]	SL1[c]	SL2[c]

[c] BL1 and BL2 stand for Big Lifeberry harvested in August and October, respectively; SL1 and SL2 stand for Sweet Lifeberry harvested in August and October, respectively.

Table S4: Untargeted metabolic profiling of Goji Berries organic extracts by ESI(-) Ion Trap MS[a]

#	m/z	Putative Annotation[a]	Formula	Ion	BL1[b]	BL2[b]	SL1[b]	SL2[b]
1	110.8	Hexenedial	C6H8O2	[M-H]-			1.9E+03	
2	124.9	Octenal	C8H14O	[M-H]-	1.4E+03			
3	128.1	Pyroglutamic acid	C5H7NO	[M-H]-	4.6E+03	1.4E+03	3.4E+03	5.5E+03
4	136.9	Hexan-1-ol	C6H14O	[M+Cl]-	1.6E+03			
5	146.9	Hexenedial	C6H8O2	[M+Cl]-	1.7E+03		3.6E+03	
6	156.9	Methyl-octanoic acid	C9H18O2	[M-H]-	1.6E+03			
7	158.8	Octatrienal	C8H10O	[M+Cl]-			7.7E+03	2.6E+03
8	160.9	Octenal	C8H14O	[M+Cl]-			7.1E+03	2.3E+03
9	169.7	Decanamide	C10H21NO	[M-H]-			4.5E+03	
10	171.0	Limonene	C10H16	[M+Cl]-	1.5E+03			3.7E+03
11	172.9	Suberic acid	C8H14O4	[M-H]-			2.1E+03	
12	179.0	Octenoic acid	C8H14O2	[M+Cl]-	1.3E+03			2.3E+03
13	186.0	Hydroxyadenine	C5H5N5O	[M+Cl]-	1.4E+03		2.3E+03	2.4E+03
14	187.0	Decadienal	C10H16O	[M+Cl]-	1.7E+03		3.7E+03	
15	199.0	Decenedioic acid	C10H16O4	[M-H]-	2.3E+03	9.0E+02	2.5E+03	5.1E+03
16	201.0	Decanedioic acid	C10H18O4	[M-H]-	2.3E+03			
17	213.1	Methyl-dodecanoic acid	C13H26O2	[M-H]-				2.8E+03
18	225.1	Tuberonic acid	C12H18O4	[M-H]-	1.7E+03			3.6E+03
19	227.1	Myristic acid	C14H28O2	[M-H]-	2.2E+04	2.4E+04	2.1E+04	6.6E+04
20	239.1	Tridecenyl acetate	C15H28O2	[M-H]-	2.2E+03	1.2E+03	2.0E+03	5.7E+03
21	241.1	Methyl-myristic acid	C15H30O2	[M-H]-	1.0E+04	5.5E+03	9.1E+03	3.1E+04
22	253.1	Palmitoleic acid	C16H30O2	[M-H]-	2.2E+04	1.2E+04	1.6E+04	6.2E+04
23	255.2	Palmitic acid	C16H32O2	[M-H]-	4.9E+04	2.4E+04	3.9E+04	2.1E+05
24	265.0	Methyl-Hexadecadienoic acid	C17H30O2	[M-H]-	3.9E+04	2.1E+04	5.6E+04	6.5E+04
25	267.1	Methyl-palmitoleic acid	C17H32O2	[M-H]-	8.8E+03	4.4E+03	7.2E+03	2.2E+04
26	269.1	Methyl-palmitic acid	C17H34O2	[M-H]-	9.1E+03	4.4E+03	6.8E+03	3.3E+04

Table S4: Untargeted metabolic profiling of Goji Berries organic extracts by ESI(-) Ion Trap MS[a]

#	m/z	Putative Annotation[a]	Formula	Ion	BL1[b]	BL2[b]	SL1[b]	SL2[b]
27	271.0	Hexadecadienal	C16H28O	[M+Cl]-				2.7E+03
28	277.1	Linolenic acid	C18H30O2	[M-H]	1.5E+03		2.1E+03	2.4E+03
29	279.1	Linoleic acid	C18H32O2	[M-H]-	1.1E+04	3.1E+03	6.6E+03	2.4E+04
30	281.1	Oleic acid	C18H34O2	[M-H]-	3.1E+04	1.2E+04	1.8E+04	1.0E+05
31	283.1	Stearic acid	C18H36O2	[M-H]-	3.6E+04	1.1E+04	1.9E+04	1.1E+05
32	293.1	Methyl lineoleate	C19H34O2	[M-H]-	2.1E+04	7.9E+03	2.4E+04	3.7E+04
33	295.1	Methyl oleate	C19H36O2	[M-H]-	3.6E+03	1.4E+03	2.9E+03	7.1E+03
34	297.0	Methyl stearate	C19H38O2	[M-H]-	6.9E+03	2.5E+03	9.4E+03	1.4E+04
35	299.0	Hydroxystearic acid	C18H36O3	[M-H]-	1.7E+03		1.9E+03	3.7E+03
36	305.1	Linolenic acid, ethyl ester	C20H34O2	[M-H]-	1.3E+03			2.9E+03
37	307.0	Eicosadienoic acid	C20H36O2	[M-H]-	8.1E+03	2.3E+03	5.8E+03	1.2E+04
38	309.0	Eicosenoic acid	C20H38O2	[M-H]-	2.8E+04	9.2E+03	2.3E+04	4.6E+04
39	311.0	Arachidic acid	C20H40O2	[M-H]-	2.4E+04	8.1E+03	3.5E+04	4.6E+04
40	313.0	Dihydroxy-octadecenoic acid	C18H34O4	[M-H]-	3.1E+03	1.1E+03	3.8E+03	5.9E+03
41	315.0	Dihydroxy-octadecanoic acid	C18H36O4	[M-H]-	1.9E+03		2.6E+03	3.4E+03
42	321.0	Eicosadienoic acid, methyl ester	C21H38O2	[M-H]-	2.6E+03	1.0E+03	3.0E+03	4.9E+03
43	323.0	Eicosenoic acid, methyl ester	C21H40O2	[M-H]-	2.2E+03	1.1E+03	2.7E+03	3.9E+03
44	325.0	Methyl arachidate	C21H42O2	[M-H]-	2.2E+04	8.4E+03	4.0E+04	4.4E+04
45	327.0	Hydroxy-eicosanoic acid	C20H40O3	[M-H]-	2.8E+03		4.2E+03	5.2E+03
46	329.1	MG(0:0/16:0/0:0)	C19H38O4	[M-H]-	1.8E+03			2.6E+03
47	335.1	Docosadienoic acid	C22H40O2	[M-H]-	1.9E+03			3.6E+03
48	337.0	Docosenoic acid (22:1)	C22H42O2	[M-H]-	9.6E+03	2.8E+03	9.3E+03	1.9E+04
49	339.0	Docosanoic acid (22:0)	C22H44O2	[M-H]-	1.7E+04	6.1E+03	3.0E+04	4.0E+04
50	341.0	Eicosanedioic acid	C20H38O4	[M-H]-	4.5E+03	2.1E+03	5.6E+03	8.6E+03
51	343.0	Dihydroxy-methoxy-octadecenoic acid	C19H36O5	[M-H]-	1.9E+03		2.0E+03	2.9E+03
52	351.1	Docosenoic acid methyl ester	C23H44O2	[M-H]-				2.3E+03

Table S4: Untargeted metabolic profiling of Goji Berries organic extracts by ESI(-) Ion Trap MS[a]

#	m/z	Putative Annotation[a]	Formula	Ion	BL1[b]	BL2[b]	SL1[b]	SL2[b]
53	353.0	Docosanoic acid, methyl ester	C23H46O2	[M-H]-	1.8E+04	5.7E+03	1.5E+04	3.1E+04
54	355.0	Hydroxy-docosanoic acid	C22H44O3	[M-H]-	2.6E+03	9.4E+02	2.8E+03	4.2E+03
55	363.0	Tetracosadienoic acid	C24H44O2	[M-H]-	1.8E+03		2.0E+03	3.1E+03
56	365.1	Tetracosenoic acid (24:1)	C24H46O2	[M-H]-	1.7E+03			3.7E+03
57	367.2	Tetracosanoic acid (24:0)	C24H48O2	[M-H]-	4.5E+03	1.6E+03	3.0E+03	1.3E+04
58	369.1	Docosanedioic acid	C22H42O4	[M-H]-	2.0E+03			3.7E+03
59	371.1	Deoxyoleandolide	C20H36O6	[M-H]-				2.3E+03
60	378.9	Tetracosenoic acid, methyl ester	C25H48O2	[M-H]-	1.9E+03		2.8E+03	3.2E+03
61	381.1	Hydroxy-tetracosenoic acid	C24H46O3	[M-H]-	6.8E+03	2.2E+03	6.9E+03	1.6E+04
62	383.1	Methyl-tetracosanoic acid	C25H50O2	[M-H]-	2.2E+03		2.7E+03	5.2E+03
63	391.1	Hexacosadienoic acid	C26H48O2	[M-H]-	1.3E+03			2.8E+03
64	393.1	Hexacosenoic acid	C26H50O2	[M-H]-				2.6E+03
65	395.2	Methyl-hexacosanoic acid	C26H52O2	[M-H]-	2.3E+03		2.4E+03	7.5E+03
66	397.1	Tetracosanedioic acid	C24H46O4	[M-H]-	9.5E+03	3.1E+03	9.3E+03	1.9E+04
67	399.1	Hydroxyvitamin D3	C27H44O2	[M-H]-	1.6E+03			2.9E+03
68	409.2	Gama-Tocotrienol	C28H42O2	[M-H]-				2.7E+03
69	411.2	Hydroxyhexacosanoic acid	C26H52O3	[M-H]-	1.5E+03			4.2E+03
70	416.9	Phyllanthin	C24H34O6	[M-H]-			2.0E+03	
71	421.1	Octacosenoic acid	C28H54O2	[M-H]-				2.7E+03
72	423.2	Montanic acid	C28H56O2	[M-H]-			1.9E+03	4.4E+03
73	425.1	Hexacosanedioic acid	C26H50O4	[M-H]-	3.9E+03	1.3E+03	5.0E+03	8.3E+03
74	431.0	Calcitetrol	C27H44O4	[M-H]-	2.1E+03		5.4E+03	3.9E+03
75	433.1	Trihydroxy-dihydrovitamin D3	C27H46O4	[M-H]-				2.7E+03
76	439.2	MG(24:1/0:0/0:0)	C27H52O4	[M-H]-				3.0E+03
77	441.1	Methyl-hexacosadienoic acid	C27H50O2	[M+Cl]-	4.4E+03	1.8E+03	5.3E+03	9.6E+03
78	443.1	Geranylgeranyl-glycerol phosphate	C23H41O6P	[M-H]-				2.4E+03

Table S4: Untargeted metabolic profiling of Goji Berries organic extracts by ESI(-) Ion Trap MS[a]

#	m/z	Putative Annotation[a]	Formula	Ion	BL1[b]	BL2[b]	SL1[b]	SL2[b]
79	449.1	Myristyl oleate	C30H58O2	[M-H]-				2.7E+03
80	451.2	Melissic acid	C30H60O2	[M-H]-				2.7E+03
81	465.2	Melissic acid methyl ester	C31H62O2	[M-H]-	2.4E+03	1.1E+03	5.8E+03	9.0E+03
82	467.2	Hydroxy-triacontanoic acid	C30H60O3	[M-H]-				2.9E+03
83	469.2	Methyl-octacosadienoic acid	C29H54O2	[M+Cl]-	1.6E+03		3.0E+03	4.7E+03
84	477.2	Palmityl oleate	C32H62O2	[M-H]-				2.3E+03
85	481.2	Triacontanedioic acid	C30H58O4	[M-H]-				3.0E+03
86	483.2	Triacontenoic acid (30:2)	C30H56O2	[M+Cl]-				2.8E+03
87	485.2	Triacontenoic acid (30:1)	C30H58O2	[M+Cl]-	9.0E+04	2.4E+05	1.8E+05	2.6E+05
88	487.2	Fucosyllactose	C18H32O15	[M-H]-	4.8E+03	1.2E+04	9.8E+03	1.4E+04
89	511.1	DG(14:0/14:0/0:0)	C31H60O5	[M-H]-				2.4E+03
90	573.3	DG(16:1/14:0/0:0)	C33H62O5	[M+Cl]-			2.2E+03	2.5E+03
91	601.3	DG(16:0/16:1/0:0)	C35H66O5	[M+Cl]-			2.3E+03	
92	615.1	Dimethyl-pentadecanoyl-(ladderaneoctanyl)-sn- glycerol	C40H72O4	[M-H]-			2.4E+03	3.3E+03
93	617.1	DG(15:0/18:0/0:0)	C36H70O5	[M+Cl]-			2.0E+03	2.4E+03
94	671.3	PA(16:0/18:2)	C37H69O8P	[M-H]-		1.1E+03	3.8E+03	4.4E+03
95	695.3	PA(18:1/18:3)	C39H69O8P	[M-H]-		9.5E+02	2.3E+03	3.8E+03

a Experiments carried out by ESI(-) Esquire 6000 MS

b MG Monoacylglycerol; DG Diacylglycerol; TG Triacylglycerol; GalCer Galactosylceramide; PA Phosphatidic acid; PC Phosphatidylcholine; PE Phosphatidylethanolamine; PG Glycerophospholipids; PI Phosphatidylinositol;

c BL1 and BL2 stand for Big Lifeberry harvested in August and October, respectively; SL1 and SL2 stand for Sweet Lifeberry harvested in August and October, respectively.

Table S5: Untargeted metabolic profiling of Goji Berries leaves hydroalcoholic extracts by ESI(+) FT-ICR MS

#	Plausible compound[a]	Ion	Molecular formula (M)	Theo. m/z[b]	Exp. m/z[c]	ppm	Intensity
1	N,N'-Dimethylurea	[M+H]+	C3H8N2O	89.07080	89.07094	-1.6	1.2E+05
2	Glyoxylic acid	[M+Na]+	C2H2O3	96.98941	96.98961	-2.1	1.7E+05
3	Trimethylamine N-oxide	[M+Na]+	C3H9NO	98.05752	98.05763	-1.2	1.4E+05
4	Pentadienoic acid	[M+H]+	C5H6O2	99.04426	99.04406	2.1	1.3E+05
5	Glutaraldehyde	[M+H]+	C5H8O2	101.05974	101.05971	0.3	1.1E+05
6	Aminopentanal	[M+H]+	C5H11NO	102.09127	102.09134	-0.7	1.2E+05
7	Serine	[M+H]+	C3H7NO3	106.04999	106.04987	1.1	1.5E+05
8	Diethanolamine	[M+H]+	C4H11NO2	106.08629	106.08626	0.3	2.5E+05
9	Propynoic acid	[M+K]+	C3H2O2	108.96837	108.96864	-2.5	1.2E+05
10	Methyl sulfate	[M+H]+	CH4O4S	112.99049	112.99031	1.6	1.5E+05
11	Thioacetic acid	[M+K]+	C2H4OS	114.96164	114.96145	1.7	1.4E+05
12	Glycerol	[M+Na]+	C3H8O3	115.03687	115.03656	2.7	1.7E+05
13	Cysteamine	[M+K]+	C2H7NS	115.99285	115.99308	-2.0	1.0E+05
14	Cumene	[M+H]+	C9H12	121.10121	121.10118	0.3	1.5E+05
15	Octatrienal	[M+H]+	C8H10O	123.08018	123.08044	-2.1	1.3E+05
16	Octadienal	[M+H]+	C8H12O	125.09591	125.09609	-1.5	1.3E+05
17	Putrescine	[M+K]+	C4H12N2	127.06322	127.06321	0.1	2.2E+05
18	Melamine	[M+H]+	C3H6N6	127.07298	127.07267	2.4	3.9E+05
19	Pyroglutamic acid	[M+H]+	C5H7NO3	130.04959	130.04987	-2.1	1.5E+05
20	Aniline	[M+K]+	C6H7N	132.02080	132.02101	-1.6	1.2E+05
21	Leucine	[M+H]+	C6H13NO2	132.10215	132.10191	1.9	1.0E+05
22	Pyridinol	[M+K]+	C5H5NO	133.99999	134.00027	-2.1	2.7E+05
23	Ureidoglycine	[M+H]+	C3H7N3O3	134.05584	134.05602	-1.3	3.4E+05
24	Cinnamyl alcohol	[M+H]+	C9H10O	135.08067	135.08044	1.7	1.3E+05
25	Adenine	[M+H]+	C5H5N5	136.06148	136.06177	-2.1	2.1E+05
26	Caprolactam	[M+Na]+	C6H11NO	136.07293	136.07328	-2.6	1.7E+05

Table S5: Untargeted metabolic profiling of Goji Berries leaves hydroalcoholic extracts by ESI(+) FT-ICR MS

#	Plausible compound[a]	Ion	Molecular formula (M)	Theo. m/z[b]	Exp. m/z[c]	ppm	Intensity
27	Hypoxanthine	[M+H]+	C5H4N4O	137.04612	137.04579	2.4	1.4E+05
28	Nonatrienal	[M+H]+	C9H12O	137.09576	137.09609	-2.4	1.5E+05
29	Levulinic acid	[M+Na]+	C5H8O3	139.03624	139.03656	-2.3	3.8E+05
30	Valine	[M+Na]+	C5H11NO2	140.06814	140.06820	-0.4	2.8E+05
31	Succinic acid	[M+Na]+	C4H6O4	141.01613	141.01583	2.1	1.3E+05
32	Allophanic acid	[M+K]+	C2H4N2O3	142.98508	142.98535	-1.9	1.3E+05
33	Cyanopyridine	[M+K]+	C6H4N2	143.00042	143.00061	-1.3	1.5E+05
34	Mercaptolactate	[M+Na]+	C3H6O3S	144.99304	144.99299	0.4	5.1E+05
35	Erythritol	[M+Na]+	C4H10O4	145.04670	145.04713	-3.0	3.8E+05
36	Keto-glutaramic acid	[M+H]+	C5H7NO4	146.04512	146.04478	2.3	2.5E+05
37	Triethanolamine	[M+H]+	C6H15NO3	150.11248	150.11247	0.1	3.5E+05
38	Thiodiacetic acid	[M+H]+	C4H6O4S	151.00574	151.00596	-1.4	2.5E+05
39	Cyclohexane-1,3-dione	[M+K]+	C6H8O2	151.01578	151.01559	1.3	1.6E+05
40	Pimelic dialdehyde	[M+Na]+	C7H12O2	151.07253	151.07295	-2.8	1.9E+05
41	Phenylpropanolamine	[M+H]+	C9H13NO	152.10709	152.10699	0.7	1.5E+05
42	Thiopurine	[M+H]+	C5H4N4S	153.02332	153.02294	2.5	1.6E+05
43	Isopropylcatechol	[M+H]+	C9H12O2	153.09143	153.09101	2.8	1.4E+05
44	Geranial	[M+H]+	C10H16O	153.12717	153.12739	-1.4	1.4E+05
45	Hydroxyproline	[M+Na]+	C5H9NO3	154.04766	154.04746	1.3	1.3E+05
46	Vanillylamine	[M+H]+	C8H11NO2	154.08665	154.08626	2.6	1.6E+05
47	Glutarate	[M+Na]+	C5H8O4	155.03185	155.03148	2.4	2.7E+05
48	Hydroxyhexanoic acid	[M+Na]+	C6H12O3	155.06816	155.06786	1.9	2.8E+05
49	S-Dimethylsulfonium propionic acid	[M+Na]+	C5H10O2S	157.02977	157.02937	2.5	3.0E+05
50	Homostachydrine	[M+H]+	C8H15NO2	158.11779	158.11756	1.5	1.0E+05
51	Isopropylmaleate	[M+H]+	C7H10O4	159.06513	159.06519	-0.3	2.3E+05
52	Trigonelline	[M+Na]+	C7H7NO2	160.03670	160.03690	-1.2	1.2E+05

Table S5: Untargeted metabolic profiling of Goji Berries leaves hydroalcoholic extracts by ESI(+) FT-ICR MS

#	Plausible compound[a]	Ion	Molecular formula (M)	Theo. m/z[b]	Exp. m/z[c]	ppm	Intensity
53	Phenylethylamine	[M+K]+	C8H11N	160.05271	160.05231	2.5	1.2E+05
54	Urocanic acid	[M+Na]+	C6H6N2O2	161.03236	161.03215	1.3	1.8E+05
55	Umbelliferone	[M+H]+	C9H6O3	163.03897	163.03897	0.0	1.1E+06
56	Nicotine	[M+H]+	C10H14N2	163.12297	163.12297	0.0	1.0E+06
57	Ciliatine	[M+K]+	C2H8NO3P	163.98778	163.98734	2.7	4.5E+05
58	Amino-imidazolecarboxylic acid	[M+K]+	C4H5N3O2	166.00089	166.00134	-2.7	2.3E+05
59	Coniine	[M+K]+	C8H17N	166.09945	166.09926	1.2	3.0E+05
60	Aceto valeric acid	[M+Na]+	C7H12O3	167.06795	167.06786	0.5	3.0E+05
61	Acetamidobutanoate	[M+Na]+	C6H11NO3	168.06289	168.06311	-1.3	1.2E+05
62	Mevaldic acid	[M+Na]+	C6H10O4	169.04711	169.04713	-0.1	1.7E+06
63	Oxaluric acid	[M+K]+	C3H4N2O4	170.98021	170.98027	-0.3	1.6E+05
64	Tetrahydroisoquinoline	[M+K]+	C9H11N	172.05190	172.05231	-2.4	1.6E+05
65	Arginine	[M+H]+	C6H14N4O2	175.11909	175.11895	0.8	1.3E+05
66	N-Guanylhistamine	[M+Na]+	C6H11N5	176.09104	176.09067	2.1	1.4E+05
67	Propylmalate	[M+H]+	C7H12O5	177.07589	177.07575	0.8	1.2E+05
68	Hexose-lactone	[M+H]+	C6H10O6	179.05482	179.05501	-1.1	1.7E+05
69	Oxo-octenoic acid	[M+Na]+	C8H12O3	179.06770	179.06786	-0.9	2.2E+05
70	Hydroxynicotine	[M+H]+	C10H14N2O	179.11819	179.11789	1.7	2.0E+05
71	Keto-n-caprylic acid	[M+Na]+	C8H14O3	181.08399	181.08352	2.6	4.4E+05
72	Tert-Butyl-methylcatechol	[M+H]+	C11H16O2	181.12234	181.12231	0.2	2.1E+05
73	Pimelic acid	[M+Na]+	C7H12O4	183.06294	183.06278	0.9	1.6E+06
74	Norcotinine	[M+Na]+	C9H10N2O	185.06882	185.06853	1.5	2.3E+05
75	Acetylcysteine	[M+Na]+	C5H9NO3S	186.01915	186.01953	-2.1	1.4E+05
76	Triethanolamine	[M+K]+	C6H15NO3	188.06860	188.06835	1.3	1.5E+05
77	Diethylaniline	[M+K]+	C10H15N	188.08396	188.08361	1.9	1.4E+05
78	Guanine	[M+K]+	C5H5N5O	190.01226	190.01257	-1.6	9.9E+04

Table S5: Untargeted metabolic profiling of Goji Berries leaves hydroalcoholic extracts by ESI(+) FT-ICR MS

#	Plausible compound[a]	Ion	Molecular formula (M)	Theo. m/z[b]	Exp. m/z[c]	ppm	Intensity
79	Diaminosalicylic acid	[M+Na]+	C7H8N2O3	191.04249	191.04271	-1.2	2.1E+05
80	Geranic acid	[M+Na]+	C10H16O2	191.10422	191.10425	-0.2	1.8E+06
81	Dimethylaminoethylphosphonate	[M+K]+	C4H12NO3P	192.01814	192.01864	-2.6	1.1E+05
82	Citronellic acid	[M+Na]+	C10H18O2	193.12041	193.11990	2.6	3.4E+05
83	Aminopropanol O-phosphate	[M+K]+	C3H10NO4P	193.99793	193.99790	0.1	8.9E+04
84	Ferulic acid	[M+H]+	C10H10O4	195.06470	195.06519	-2.5	3.5E+05
85	Methyl-oxo-octanoic acid	[M+Na]+	C9H16O3	195.09883	195.09917	-1.7	4.4E+05
86	Dihydroxy-octanoic acid	[M+Na]+	C8H16O4	199.09410	199.09408	0.1	4.4E+05
87	Ricinine	[M+K]+	C8H8N2O2	203.02223	203.02174	2.4	4.4E+05
88	Aminophenylalanine	[M+Na]+	C9H12N2O2	203.07854	203.07910	-2.7	3.0E+05
89	Patchoula-diene	[M+H]+	C15H22	203.17948	203.17943	0.3	1.6E+05
90	Iridotrial	[M+Na]+	C10H14O3	205.08311	205.08352	-2.0	2.2E+05
91	Isocaryophyllene	[M+H]+	C15H24	205.19459	205.19508	-2.4	1.3E+05
92	Vanillic acid	[M+K]+	C8H8O4	207.00601	207.00542	2.9	1.1E+05
93	Oxo-decenoic acid	[M+Na]+	C10H16O3	207.09873	207.09917	-2.1	9.4E+05
94	Trimethyluric acid	[M+H]+	C8H10N4O3	211.08243	211.08257	-0.6	6.4E+05
95	N'-Nitrosonornicotine	[M+K]+	C9H11N3O	216.05312	216.05337	-1.2	8.8E+04
96	Hydroxynicotine	[M+K]+	C10H14N2O	217.07391	217.07377	0.6	1.1E+06
97	Dicyclohexylamine	[M+K]+	C12H23N	220.14588	220.14621	-1.5	8.6E+04
98	Hydroxytryptophan	[M+H]+	C11H12N2O3	221.09208	221.09207	0.1	1.5E+05
99	Linderic acid	[M+Na]+	C12H22O2	221.15179	221.15120	2.7	2.4E+05
100	Decenedioic acid	[M+Na]+	C10H16O4	223.09408	223.09408	0.0	1.2E+06
101	Pelargonyl acetic acid	[M+Na]+	C11H20O3	223.13066	223.13047	0.9	3.7E+05
102	Sebacic acid	[M+Na]+	C10H18O4	225.10940	225.10973	-1.5	1.5E+06
103	Dodecatriynoic acid	[M+K]+	C12H12O2	227.04641	227.04689	-2.1	5.1E+05
104	Hydroxycotinine	[M+K]+	C10H12N2O2	231.05236	231.05304	-2.9	4.3E+05

Table S5: Untargeted metabolic profiling of Goji Berries leaves hydroalcoholic extracts by ESI(+) FT-ICR MS

#	Plausible compound[a]	Ion	Molecular formula (M)	Theo. m/z[b]	Exp. m/z[c]	ppm	Intensity
105	Ammodendrine	[M+Na]+	C12H20N2O	231.14676	231.14678	-0.1	4.7E+05
106	Tetradecadienol	[M+Na]+	C14H26O	233.18786	233.18759	1.2	1.1E+05
107	Acetoxychavicol acetate	[M+H]+	C13H14O4	235.09689	235.09649	1.7	1.6E+05
108	Cucurbic acid	[M+Na]+	C12H20O3	235.13007	235.13047	-1.7	3.3E+05
109	Hexadecatrienal	[M+H]+	C16H26O	235.20573	235.20564	0.4	2.6E+05
110	Tridecanal	[M+K]+	C13H26O	237.16117	237.16152	-1.5	3.4E+05
111	Succinyl proline	[M+Na]+	C9H13NO5	238.06843	238.06859	-0.7	2.0E+05
112	N-Acetylserotonin	[M+Na]+	C12H14N2O2	241.09480	241.09475	0.2	1.7E+05
113	Isocaryophyllene	[M+K]+	C15H24	243.15117	243.15096	0.9	2.3E+05
114	Hexadecatetraenoic acid	[M+H]+	C16H24O2	249.18455	249.18491	-1.4	3.4E+05
115	Dihydroxyphenylglycol O-sulfate	[M+H]+	C8H10O7S	251.02213	251.02200	0.5	3.8E+05
116	Tyramine-O-sulfate	[M+K]+	C8H11NO4S	256.00338	256.00404	-2.6	1.0E+05
117	Palmitic amide	[M+H]+	C16H33NO	256.26287	256.26349	-2.4	8.8E+05
118	Jasminine	[M+K]+	C11H12N2O3	259.04760	259.04795	-1.4	4.4E+05
119	Theacrine	[M+K]+	C9H12N4O3	263.05359	263.05410	-1.9	1.3E+05
120	Tetradecadienoic acid	[M+K]+	C14H24O2	263.14119	263.14079	1.5	1.4E+05
121	Acutifolane A	[M+H]+	C16H22O3	263.16411	263.16417	-0.2	2.4E+05
122	Aminodeoxychorismate	[M+K]+	C10H11NO5	264.02735	264.02688	1.8	1.2E+05
123	Genipin	[M+K]+	C11H14O5	265.04731	265.04728	0.1	1.4E+05
124	Hydroxyfuranochalcone	[M+H]+	C17H12O3	265.08604	265.08592	0.5	1.6E+05
125	Keto myristic acid	[M+Na]+	C14H26O3	265.17691	265.17742	-1.9	4.8E+05
126	Resveratrol	[M+K]+	C14H12O3	267.04235	267.04180	2.0	2.2E+05
127	Piceatannol	[M+Na]+	C14H12O4	267.06224	267.06278	-2.0	2.2E+05
128	Amino-tridecanoic acid	[M+K]+	C13H27NO2	268.16703	268.16734	-1.2	4.6E+05
129	Inosine	[M+H]+	C10H12N4O5	269.08749	269.08805	-2.1	1.7E+05
130	Oxo-pentyl-cyclopentanehexanoic acid	[M+H]+	C16H28O3	269.21063	269.21112	-1.8	1.1E+06

Table S5: Untargeted metabolic profiling of Goji Berries leaves hydroalcoholic extracts by ESI(+) FT-ICR MS

#	Plausible compound[a]	Ion	Molecular formula (M)	Theo. m/z[b]	Exp. m/z[c]	ppm	Intensity
131	Doxylamine	[M+H]+	C17H22N2O	271.18078	271.18049	1.1	2.6E+05
132	Axerophthene	[M+H]+	C20H30	271.24219	271.24203	0.6	1.1E+06
133	N-Feruloylglycine	[M+Na]+	C12H13NO5	274.06788	274.06859	-2.6	1.1E+05
134	Homoanserine	[M+Na]+	C11H18N4O3	277.12671	277.12711	-1.4	2.0E+05
135	Alhpa-tocopheronolactone	[M+H]+	C16H22O4	279.15948	279.15909	1.4	5.1E+05
136	Decenoylcholine	[M+Na]+	C15H30NO2	279.21693	279.21687	0.2	1.7E+06
137	Methyladenosine	[M+H]+	C11H15N5O4	282.11984	282.11968	0.6	1.5E+05
138	Amino-tetradecanoic acid	[M+K]+	C14H29NO2	282.18299	282.18299	0.0	3.7E+05
139	Aminoadenosine	[M+H]+	C10H14N6O4	283.11443	283.11493	-1.8	8.0E+05
140	Dehydroretinaldehyde	[M+H]+	C20H26O	283.20607	283.20564	1.5	6.5E+05
141	Hydroxy-heptadecen-ynoic acid	[M+H]+	C17H30O3	283.22656	283.22677	-0.7	1.4E+06
142	Ellipticine	[M+K]+	C17H14N2	285.07910	285.07886	0.8	1.6E+05
143	N2-(D-1-Carboxyethyl)-L-arginine	[M+K]+	C9H18N4O4	285.09579	285.09596	-0.6	2.0E+05
144	Cyclic bisphospho-D-glycerate	[M+K]+	C3H6O9P2	286.91245	286.91186	2.0	1.3E+05
145	Inosine	[M+Na]+	C10H12N4O5	291.07037	291.06999	1.3	2.5E+05
146	Dihydroxyisoflavone	[M+K]+	C15H10O4	293.02126	293.02107	0.7	1.6E+05
147	Methano-retinal	[M+H]+	C21H28O	297.22062	297.22129	-2.3	6.0E+05
148	Hydroxylinoleic acid	[M+H]+	C18H32O3	297.24195	297.24242	-1.6	1.5E+06
149	Liriodenine	[M+Na]+	C17H9NO3	298.04670	298.04746	-2.6	1.7E+05
150	Dihydroxy-methylenedioxy-phenylcoumarin	[M+H]+	C16H10O6	299.05530	299.05501	1.0	2.2E+05
151	Ranunculin	[M+Na]+	C11H16O8	299.07348	299.07374	-0.9	2.7E+05
152	Apigenin dimethylether	[M+H]+	C17H14O5	299.09216	299.09140	2.5	2.2E+05
153	Thalassemine	[M+H]+	C8H19N4O6P	299.11155	299.11150	0.2	2.5E+05
154	Cryptophorine	[M+K]+	C17H27NO	300.17187	300.17242	-1.8	1.4E+05
155	Sphingosine	[M+H]+	C18H37NO2	300.28902	300.28971	-2.3	2.5E+05
156	Alhpa-tocopheronolactone	[M+Na]+	C16H22O4	301.14082	301.14103	-0.7	6.1E+06

Table S5: Untargeted metabolic profiling of Goji Berries leaves hydroalcoholic extracts by ESI(+) FT-ICR MS

#	Plausible compound[a]	Ion	Molecular formula (M)	Theo. m/z[b]	Exp. m/z[c]	ppm	Intensity
157	Xylobiose	[M+Na]+	C10H18O9	305.08461	305.08430	1.0	3.3E+05
158	Matricin	[M+H]+	C17H22O5	307.15421	307.15400	0.7	4.8E+05
159	Cinnamodial	[M+H]+	C17H24O5	309.16884	309.16965	-2.6	4.1E+05
160	Hydroxy-(methoxyphenyl)-propylcoumarin	[M+H]+	C19H18O4	311.12791	311.12779	0.4	1.7E+05
161	Dihydroxy palmitic acid	[M+Na]+	C16H32O4	311.21851	311.21928	-2.5	2.3E+05
162	Dodecanoyl-sn-glycerol	[M+K]+	C15H30O4	313.17754	313.17757	-0.1	4.6E+05
163	Dehydrophytosphingosine	[M+H]+	C18H37NO3	316.28529	316.28462	2.1	5.5E+05
164	Tuliposide B	[M+Na]+	C11H18O9	317.08436	317.08430	0.2	4.1E+05
165	Leukotriene A4	[M+H]+	C20H30O3	319.22753	319.22677	2.4	8.1E+05
166	Thalassemine	[M+Na]+	C8H19N4O6P	321.09276	321.09344	-2.1	3.1E+05
167	Prosafrinine	[M+K]+	C17H33NO2	322.21380	322.21429	-1.5	3.2E+05
168	Dimethyl-phenyl-benzodipyranone	[M+Na]+	C20H16O3	327.09895	327.09917	-0.7	2.4E+05
169	Megastachine	[M+H]+	C20H29NO3	332.22150	332.22202	-1.6	7.6E+05
170	Hydroperoxy-octadecatrienoic acid	[M+Na]+	C18H30O4	333.20397	333.20363	1.0	3.8E+05
171	Jatrophone	[M+Na]+	C20H24O3	335.16155	335.16177	-0.6	2.6E+06
172	Hydroperoxy-eicosadienoic acid	[M+H]+	C20H36O4	341.26835	341.26864	-0.8	1.5E+06
173	Gynocardin	[M+K]+	C12H17NO8	342.05934	342.05858	2.2	2.3E+05
174	N-[(Indolyl)acetyl]-L-lysine	[M+K]+	C16H21N3O3	342.12047	342.12145	-2.9	1.9E+05
175	Catechin methyl ether	[M+K]+	C16H16O6	343.05884	343.05785	2.9	2.0E+05
176	Tetramethoxyflavanone	[M+H]+	C19H20O6	345.13257	345.13326	-2.0	4.7E+05
177	Gingerol	[M+Na]+	C19H30O4	345.20415	345.20363	1.5	4.7E+05
178	Cularimine	[M+Na]+	C19H21NO4	350.13526	350.13628	-2.9	2.1E+05
179	Crocetin	[M+Na]+	C20H24O4	351.15710	351.15668	1.2	3.7E+05
180	PC(O-2:0/O-2:0)	[M+K]+	C12H28NO6P	352.12877	352.12858	0.5	3.0E+05
181	Capnine	[M+H]+	C17H37NO4S	352.25059	352.25161	-2.9	1.7E+05
182	Leukotriene B4	[M+Na]+	C20H32O4	359.22033	359.21928	2.9	3.6E+05

Table S5: Untargeted metabolic profiling of Goji Berries leaves hydroalcoholic extracts by ESI(+) FT-ICR MS

#	Plausible compound[a]	Ion	Molecular formula (M)	Theo. m/z[b]	Exp. m/z[c]	ppm	Intensity
183	PC(O-5:0/0:0)	[M+K]+	C13H30NO6P	366.14511	366.14423	2.4	5.5E+05
184	Keto-decanoylcarnitine	[M+K]+	C17H31NO5	368.18283	368.18338	-1.5	3.2E+05
185	Dihydroxy-trimethoxyflavanone	[M+Na]+	C18H18O7	369.09401	369.09447	-1.3	3.3E+05
186	Alpha-dihydroxy-pentanorcholecalciferol	[M+Na]+	C22H34O3	369.23898	369.24002	-2.8	9.3E+05
187	Carnosic acid	[M+K]+	C20H28O4	371.16228	371.16192	1.0	2.1E+05
188	Gibberellin A2	[M+Na]+	C19H26O6	373.16176	373.16216	-1.1	1.7E+05
189	Usaramine	[M+Na]+	C18H25NO6	374.15773	374.15741	0.9	1.8E+05
190	Coumestrol diacetate	[M+Na]+	C19H12O7	375.04781	375.04752	0.8	8.8E+05
191	Vincamine	[M+Na]+	C21H26N2O3	377.18285	377.18356	-1.9	2.5E+05
192	MG(0:0/18:2/0:0)	[M+Na]+	C21H38O4	377.26699	377.26623	2.0	1.7E+05
193	Reduced riboflavin	[M+H]+	C17H22N4O6	379.16018	379.16121	-2.7	3.0E+06
194	Docosanoic acid	[M+K]+	C22H44O2	379.29650	379.29729	-2.1	3.3E+05
195	MG(0:0/20:2/0:0)	[M+H]+	C23H42O4	383.31489	383.31559	-1.8	2.3E+06
196	Isopentenyladenine-9-N-glucoside	[M+Na]+	C17H25N5O4	386.18020	386.17988	0.8	2.1E+05
197	Leukotriene B4 dimethylamide	[M+Na]+	C22H37NO3	386.26682	386.26656	0.7	2.0E+05
198	Cinnamoyl-dihydro-dihydroxy-phenylcoumarin	[M+H]+	C24H18O5	387.12235	387.12270	-0.9	1.9E+05
199	Gibberellin A28	[M+H]+	C20H26O8	395.17002	395.17004	-0.1	5.5E+05
200	O-Demethyl-O-deacetylvindoline	[M+H]+	C22H28N2O5	401.20661	401.20710	-1.2	3.5E+05
201	Tetradecenoylcarnitine	[M+K]+	C21H39NO4	408.25086	408.25107	-0.5	3.2E+05
202	Dihydroxy-methoxy-(methylbutyryloxy)chalcone	[M+K]+	C21H22O6	409.10533	409.10480	1.3	2.5E+05
203	Hydroxy-hexacosanoic acid	[M+H]+	C26H52O3	413.39931	413.39892	0.9	1.2E+06
204	N-stearoyl taurine	[M+Na]+	C20H41NO4S	414.26543	414.26485	1.4	9.1E+05
205	Tetrahydroxy-trimethoxyflavone	[M+K]+	C18H16O9	415.04179	415.04259	-1.9	2.0E+05
206	8-C-p-Hydroxybenzylkaempferol	[M+Na]+	C22H16O7	415.07766	415.07882	-2.8	2.5E+05
207	Hexanoyl-alpha-glucopyranose	[M+K]+	C18H32O8	415.17378	415.17288	2.2	3.4E+05
208	Hyaluronic acid	[M+H]+	C16H27NO12	426.16085	426.16060	0.6	3.0E+05

Table S5: Untargeted metabolic profiling of Goji Berries leaves hydroalcoholic extracts by ESI(+) FT-ICR MS

#	Plausible compound[a]	Ion	Molecular formula (M)	Theo. m/z[b]	Exp. m/z[c]	ppm	Intensity
209	Geranylchrysin	[M+K]+	C25H26O4	429.14577	429.14627	-1.2	3.1E+05
210	Rehmaionoside A	[M+K]+	C19H34O8	429.18883	429.18853	0.7	2.9E+05
211	Calcitriol	[M+Na]+	C27H44O3	439.31921	439.31827	2.1	3.1E+05
212	Lamioside	[M+Na]+	C18H28O11	443.15149	443.15238	-2.0	2.7E+05
213	Lauryl oleate	[M+Na]+	C28H54O2	445.40120	445.40160	-0.9	2.4E+05
214	Hydroxy-gama-tocotrienol	[M+Na]+	C28H42O3	449.30138	449.30262	-2.8	3.8E+05
215	Triacontadienoic acid	[M+H]+	C30H56O2	449.43464	449.43531	-1.5	3.1E+05
216	Catechin-O-(hydroxy-oxo-cyclohexene-carboxylate)	[M+Na]+	C22H20O9	451.10113	451.09995	2.6	1.6E+05
217	MG(0:0/22:0/0:0)	[M+K]+	C25H50O4	453.33412	453.33407	0.1	2.1E+05
218	PC(6:0/6:0)	[M+H]+	C20H40NO8P	454.25640	454.25643	-0.1	1.7E+05
219	PA(18:3/0:0)	[M+Na]+	C21H37O7P	455.21705	455.21691	0.3	5.0E+05
220	S-Octyl glutathione	[M+K]+	C18H33N3O6S	458.17136	458.17217	-1.8	1.8E+05
221	PG(13:0/0:0)	[M+Na]+	C19H39O9P	465.22227	465.22239	-0.3	3.5E+05
222	PA(20:0/0:0)	[M+H]+	C23H47O7P	467.31456	467.31322	2.9	2.8E+05
223	Hexadecanedioic acid mono-carnitine ester	[M+K]+	C23H43NO6	468.27229	468.27220	0.2	2.3E+05
224	TG(8:0/8:0/8:0)	[M+H]+	C27H50O6	471.36742	471.36802	-1.3	3.3E+06
225	PA(19:1/0:0)	[M+Na]+	C22H43O7P	473.26319	473.26386	-1.4	3.5E+05
226	PE(O-16:0/0:0)	[M+K]+	C21H46NO6P	478.26919	478.26943	-0.5	2.5E+05
227	PC(P-16:0/0:0)	[M+H]+	C24H50NO6P	480.34583	480.34485	2.0	2.0E+05
228	Alpha-dihydroxyvitamin D3-lactone	[M+K]+	C27H40O5	483.25178	483.25073	2.2	3.5E+05
229	Hydroxy-alpha-tocopherol	[M+K]+	C29H50O3	485.33904	485.33915	-0.2	2.0E+05
230	PE(17:2/0:0)	[M+Na]+	C22H42NO7P	486.26041	486.25911	2.7	2.2E+05
231	Dracorubin	[M+H]+	C32H24O5	489.17035	489.16965	1.4	1.8E+05
232	Sophoraisoflavanone D	[M+H]+	C30H36O6	493.25915	493.25847	1.4	2.0E+05
233	Spinosic acid A	[M+Na]+	C30H48O4	495.34301	495.34448	-3.0	1.2E+05

Table S5: Untargeted metabolic profiling of Goji Berries leaves hydroalcoholic extracts by ESI(+) FT-ICR MS

#	Plausible compound[a]	Ion	Molecular formula (M)	Theo. m/z[b]	Exp. m/z[c]	ppm	Intensity
234	Cucurbitacin S	[M+H]+	C30H42O6	499.30587	499.30542	0.9	1.2E+05
235	Tetradehydrolycopene	[M+H]+	C40H52	533.41410	533.41418	-0.1	1.2E+05
236	UDP-amino-deoxy-arabinose	[M+H]+	C14H23N3O15P2	536.06816	536.06772	0.8	1.2E+05
237	Iridodial glucoside tetraacetate	[M+K]+	C24H34O11	537.17452	537.17327	2.3	9.5E+04
238	PC(16:0/2:0)	[M+H]+	C26H52NO8P	538.35120	538.35033	1.6	1.3E+05
239	Dimethylpyraono-kaempferol-methyl ether-rhamnoside	[M+K]+	C27H28O10	551.13035	551.13141	-1.9	8.3E+04
240	Flavonol-O-D-xylosylglucoside	[M+Na]+	C26H28O12	555.14769	555.14730	0.7	1.3E+05
241	Cucurbitacin D	[M+K]+	C30H44O7	555.27127	555.27186	-1.1	7.3E+04
242	PC(18:3/0:0)	[M+K]+	C26H48NO7P	556.27907	556.28000	-1.7	8.4E+04
243	PI(O-16:0/0:0)	[M+H]+	C25H51O11P	559.32376	559.32418	-0.7	9.8E+04
244	Flavonol-O-D-xylosylglucoside	[M+K]+	C26H28O12	571.12178	571.12124	1.0	2.0E+05
245	Myristinin A	[M+Na]+	C33H40O7	571.26543	571.26662	-2.1	1.1E+05
246	Cer(d18:2/16:0)	[M+K]+	C34H65NO3	574.45851	574.45960	-1.9	8.4E+04
247	Hydroxy-b,e-caroten-one	[M+Na]+	C40H54O2	589.40311	589.40160	2.6	9.7E+04
248	Celapanine	[M+K]+	C30H35NO10	608.19095	608.18926	2.8	1.2E+05
249	Peonidin-(p-coumarylglucoside)	[M+H]+	C31H29O13	610.16887	610.16809	1.3	9.9E+04
250	Isorhamnetin-glucuronide-sulfate	[M+K]+	C22H20O16S	611.01158	611.01037	2.0	9.6E+04
251	PA(14:1/14:1)	[M+Na]+	C31H57O8P	611.36982	611.36833	2.4	8.6E+04
252	Myricetin-(galloylrhamnoside)	[M+H]+	C28H24O16	617.11284	617.11371	-1.4	1.5E+05
253	Naringenin-(p-Coumaroylglucoside)	[M+K]+	C30H28O12	619.12272	619.12124	2.4	9.3E+04
254	PI(P-18:0/0:0)	[M+K]+	C27H53O11P	623.29645	623.29571	1.2	1.1E+05
255	Kanokoside D	[M+H]+	C27H44O16	625.26971	625.27021	-0.8	7.5E+04
256	Pelargonidin 3-O-O-dimalonylglucoside	[M+Na]+	C27H25O16	628.10241	628.10348	-1.7	9.4E+04
257	Calebassone	[M+K]+	C40H48N4O2	655.34228	655.34089	2.1	8.1E+04
258	DG(18:3/22:6/0:0)	[M+H]+	C43H66O5	663.49712	663.49830	-1.8	8.2E+04

Table S5: Untargeted metabolic profiling of Goji Berries leaves hydroalcoholic extracts by ESI(+) FT-ICR MS

#	Plausible compound[a]	Ion	Molecular formula (M)	Theo. m/z[b]	Exp. m/z[c]	ppm	Intensity
259	PA(O-18:0/14:0)	[M+K]+	C35H71O7P	673.45549	673.45690	-2.1	7.2E+04
260	PG(12:0/13:0)	[M+K]+	C31H64NO10P	680.39196	680.38994	3.0	8.8E+04
261	PE(15:0/P-16:0)	[M+Na]+	C36H72NO7P	684.49317	684.49386	-1.0	7.9E+04
262	Pelargonidin-O-[O-(beta-D-xylopyranosyl)O-(methyl-malonyl)-bata-galactopyranoside]	[M+Na]+	C30H33O17	688.16224	688.16100	1.8	7.2E+04
263	Quercetin-(diacetylrhamnosyl)-glucoside	[M+H]+	C31H34O18	695.18166	695.18179	-0.2	7.6E+04
264	PE(15:0/16:0)	[M+Na]+	C36H72NO8P	700.48874	700.48878	-0.1	1.0E+05
265	DG(22:6/22:6/0:0)	[M+H]+	C47H68O5	713.51458	713.51395	0.9	1.2E+05
266	PS(14:1/16:1)	[M+Na]+	C36H66NO10P	726.43084	726.43165	-1.1	1.3E+05
267	PA(O-16:0/20:1)	[M+K]+	C39H77O7P	727.50523	727.50385	1.9	1.8E+06
268	Methyltetrahydropteroyltri-glutamate	[M+Na]+	C30H39N9O12	740.26013	740.26104	-1.2	1.4E+05
269	DG(18:0/24:1/0:0)	[M+K]+	C45H86O5	745.61254	745.61069	2.5	1.3E+05
270	Quercetin-(xylosylrutinoside)	[M+Na]+	C32H38O20	765.18550	765.18486	0.8	9.0E+04
271	PE(18:1/P-18:1)	[M+K]+	C41H78NO7P	766.51249	766.51475	-2.9	1.0E+05
272	PE(14:0/24:1(15Z))	[M+H]+	C43H84NO8P	774.59922	774.60073	-2.0	7.8E+04
273	Cyanidin-(caffeylsambubioside)	[M+K]+	C35H35O18	782.14549	782.14550	0.0	1.6E+05
274	PG(16:0/18:1)	[M+K]+	C40H80NO10P	804.51458	804.51514	-0.7	1.0E+05
275	PS(18:1/20:4)]	[M+H]+	C44H76NO10P	810.52702	810.52796	-1.2	7.8E+04
276	PG(O-18:0/22:4)	[M+H]+	C46H85O9P	813.59978	813.60040	-0.8	8.1E+04
277	PE(18:0/22:4)	[M+Na]+	C45H82NO8P	818.56804	818.56703	1.2	1.1E+05
278	Laricitrin-rutinoside-glucoside	[M+H]+	C34H44O23	821.23499	821.23461	0.5	1.2E+05
279	PG(18:3/22:6)	[M+Na]+	C46H73O10P	839.48282	839.48336	-0.6	7.9E+04
280	PS(22:1/22:6)	[M+Na]+	C50H84NO10P	912.57280	912.57251	0.3	1.3E+05
281	Hydroxyphenylacetyl-CoA	[M+Na]+	C29H42N7O18P3S	924.14343	924.14121	2.4	9.5E+04
282	Kaempferol-neohesperidoside-(ferulylglucoside)	[M+H]+	C43H48O23	933.26759	933.26591	1.8	1.6E+05
283	TG(17:1/20:5/20:5)	[M+Na]+	C60H94O6	933.69312	933.69426	-1.2	9.8E+04

Table S5: Untargeted metabolic profiling of Goji Berries leaves hydroalcoholic extracts by ESI(+) FT-ICR MS

#	**Plausible compound**[a]	**Ion**	**Molecular formula (M)**	**Theo. m/z**[b]	**Exp. m/z**[c]	**ppm**	**Intensity**
284	PI(17:1/22:4)	[M+K]+	C48H83O13P	937.52204	937.52029	1.9	1.2E+05
285	PC(22:4/24:0)	[M+K]+	C54H100NO8P	960.68144	960.68182	-0.4	1.3E+05
286	PIP(16:0/20:2)	[M+Na]+	C45H84O16P2	965.51160	965.51268	-1.1	1.1E+05

a MG Monoacylglycerol; DG Diacylglycerol; TG Triacylglycerol; GalCer Galactosylceramide; PA Phosphatidic acid; PC Phosphatidylcholine; PE Phosphatidylethanolamine; PG Glycerophospholipids; PI Phosphatidylinositol; PS Phosphatidylserine; SQMG Glycosylmonoacylglycerols; DHAP Dihydroxyacetone phosphate; DNP Dinitrophenyl

b Theoretical m/z

c experimental m/z

Table S6: Untargeted metabolic profiling of Goji Berries leaves hydroalcoholic extracts by ESI(-) FT-ICR MS

#	Putative Annotation[a]	Ion	Molecular formula (M)	Theo. m/z[b]	Exp. m/z[c]	ppm	Intensity
1	1,4-Lactone	[M-H]-	C4H6O2	85.02950	85.02974	2.8	1.5E+06
2	Glycerol	[M-H]-	C3H8O3	91.04007	91.04018	1.2	1.2E+06
3	Cyclopropylamine	[M+Cl]-	C3H7N	92.02725	92.02736	1.2	1.1E+06
4	Sulfate	[M-H]-	H2SO4	96.96010	96.96006	-0.4	1.6E+06
5	Succinimide	[M-H]-	C4H5NO2	98.02475	98.02463	-1.2	1.9E+06
6	Hexylamine	[M-H]-	C6H15N	100.11317	100.11347	3.0	1.2E+06
7	Benzaldehyde	[M-H]-	C7H6O	105.03459	105.03452	-0.7	1.0E+06
8	1-Butanol	[M+Cl]-	C4H10O	109.04257	109.04288	2.9	1.3E+06
9	Piperidine	[M+Cl]-	C5H11N	120.05855	120.05824	-2.6	1.8E+06
10	Oxalic acid	[M+Cl]-	C2H2O4	124.96471	124.96455	-1.3	1.1E+06
11	Dihydrothymine	[M-H]-	C5H8N2O2	127.05130	127.05110	-1.6	6.2E+06
12	Oxaloacetate	[M-H]-	C4H4O5	130.99860	130.99882	1.7	1.7E+06
13	L-Asparagine	[M-H]-	C4H8N2O3	131.04622	131.04617	-0.3	1.4E+06
14	(S)-Malate	[M-H]-	C4H6O5	133.01425	133.01436	0.9	1.7E+06
15	Cinnamyl alcohol	[M-H]-	C9H10O	133.06589	133.06572	-1.3	1.7E+06
16	Tyramine	[M-H]-	C8H11NO	136.07679	136.07656	-1.7	1.2E+06
17	Salicylic acid	[M-H]-	C7H6O3	137.02442	137.02468	1.9	1.8E+06
18	cis-4-Carboxymethylenebut-2-en-4-olide	[M-H]-	C6H4O4	139.00368	139.00405	2.6	1.2E+06
19	4Z,7-octadienoic acid	[M-H]-	C8H12O2	139.07645	139.07681	2.6	3.4E+06
20	2E-Nonen-1-ol	[M-H]-	C9H18O	141.12849	141.12862	0.9	3.8E+06
21	2,3-Dimethylmaleate	[M-H]-	C6H8O4	143.03498	143.03468	-2.1	1.2E+06
22	L-Lysine	[M-H]-	C6H14N2O2	145.09825	145.09843	1.2	1.7E+06
23	L-Glutamate	[M-H]-	C5H9NO4	146.04588	146.04548	-2.7	2.2E+06
24	trans-Cinnamate	[M-H]-	C9H8O2	147.04515	147.04552	2.5	1.4E+06
25	cis-Acetylacrylate	[M+Cl]-	C5H6O3	149.00110	149.00126	1.1	1.4E+06
26	Thiopurine	[M-H]-	C5H4N4S	151.00839	151.00881	2.8	1.3E+06

Table S6: Untargeted metabolic profiling of Goji Berries leaves hydroalcoholic extracts by ESI(-) FT-ICR MS

#	Putative Annotation[a]	Ion	Molecular formula (M)	Theo. m/z[b]	Exp. m/z[c]	ppm	Intensity
27	L-Arabitol	[M-H]-	C5H12O5	151.06120	151.06163	2.9	2.0E+06
28	L-Aspartate 4-semialdehyde	[M+Cl]-	C4H7NO3	152.01199	152.01168	-2.1	1.1E+06
29	Succinate	[M+Cl]-	C4H6O4	152.99601	152.99609	0.5	1.2E+06
30	Borneol	[M-H]-	C10H18O	153.12849	153.12858	0.6	2.0E+06
31	L-Histidine	[M-H]-	C6H9N3O2	154.06220	154.06205	-1.0	1.9E+06
32	2-methyl-octanoic acid	[M-H]-	C9H18O2	157.12340	157.12338	-0.1	2.8E+06
33	delta-Guanidinovaleric acid	[M-H]-	C6H13N3O2	158.09350	158.09329	-1.3	1.9E+06
34	2-Propylsuccinic acid	[M-H]-	C7H12O4	159.06628	159.06607	-1.3	1.9E+06
35	Pyrogallol	[M+Cl]-	C6H6O3	161.00110	161.00151	2.6	2.3E+06
36	Melamine	[M+Cl]-	C3H6N6	161.03480	161.03476	-0.2	1.8E+06
37	(R)-3,3-Dimethylmalate	[M-H]-	C6H10O5	161.04555	161.04598	2.7	1.3E+06
38	Methyl cinnamate	[M-H]-	C10H10O2	161.06080	161.06125	2.8	1.1E+06
39	Nicotine	[M-H]-	C10H14N2	161.10842	161.10828	-0.9	1.3E+06
40	7-Methylxanthine	[M-H]-	C6H6N4O2	165.04180	165.04219	2.4	9.7E+05
41	L-Asparagine	[M+Cl]-	C4H8N2O3	167.02289	167.02318	1.7	1.6E+06
42	Decanoic acid	[M-H]-	C10H20O2	171.13905	171.13916	0.6	2.5E+06
43	5-Hydroxymethyluracil	[M+Cl]-	C5H6N2O3	177.00724	177.00706	-1.0	9.9E+05
44	7,8-Dihydroxycoumarin	[M-H]-	C9H6O4	177.01933	177.01925	-0.5	9.6E+05
45	O-Phospho-L-serine	[M-H]-	C3H8NO6P	184.00165	184.00120	-2.4	9.2E+05
46	3-methyl-decanoic acid	[M-H]-	C11H22O2	185.15470	185.15498	1.5	9.5E+05
47	3-Isopropylcatechol	[M+Cl]-	C9H12O2	187.05313	187.05329	0.8	8.7E+05
48	Quinic acid	[M-H]-	C7H12O6	191.05611	191.05603	-0.4	1.6E+06
49	Phthalate	[M+Cl]-	C8H6O4	200.99601	200.99646	2.2	1.1E+06
50	Cysteic acid	[M+Cl]-	C3H7NO5S	203.97389	203.97422	1.6	1.1E+06
51	Pantothenol	[M-H]-	C9H19NO4	204.12413	204.12453	2.0	7.6E+05
52	Gallic acid	[M+Cl]-	C7H6O5	204.99092	204.99056	-1.8	2.5E+06

Table S6: Untargeted metabolic profiling of Goji Berries leaves hydroalcoholic extracts by ESI(-) FT-ICR MS

#	Putative Annotation[a]	Ion	Molecular formula (M)	Theo. m/z[b]	Exp. m/z[c]	ppm	Intensity
53	Anthocyanin	[M-H]-	C15H11O	206.07371	206.07395	1.1	7.3E+05
54	decanamide	[M+Cl]-	C10H21NO	206.13172	206.13143	-1.4	9.8E+05
55	Menadione	[M+Cl]-	C11H8O2	207.02183	207.02236	2.6	6.8E+05
56	2-Octenedioic acid	[M+Cl]-	C8H12O4	207.04296	207.04298	0.1	1.1E+06
57	L-Citrulline	[M+Cl]-	C6H13N3O3	210.06509	210.06559	2.4	1.0E+06
58	(S)-6-Hydroxynicotine	[M+Cl]-	C10H14N2O	213.08001	213.07994	-0.4	2.5E+06
59	3-Oxododecanoic acid	[M-H]-	C12H22O3	213.14962	213.14964	0.1	7.5E+05
60	2-methyl lauric acid	[M-H]-	C13H26O2	213.18600	213.18657	2.7	2.7E+06
61	Succinyl proline	[M-H]-	C9H13NO5	214.07210	214.07226	0.8	9.3E+05
62	Caffeic acid	[M+Cl]-	C9H8O4	215.01166	215.01177	0.5	2.4E+06
63	D-Glucose	[M+Cl]-	C6H12O6	215.03279	215.03219	-2.8	1.1E+06
64	Diisopropyl phosphate	[M+Cl]-	C6H15O4P	217.04020	217.04018	-0.1	1.7E+06
65	Myristoleic acid	[M-H]-	C14H26O2	225.18600	225.18653	2.3	2.2E+06
66	Tetradecanoic acid	[M-H]-	C14H28O2	227.20165	227.20161	-0.2	2.4E+07
67	Phenylacetylglycine	[M+Cl]-	C10H11NO3	228.04329	228.04308	-0.9	1.2E+06
68	Ferulic acid	[M+Cl]-	C10H10O4	229.02731	229.02754	1.0	2.1E+06
69	2-pentadecenoic acid	[M-H]-	C15H28O2	239.20165	239.20188	0.9	3.0E+06
70	Palmitic acid	[M-H]-	C16H32O2	255.23295	255.23238	-2.2	4.2E+07
71	5,8,11-heptadecatriynoic acid	[M-H]-	C17H22O2	257.15470	257.15431	-1.5	9.0E+05
72	Norlinolenic acid	[M-H]-	C17H28O2	263.20165	263.20139	-1.0	8.9E+05
73	7-methyl-6E-hexadecenoic acid	[M-H]-	C17H32O2	267.23295	267.23349	2.0	4.0E+06
74	Oleic acid	[M-H]-	C18H34O2	281.24860	281.24849	-0.4	1.1E+07
75	Stearic acid	[M-H]-	C18H36O2	283.26425	283.26484	2.1	1.1E+07
76	14:4(2E,4E,8E,10E)(6Me[S],9Me,12Me[S])	[M+Cl]-	C17H26O2	297.16268	297.16239	-1.0	3.3E+06
77	3',4'-Dihydroxy-5,7-dimethoxyflavan	[M-H]-	C17H18O5	301.10815	301.10794	-0.7	1.2E+06
78	Stearidonic acid	[M+Cl]-	C18H28O2	311.17833	311.17894	2.0	2.3E+07

Table S6: Untargeted metabolic profiling of Goji Berries leaves hydroalcoholic extracts by ESI(-) FT-ICR MS

#	Putative Annotation[a]	Ion	Molecular formula (M)	Theo. m/z[b]	Exp. m/z[c]	ppm	Intensity
79	4-Oxo-13-cis-retinoate	[M-H]-	C20H25O3	312.17309	312.17395	2.7	4.9E+06
80	N-Arachidonoyl glycine	[M-H]-	C22H35NO3	360.25442	360.25451	0.3	7.3E+05
81	5alpha-Androstan-17beta-ol propionate	[M+Cl]-	C22H36O2	367.24093	367.24188	2.6	6.3E+05
82	Eicosanedioic acid	[M+Cl]-	C20H38O4	377.24641	377.24636	-0.1	5.1E+05
83	11,12-dihydroxy arachidic acid	[M+Cl]-	C20H40O4	379.26206	379.26259	1.4	9.5E+05
84	C17 Sphingosine-1-phosphate	[M-H]-	C17H39N2O5P	381.25238	381.25298	1.6	7.1E+05
85	MG(0:0/18:1(11Z)/0:0)	[M+Cl]-	C21H40O4	391.26206	391.26187	-0.5	5.0E+05
86	Campestanol	[M-H]-	C28H50O	401.37889	401.37956	1.7	4.9E+05
87	PG(18:1(9Z)/0:0)	[M+Cl]-	C24H47O9P	545.26517	545.26418	-1.8	1.9E+06
88	PG(22:6(4Z,7Z,10Z,13Z,16Z,19Z)/0:0)	[M-H]-	C28H45O9P	555.27284	555.27224	-1.1	5.9E+07
89	Delphinine	[M-H]-	C33H45NO9	598.30216	598.30333	2.0	1.8E+06
90	Kaempferol 7,4'-dimethyl ether 3-(6''-(E)-p-coumarylglucoside)	[M-H]-	C32H30O13	621.16136	621.16067	-1.1	1.1E+06
91	DG(20:3(5Z,8Z,11Z)/22:6(4Z,7Z,10Z,13Z,16Z,19Z)/0:0)	[M-H]-	C45H70O5	689.51505	689.51579	1.1	1.0E+06
92	PA(12:0/22:4(7Z,10Z,13Z,16Z))	[M+Cl]-	C37H65O8P	703.41111	703.41134	0.3	5.4E+05
93	SM(d18:1/16:0)	[M+Cl]-	C39H80N2O6P	738.54480	738.54573	1.3	6.6E+05
94	PG(16:0/18:3(6Z,9Z,12Z))	[M-H]-	C40H73O10P	743.48686	743.48877	2.6	6.6E+05
95	PC(15:0/18:0)	[M-H]-	C41H82NO8P	746.57053	746.56881	-2.3	7.0E+05
96	GlcCer(d18:0/18:0)	[M+Cl]-	C42H83NO8	764.58127	764.58294	2.2	6.1E+05
97	PE(18:3(6Z,9Z,12Z)/22:6(4Z,7Z,10Z,13Z,16Z,19Z))	[M+Cl]-	C45H72NO8P	820.46896	820.46806	-1.1	1.1E+06
98	PI(O-16:0/17:1(9Z))	[M+Cl]-	C42H81O12P	843.51597	843.51552	-0.5	7.0E+05
99	PC(20:5(5Z,8Z,11Z,14Z,17Z)/24:1(15Z))	[M+Cl]-	C52H92NO8P	924.62546	924.62456	-1.0	6.9E+05
100	TG(17:1(9Z)/20:1(11Z)/20:1(11Z))[iso3]	[M-H]-	C60H110O6	925.82296	925.82546	2.7	7.4E+05

a MG Monoacylglycerol; DG Diacylglycerol; TG Triacylglycerol; GalCer Galactosylceramide; PA Phosphatidic acid; PC Phosphatidylcholine; PE Phosphatidylethanolamine; PG Glycerophospholipids; PI Phosphatidylinositol; PS Phosphatidylserine; SQMG Glycosylmonoacylglycerols; DHAP Dihydroxyacetone phosphate; DNP Dinitrophenyl

Table S6: Untargeted metabolic profiling of Goji Berries leaves hydroalcoholic extracts by ESI(-) FT-ICR MS

#	Putative Annotation[a]	Ion	Molecular formula (M)	Theo. m/z[b]	Exp. m/z[c]	ppm	Intensity

b Theoretical m/z

c experimental m/z

Table S7: Untargeted metabolic profiling of Goji Berries leaves organic extracts by ESI(+) Ion Trap MS[a]

#	m/z	Intensity	Putative Annotation[b]	Molecular formula (M)	Ion
1	131.8	1.7E+03	Leucine/Isoleucine	C6H13NO2	[M+H]+
2	140.1	5.1E+03	Valine	C5H11NO2	[M+Na]+
3	156.1	5.7E+03	Histidine	C6H9N3O2	[M+H]+
4	160.1	1.5E+03	Trigonelline	C7H7NO2	[M+Na]+
5	163.1	1.7E+03	Dimethylmalic acid	C6H10O5	[M+H]+
6	164.1	1.5E+03	S-(carboxypropyl)-Cysteamine	C6H13NO2S	[M+H]+
7	176.1	6.1E+03	Citrulline	C6H13N3O3	[M+H]+
8	203.1	4.0E+03	Aldohexose	C6H12O6	[M+Na]+
9	210.1	2.0E+03	Butenyl-methyl-threonine	C9H17NO3	[M+Na]+
10	219.1	1.7E+03	Aldohexose	C6H12O6	[M+K]+
11	229.1	1.6E+03	Myristic acid	C14H28O2	[M+H]+
12	244.9	2.0E+03	L-Uridine	C9H12N2O6	[M+H]+
13	252.9	1.9E+03	Dodecanedioic acid	C12H22O4	[M+Na]+
14	260.9	1.9E+03	Methoxychalcone	C16H14O2	[M+Na]+
15	270.9	1.6E+03	Apigenin	C15H10O5	[M+H]+
16	273.0	1.8E+03	Naringenin	C15H12O5	[M+H]+
17	277.0	3.7E+03	Chrysanthetriol	C15H26O3	[M+H]+
18	279.1	2.9E+03	Palmitic Acid	C16H32O2	[M+Na]+
19	280.9	1.7E+03	Apigeniflavan	C15H14O4	[M+Na]+
20	283.1	1.6E+03	Oleic acid	C18H34O2	[M+H]+
21	284.3	2.7E+03	Stearamide	C18H37NO	[M+H]+
22	289.1	2.0E+03	Dihydroretinol	C20H32O	[M+H]+
23	291.0	2.2E+03	Catechin	C15H14O6	[M+H]+
24	293.0	2.6E+03	Apigenin	C15H10O5	[M+Na]+
25	295.0	2.2E+03	Hydroxylinolenic acid	C18H30O3	[M+H]+
26	297.1	2.1E+03	Hydroxy-octadecadienoic acid	C18H32O3	[M+H]+

Table S7: Untargeted metabolic profiling of Goji Berries leaves organic extracts by ESI(+) Ion Trap MS[a]

#	m/z	Intensity	Putative Annotation[b]	Molecular formula (M)	Ion
27	299.1	2.0E+03	Oxooctadecanoic acid	C18H34O3	[M+H]+
28	301.1	2.9E+03	Hydroxystearic acid	C18H36O3	[M+H]+
29	303.0	3.2E+03	Quercetin	C15H10O7	[M+H]+
30	305.0	3.4E+03	Oleic Acid	C18H34O2	[M+Na]+
31	306.9	4.2E+03	Stearic acid	C18H36O2	[M+Na]+
32	309.1	2.2E+03	Fructoselysine	C12H24N2O7	[M+H]+
33	315.0	3.2E+03	Octadecanedioic acid	C18H34O4	[M+H]+
34	317.0	3.8E+03	Octadienoic acid, methyl ester	C19H34O2	[M+Na]+
35	318.9	3.2E+03	Myricetin (triacetylxyloside)	C19H36O2	[M+H]+
36	321.0	1.2E+03	Octadecanoic acid, methyl ester	C19H38O2	[M+Na]+
37	322.9	3.4E+03	Trimethoxyflavan	C18H20O4	[M+Na]+
38	324.9	3.2E+03	Fructofuranose dianhydride	C12H20O10	[M+H]+
39	327.0	3.2E+03	Octadecanoic acid, methyl ester	C19H38O2	[M+Na]+
40	337.0	4.4E+03	Octadecanoic acid, methyl ester	C19H38O2	[M+K]+
41	339.0	4.4E+03	Dihydroxy stearic acid	C18H36O4	[M+Na]+
42	341.0	3.0E+03	Trihydroxy-dimethoxyisoflavan	C17H18O6	[M+Na]+
43	346.9	3.7E+03	Portulacaxanthin I	C14H16N2O7	[M+Na]+
44	348.9	2.3E+03	Trihydroxy-prenyldihydrochalcone	C20H22O4	[M+Na]+
45	351.0	3.8E+03	MG(16:1/0:0/0:0)	C19H36O4	[M+Na]+
46	353.0	5.5E+03	MG(16:0/0:0/0:0)	C19H38O4	[M+Na]+
47	359.0	2.1E+03	Catharanthine	C21H24N2O2	[M+Na]+
48	362.9	3.0E+03	Neobanol	C18H12O6	[M+Na]+
49	365.0	8.1E+03	Disaccharide	C12H22O11	[M+Na]+
50	367.0	4.2E+03	Hydroxydocosahexaenoic acid	C22H32O3	[M+Na]+
51	369.0	4.7E+03	MG(0:0/16:0/0:0)	C19H38O4	[M+K]+
52	371.1	3.7E+03	Docosanedioic acid	C22H42O4	[M+H]+

Table S7: Untargeted metabolic profiling of Goji Berries leaves organic extracts by ESI(+) Ion Trap MS[a]

#	m/z	Intensity	Putative Annotation[b]	Molecular formula (M)	Ion
53	377.0	8.5E+03	Chlorogenic acid	C16H18O9	[M+Na]+
54	379.1	3.1E+03	O-Feruloyl-Dglucose Dihydroxymethoxy	C16H20O9	[M+Na]+
55	381.0	8.1E+03	Disaccharide	C12H22O11	[M+K]+
56	383.0	4.1E+03	Mycocerosic acid	C25H50O2	[M+H]+
57	385.0	4.1E+03	Dihydroxy-tetramethoxyisoflavan	C19H22O7	[M+Na]+
58	387.0	3.3E+03	Anacardic acid	C22H36O3	[M+K]+
59	389.1	2.2E+03	Tetracosenoic acid	C24H46O2	[M+Na]+
60	391.1	3.9E+03	Diisooctyl phthalate	C24H38O4	[M+H]+
61	393.1	6.2E+03	Pentahydroxy-trimethoxyflavone	C18H16O10	[M+H]+
62	395.0	4.4E+03	Hydroxy-trimethoxy-methylenedioxyflavone	C19H16O8	[M+Na]+
63	397.0	4.5E+03	Dihydroxy-tetramethoxyflavone	C19H18O8	[M+Na]+
64	399.0	4.5E+03	Hydroxy-pentamethoxyisoflavan	C20H24O7	[M+Na]+
65	401.0	4.9E+03	Dihydroxy-methoxy- (hydroxybenzyl) dihydrochalcone	C19H22O8	[M+Na]+
66	405.1	2.4E+03	Glucopyranuronic acid	C19H26O8	[M+Na]+
67	407.1	5.2E+03	Dihydroxy-dimethoxy-prenylflavanone	C22H24O6	[M+Na]+
68	409.0	7.9E+03	Dioxocholenoic Acid	C24H34O4	[M+Na]+
69	413.1	1.3E+04	Rehmaionoside B	C19H34O8	[M+Na]+
70	417.0	2.7E+03	Hydroxyeicosatetraenoate glyceryl ester	C23H38O5	[M+H]+
71	419.0	2.3E+03	Palmitoyl glucuronide	C22H42O7	[M+H]+
72	421.1	5.6E+03	Tetracosanedioic acid	C24H46O4	[M+Na]+
73	425.0	5.0E+03	Octacosanoic acid	C28H56O2	[M+H]+
74	427.0	4.2E+03	Hydroxy-didehydrovitamin D3 lactone	C27H38O4	[M+H]+
75	429.1	3.9E+04	Isomontanolide	C22H30O7	[M+Na]+
76	433.1	3.5E+03	Hexacosenoic acid	C26H50O2	[M+K]+
77	435.1	3.7E+03	Ficisterol	C29H48O	[M+Na]+
78	437.0	7.3E+03	Tridecyl oleate	C29H56O2	[M+H]+

Table S7: Untargeted metabolic profiling of Goji Berries leaves organic extracts by ESI(+) Ion Trap MS[a]

#	m/z	Intensity	Putative Annotation[b]	Molecular formula (M)	Ion
79	439.0	5.3E+03	Calcitriol	C27H44O3	[M+Na]+
80	443.1	5.4E+03	Glycyphyllin	C21H24O9	[M+Na]+
81	445.1	6.8E+03	Trihydroxy-oxo-cholanoic Acid	C24H38O6	[M+Na]+
82	447.1	3.8E+03	Alpha-Tocotrienol	C29H44O2	[M+Na]+
83	449.1	6.6E+03	Luteolin 7-O-glucoside	C21H20O11	[M+H]+
84	453.0	1.7E+04	Tetradecyl-hexadecanoate	C30H60O2	[M+H]+
85	455.0	6.7E+03	Diaponeurosporenic acid	C30H40O2	[M+Na]+
86	457.0	1.0E+04	Heptamethoxyflavanone	C22H26O9	[M+Na]+
87	463.0	6.2E+03	Methylcycloartaenol	C31H52O	[M+Na]+
88	465.0	7.7E+03	Isoquercetin	C21H20O12	[M+H]+
89	467.0	4.4E+03	Dihydrophaseic acid O-beta-D-glucoside	C21H32O10	[M+Na]+
90	469.0	7.8E+03	Catechinol O-beta-D-galactopyranoside	C21H24O12	[M+H]+
91	471.0	4.1E+03	D-Galactosyl-3-(N-acetyl-beta-Dgalactosaminyl)-L serine	C17H30N2O13	[M+H]+
92	475.0	4.2E+03	Phylloquinol	C31H48O2	[M+Na]+
93	477.1	7.8E+03	Tetrahydrogeranylgeranyl diphosphate	C20H40O7P2	[M+Na]+
94	479.1	5.0E+03	Palmityl oleate	C32H62O2	[M+H]+
95	481.1	6.5E+03	Hexahydroxyflavone 3-glucoside	C21H20O13	[M+H]+
96	483.0	2.5E+03	6-Acetylpicropolin	C24H28O9	[M+Na]+
97	485.1	8.0E+03	Digalloyl-beta-D-glucose	C20H20O14	[M+H]+
98	487.1	3.9E+03	Tetramethyl-hexacosadienoic acid	C30H56O2	[M+K]+
99	493.0	1.7E+04	Limonin	C26H30O8	[M+Na]+
100	497.1	6.6E+03	PG(17:1/0:0)	C23H45O9P	[M+H]+
101	499.1	4.7E+03	PG(17:0/0:0)	C23H47O9P	[M+H]+
102	503.1	5.7E+03	Epi-Brassinolide	C28H48O6	[M+Na]+
103	507.1	6.2E+03	Stearyl oleate	C34H66O2	[M+H]+
104	509.1	2.5E+04	Trihydroxy-prenylchalcone O-glucoside	C26H30O9	[M+Na]+

Table S7: Untargeted metabolic profiling of Goji Berries leaves organic extracts by ESI(+) Ion Trap MS[a]

#	m/z	Intensity	Putative Annotation[b]	Molecular formula (M)	Ion
105	511.1	6.5E+03	PG(18:1(9Z)/0:0)	C24H47O9P	[M+H]+
106	513.1	4.3E+03	Raucaffricine	C27H32N2O8	[M+H]+
107	519.1	7.6E+03	Tetratriacontahexaenoic acid	C34H56O2	[M+Na]+
108	521.1	5.0E+03	Triacontanedioic acid	C30H58O4	[M+K]+
109	523.1	7.7E+03	Tetratriacontatetraenoic acid	C34H60O2	[M+Na]+
110	525.1	3.1E+04	Geranioloxyalatum flavone	C27H34O9	[M+Na]+
111	527.1	6.8E+03	Trisaccharide	C18H32O16	[M+Na]+
112	531.1	5.2E+03	Mucronine B	C28H36N4O4	[M+K]+
113	533.1	9.9E+03	DG(14:1/14:0/0:0)	C31H58O5	[M+Na]+
114	535.1	5.4E+03	Trihydroxyecdysone	C27H44O9	[M+Na]+
115	536.1	6.3E+03	PC(P-20:0/0:0)	C28H58NO6P	[M+H]+
116	537.1	5.7E+03	Phthioceranic acid (C36)	C36H72O2	[M+H]+
117	539.1	5.3E+03	L-Olivosyl-oleandolide	C26H44O10	[M+Na]+
118	544.0	3.0E+03	Ophrysanin	C23H21O14	[M+Na]+
119	545.1	5.6E+03	Trilobolide	C27H38O10	[M+Na]+
120	549.1	5.4E+03	Heneicosanyl oleate	C37H72O2	[M+H]+
121	551.1	4.2E+03	Tetradecenyl-octadecenoyl-sn-glycerol	C35H66O4	[M+H]+
122	557.1	3.5E+03	PG(20:3/0:0)	C26H47O9P	[M+Na]+
123	558.2	3.5E+03	Cyanidin (malonylglucoside)	C24H23O14	[M+Na]+
124	559.2	3.9E+03	DG(14:1/16:1/0:0)	C33H60O5	[M+Na]+
125	561.2	7.4E+03	DG(14:0/16:1/0:0)	C33H62O5	[M+Na]+
126	563.1	9.4E+03	DG(14:0/16:0/0:0)	C33H64O5	[M+Na]+
127	565.2	5.6E+03	Cortolone-3-glucuronide	C27H42O11	[M+Na]+
128	575.2	4.2E+03	DG(15:0/16:1/0:0)	C34H64O5	[M+Na]+
129	577.2	9.4E+03	DG(15:0/16:0/0:0)	C34H66O5	[M+Na]+
130	579.2	5.5E+03	PA(O-16:0/12:0)	C31H63O7P	[M+H]+

Table S7: Untargeted metabolic profiling of Goji Berries leaves organic extracts by ESI(+) Ion Trap MS[a]

#	m/z	Intensity	Putative Annotation[b]	Molecular formula (M)	Ion
131	587.2	5.0E+03	DG(14:0/18:2/0:0)	C35H64O5	[M+Na]+
132	591.2	6.4E+03	DG(14:0/18:0/0:0)	C35H68O5	[M+Na]+
133	593.1	5.0E+03	PA(O-16:0/13:0)	C32H65O7P	[M+H]+
134	599.2	5.1E+03	DG(15:0/18:3/0:0)	C36H64O5	[M+Na]+
135	601.2	9.8E+03	DG(15:0/18:2/0:0)	C36H66O5	[M+Na]+
136	603.2	6.1E+03	DG(15:0/18:1/0:0)	C36H68O5	[M+Na]+
137	605.2	7.9E+03	DG(15:0/18:0/0:0)	C36H70O5	[M+Na]+
138	607.2	5.8E+03	PA(O-16:0/14:0)	C33H67O7P	[M+H]+
139	611.2	3.3E+03	Rutin	C27H30O16	[M+H]+
140	613.3	6.9E+03	DG(14:0/20:3/0:0)	C37H66O5	[M+Na]+
141	615.3	7.7E+03	Myricetin (triacetylxyloside)	C26H24O15	[M+K]+
142	617.2	5.1E+03	PA(P-16:0/15:1)	C34H65O7P	[M+H]+
143	619.3	4.4E+03	O-[beta-D-glucopyranosyl]-furostantriol	C33H56O9	[M+Na]+
144	621.2	7.4E+03	PA(O-16:0/15:0)	C34H69O7P	[M+H]+
145	625.3	3.0E+03	Avermectin A2b aglycone	C34H50O9	[M+Na]+
146	629.2	1.0E+04	DG(14:0/20:3/0:0)	C37H66O5	[M+K]+
147	631.2	8.3E+03	DG(15:0/20:1/0:0)	C38H72O5	[M+Na]+
148	633.2	4.9E+03	DG(15:0/20:0/0:0)	C38H74O5	[M+Na]+
149	635.2	5.3E+03	DG(14:0/22:6/0:0)	C39H64O5	[M+Na]+
150	637.2	5.2E+03	DG(14:0/22:5/0:0)	C39H66O5	[M+Na]+
151	639.2	5.6E+03	DG(14:0/22:4/0:0)	C39H68O5	[M+Na]+
152	641.3	2.5E+03	DG(14:1/22:2/0:0)	C39H70O5	[M+Na]+
153	643.1	2.2E+03	DG(14:0/22:2/0:0)	C39H72O5	[M+Na]+
154	645.2	2.5E+03	DG(14:0/22:1/0:0)	C39H74O5	[M+Na]+
155	647.1	2.1E+03	DG(14:0/22:0/0:0)	C39H76O5	[M+Na]+
156	649.2	5.7E+03	PA(O-16:0/17:0)	C36H73O7P	[M+H]+

Table S7: Untargeted metabolic profiling of Goji Berries leaves organic extracts by ESI(+) Ion Trap MS[a]

#	m/z	Intensity	Putative Annotation[b]	Molecular formula (M)	Ion
157	651.3	6.9E+03	PI(P-20:0/0:0)	C29H57O11P	[M+K]+
158	653.1	3.2E+04	PG(12:0/15:0)	C33H65O10P	[M+H]+
159	655.2	8.3E+03	PA(O-16:0/18:4)	C37H67O7P	[M+H]+
160	657.2	4.8E+03	PA(O-16:0/18:3)	C37H69O7P	[M+H]+
161	659.3	3.3E+03	PA(O-16:0/18:2)	C37H71O7P	[M+H]+
162	661.2	2.7E+03	PA(O-16:0/18:1)	C37H73O7P	[M+H]+
163	663.2	3.7E+03	PA(16:0e/18:0)	C37H75O7P	[M+H]+
164	665.2	5.3E+03	DG(16:0/22:5/0:0)	C41H70O5	[M+Na]+
165	667.3	5.1E+03	PI(22:6/0:0)	C31H49O12P	[M+Na]+
166	669.1	3.6E+04	(O-galactopyranosyl)-keto-triacontanetriol	C36H70O9	[M+Na]+
167	679.1	2.8E+03	PG(12:0/17:1)	C35H67O10P	[M+H]+
168	681.2	1.9E+03	PG(12:0/17:0)	C35H69O10P	[M+H]+
169	685.2	4.9E+03	PA(O-16:0/20:3)	C39H73O7P	[M+H]+
170	689.1	8.6E+03	PA(O-16:0/20:1)	C39H77O7P	[M+H]+
171	691.2	2.7E+03	PA(O-16:0/20:0)	C39H79O7P	[M+H]+
172	693.2	3.9E+03	PG(12:0/18:1)	C36H69O10P	[M+H]+
173	695.2	2.4E+03	PG(12:0/18:0)	C36H71O10P	[M+H]+
174	703.1	2.8E+03	PA(14:0/22:1)	C39H75O8P	[M+H]+
175	707.2	2.0E+03	PG(12:0/19:1)	C37H71O10P	[M+H]+
176	715.2	2.2E+03	Scillaren A	C36H52O13	[M+Na]+
177	717.2	1.7E+03	PA(O-16:0/22:1)	C41H81O7P	[M+H]+
178	719.2	2.0E+03	PA(O-16:0/22:0)	C41H83O7P	[M+H]+
179	721.3	2.0E+03	PG(12:0/20:1)	C38H73O10P	[M+H]+
180	735.3	2.2E+03	PG(13:0/20:1)	C39H75O10P	[M+H]+
181	737.3	3.3E+03	O-glucopyranosyl-(hexadecanoyloxy)-eicosanol	C42H82O8	[M+Na]+
182	749.2	2.0E+03	Hexadecanoyl-(octadecenoyl)-sn-glycerophosphosn-glycerol	C40H77O10P	[M+H]+

Table S7: Untargeted metabolic profiling of Goji Berries leaves organic extracts by ESI(+) Ion Trap MS[a]

#	m/z	Intensity	Putative Annotation[b]	Molecular formula (M)	Ion
183	767.2	1.7E+03	PG(P-18:0/17:2)	C41H77O9P	[M+Na]+
184	770.3	7.4E+03	GalCer(d16:1/23:0)	C45H87NO8	[M+H]+
185	771.2	3.1E+03	PA(19:0/22:2)	C44H83O8P	[M+H]+
186	773.2	1.6E+03	DG(22:6/24:1/0:0)	C49H82O5	[M+Na]+
187	781.2	2.5E+03	PI(12:0/18:1)	C39H73O13P	[M+H]+
188	785.2	2.2E+04	PA(20:0/22:2)	C45H85O8P	[M+H]+
189	787.2	7.8E+03	PA(20:0/22:1)	C45H87O8P	[M+H]+
190	796.3	4.0E+03	GalCer(d18:2/23:0)	C47H89NO8	[M+H]+
191	797.3	1.6E+04	TG(16:1/14:0/16:1)	C49H90O6	[M+Na]+
192	798.3	7.2E+03	GalCer(d18:1/23:0)	C47H91NO8	[M+H]+
193	799.3	3.6E+03	TG(16:0/14:0/16:1)	C49H92O6	[M+Na]+
194	802.3	1.3E+03	PE(16:0/22:6)	C43H74NO9P	[M+Na]+
195	803.3	3.4E+03	PA(21:0/22:0)	C46H91O8P	[M+H]+
196	812.3	2.4E+03	PE(20:4/22:6)	C47H74NO8P	[M+H]+
197	813.2	2.4E+04	PA(22:0/22:2)	C47H89O8P	[M+H]+
198	814.2	1.1E+04	PA(22:0/22:0)	C47H93O8P	[M+H]+
199	871.5	1.9E+03	Pheophytin a	C55H74N4O5	[M+H]+
200	893.3	1.1E+03	Chlorophyll a	C55H72O5N4Mg	[M+H]+
201	909.2	8.9E+02	Pheophytin a	C55H74N4O5	[M+K]+
202	915.3	6.4E+02	Chlorophyll a	C55H72O5N4Mg	[M+Na]+

a Experiments carried out by ESI(+) Esquire 6000 MS

b MG Monoacylglycerol; DG Diacylglycerol; TG Triacylglycerol; GalCer Galactosylceramide; PA Phosphatidic acid; PC Phosphatidylcholine; PE Phosphatidylethanolamine; PG Glycerophospholipids; PI Phosphatidylinositol.

Table S8: Untargeted metabolic profiling of Goji Berries leaves organic extracts by ESI(-) Ion Trap MS[a]

#	m/z	Intensity	Putative Annotation[b]	Molecular Formula (M)	Ion
1	110.8	1.6E+03	2-Furoic acid	C5H4O3	[M-H]-
2	127.8	3.3E+03	Pyroglutamic acid	C5H7NO3	[M-H]-
3	133.8	1.1E+03	Malic acid	C4H6O5	[M-H]
4	144.8	9.4E+02	Glutamine	C5H10N2O3	[M-H]-
5	156.8	2.1E+03	Methyl-octanoic acid	C9H18O2	[M-H]-
6	158.7	1.7E+03	Pimelic acid	C7H12O4	[M-H]-
7	160.7	1.7E+03	Hydroxyadipic acid	C6H10O5	[M-H]-
8	168.8	2.8E+03	Citronellic acid	C10H18O2	[M-H]-
9	170.9	3.4E+03	Decanoic acid	C10H20O2	[M-H]-
10	172.8	1.9E+03	Shikimic acid	C7H10O5	[M-H]-
11	178.8	9.7E+02	Aldohexose	C6H12O6	[M-H]-
12	184.8	1.2E+03	Tartaric acid	C4H6O6	[M+Cl]-
13	185.9	3.3E+03	hydroxy-2-decenoic acid	C10H18O3	[M-H]-
14	186.9	3.3E+03	Hydroxy-decanoic acid	C10H20O3	[M-H]-
15	200.8	1.6E+03	Sebacic acid	C10H18O4	[M-H]-
16	204.9	1.2E+03	Methylcitric acid	C7H10O7	[M-H]-
17	210.8	9.2E+02	Methyl-dodecenoic acid	C13H24O2	[M-H]-
18	212.9	3.1E+03	Methyl-dodecanoic acid	C13H26O2	[M-H]-
19	214.9	9.4E+02	Hexose	C6H1206	[M+Cl]-
20	225.0	4.3E+03	Myristoleic acid	C14H26O2	[M-H]-
21	227.0	7.6E+04	Myristic acid	C14H28O2	[M-H]-
22	229.0	1.6E+03	Dodecanedioic acid	C12H22O4	[M-H]-
23	239.0	5.9E+03	Tridecenyl acetate	C15H28O2	[M-H]-
24	241.0	2.7E+04	Methyl myristic acid	C15H30O2	[M-H]-
25	242.9	1.3E+03	Methyl-dodecanedioic acid	C13H24O4	[M-H]-

Table S8: Untargeted metabolic profiling of Goji Berries leaves organic extracts by ESI(-) Ion Trap MS[a]

#	m/z	Intensity	Putative Annotation[b]	Molecular Formula (M)	Ion
26	250.9	1.3E+03	Hexadecynoic acid	C16H28O2	[M-H]-
27	253.0	4.5E+04	Palmitoleic acid	C16H30O2	[M-H]-
28	255.0	1.0E+05	Palmitic acid	C16H32O2	[M-H]-
29	256.9	2.3E+03	Tetradecanedioic acid	C14H26O4	[M-H]-
30	264.9	4.0E+04	Methyl-Hexadecadienoic acid	C17H30O2	[M-H]-
31	267.0	1.3E+04	Methyl-hexadecenoic acid	C17H32O2	[M-H]-
32	269.0	1.4E+04	Methyl palmitic acid	C17H34O2	[M-H]-
33	271.0	1.7E+03	Hydroxy palmitic acid	C16H32O3	[M-H]-
34	276.9	5.8E+03	Linolenic acid	C18H30O2	[M-H]-
35	279.0	1.2E+04	Linoleic acid	C18H32O2	[M-H]-
36	281.0	3.5E+04	Oleic acid	C18H34O2	[M-H]-
37	283.0	5.2E+04	Stearic acid	C18H36O2	[M-H]-
38	285.0	2.2E+03	Hydroxy-palmitic acid methyl ester	C17H34O3	[M-H]-
39	292.9	1.3E+04	Methyl-octadecadienoic acid	C19H34O2	[M-H]-
40	295.0	3.2E+03	Methyl-octadecenoic acid	C19H36O2	[M-H]-
41	297.0	6.2E+03	Methyl stearic acid	C19H38O2	[M-H]-
42	299.0	1.7E+03	Hydroxystearic acid	C18H36O3	[M-H]-
43	305.0	2.4E+03	Linolenic acid, ethyl ester	C20H34O2	[M-H]-
44	307.0	1.7E+03	Eicosadienoic acid	C20H36O2	[M-H]-
45	309.0	1.1E+04	Eicosenoic acid	C20H38O2	[M-H]-
46	311.0	1.3E+04	Arachidic acid	C20H40O2	[M-H]-
47	312.9	1.6E+03	Dihydroxy-octadecenoic acid	C18H34O4	[M-H]-
48	314.9	1.4E+03	Dihydroxy-octadecanoic acid	C18H36O4	[M-H]-
49	321.0	1.8E+03	Eicosadienoic acid, methyl ester	C21H38O2	[M-H]-
50	322.9	1.9E+03	Eicosenoic acid, methyl ester	C21H40O2	[M-H]-
51	325.0	1.1E+04	Eicosanoic acid, methyl ester	C21H42O2	[M-H]-

Table S8: Untargeted metabolic profiling of Goji Berries leaves organic extracts by ESI(-) Ion Trap MS[a]

#	m/z	Intensity	Putative Annotation[b]	Molecular Formula (M)	Ion
52	326.9	2.3E+03	Hydroxy-eicosanoic acid	C20H40O3	[M-H]-
53	337.0	4.8E+03	Docosenoic acid	C22H42O2	[M-H]-
54	339.0	8.5E+03	Docosanoic acid (22:0)	C22H44O2	[M-H]-
55	340.9	2.6E+03	Eicosanedioic acid	C20H38O4	[M-H]-
56	345.8	9.1E+02	Deoxyguanosine 5'-monophosphate	C10H14N5O7P	[M-H]-
57	353.0	7.1E+03	Chlorogenic acid	C16H18O9	[M-H]-
58	355.0	1.6E+03	Hydroxydocosanoic acid	C22H44O3	[M-H]
59	365.0	9.1E+02	Docosenyl acetate	C24H46O2	[M-H]-
60	367.1	4.3E+03	Tetracosanoic acid	C24H48O2	[M-H]-
61	369.1	1.7E+03	Docosanedioic acid	C22H42O4	[M-H]-
62	371.1	1.3E+03	Deoxyoleandolide	C20H36O6	[M-H]-
63	381.1	3.6E+03	Heptadecenyl-resorcinol	C23H38O2	[M+Cl]-
64	383.1	1.7E+03	Sativic acid	C18H36O6	[M+Cl]-
65	395.2	2.7E+03	Methyl-hexacosanoic acid	C26H52O2	[M-H]-
66	397.0	5.1E+03	Tetracosanedioic acid	C24H46O4	[M-H]-
67	399.0	1.1E+03	Didecanoylglycerol	C23H44O5	[M-H]-
68	411.1	1.4E+03	Hydroxyhexacosanoic acid	C26H52O3	[M-H]-
69	415.0	9.6E+02	Calcitriol	C27H44O3	[M-H]-
70	421.0	1.0E+03	Octacosenoic acid	C28H54O2	[M-H]
71	425.1	2.4E+03	Hexacosanedioic acid	C26H50O4	[M-H]-
72	431.0	1.2E+03	Calcitetrol	C27H44O4	[M-H]-
73	439.2	1.5E+03	Hydroxy-octacosanoic acid	C28H56O3	[M-H]-
74	441.1	2.5E+03	N-linolenoyl-glutamine	C23H38N2O4	[M+Cl]-
75	451.3	1.1E+03	6-C-Glucopyranosylcatechin	C21H24O11	[M-H]-
76	465.1	1.2E+03	Methyl-triacontanoic acid	C31H62O2	[M-H]-
77	467.2	1.0E+03	Catechinol O-galactopyranoside	C21H24O12	[M-H]-

Table S8: Untargeted metabolic profiling of Goji Berries leaves organic extracts by ESI(-) Ion Trap MS[a]

#	m/z	Intensity	Putative Annotation[b]	Molecular Formula (M)	Ion
78	487.1	5.9E+03	Fucosyllactose	C18H32O15	[M-H]-
79	529.2	1.0E+03	Luteolin glucoside-4'-(methyl-butenoate)	C26H26O12	[M-H]-
80	623.2	9.3E+02	Kanokoside D	C27H44O16	[M-H]-
81	665.2	2.8E+03	Maltotetraose	C24H42O21	[M-H]
82	685.2	2.3E+03	Glucopyranosyl-(tetradecanoyloxy)- eicosanol	C40H78O8	[M-H]-
83	695.3	2.5E+03	Phylloflavanine	C35H32O13	[M+Cl]
84	741.3	2.6E+03	Quercetin glucosyl-xylosyl-rhamnoside	C32H38O20	[M-H]-

a Experiments carried out by ESI(+) Esquire 6000 MS

b MG Monoacylglycerol; DG Diacylglycerol; TG Triacylglycerol; GalCer Galactosylceramide; PA Phosphatidic acid; PC Phosphatidylcholine; PE Phosphatidylethanolamine; PG Glycerophospholipids; PI Phosphatidylinositol.
